# Supplementary material for: Pattern and prevalence of vaping nicotine and non-nicotine drugs in the United Kingdom: a cross-sectional study
Source: BMJ Open. 2023 Apr 25;13(4):e066826. doi: 10.1136/bmjopen-2022-066826 (PMC10151839; doi:10.1136/bmjopen-2022-066826)
Supplement: Supplementary data [file bmjopen-2022-066826supp002.pdf]

Background Info

INFORMATION SHEET FOR PARTICIPANTS
Ethical Clearance Reference Number: HR-21/22-26249

Title of study : A survey about vaping drugs in the UK

Invitation Paragraph

I would like to invite you to participate in this research project. Before you decide whether you want to take part, it is important for you to understand why the research is being done and what your participation will involve. Please take time to read the following information carefully and discuss it with others if you wish. My contact details are at the end of this information sheet if you need to ask any questions before you decide to take part.

What is the purpose of the study?

The purpose of the study is to find out how common vaping is. In recent years more and more people have begun to vape nicotine and drugs that do not contain nicotine such as cannabis. People have been switching from smoking drugs to vaping drugs for several reasons e.g. it is thought to be a safer way of taking drugs. We are interested to know how common this is and how regular people vape drugs that do not contain nicotine and their reasons for vaping drugs instead of other ways of taking drugs. This will help our team of researchers, who study people who use different types of drugs, to have a better understanding of people's vaping behaviours.

Why have I been invited to take part?

You are being invited to participate in this study because you are registered with Prolific and they have identified that you meet the inclusion criteria. Our inclusion criteria is very simple, it includes anyone over the age of 18. So you don't have to have experience of vaping either nicotine or non-nicotine drugs to take part. We are trying to understand how common vaping in is the population, so it's important we get a snap shot of people who do and do not vape drugs.

What will happen if I take part?

If you agree to take part you will complete a survey anonymously. The survey will ask you questions about whether you have smoked or vaped nicotine or non-nicotine drugs and if you currently do so. If you do vape, we'll ask you questions about what substances; for example *have you ever used any type of vaping device? If you have, which of the following substances have you ever vaped? cannabis, alcohol, cocaine etc, how old were you when you first vaped each substance?* The survey will take you approximately 5-10 mins to complete, depending on your experience of vaping. We will not be asking for your name, date of birth or any other personal data that can identify you. We will ask your age category, eg 18-24, 25-34 and your sex.

Do I have to take part?

Participation is completely voluntary. You should only take part if you want to and choosing not to take part will not disadvantage you in anyway. If you choose to take part you will be asked to provide your consent. To do this you will be asked to indicate that you have read and understand the information provided and that you consent to your anonymous data being used for the purposes explained. You are free to withdraw at any point during completion of the survey, without having to give a reason. You can delete your responses to the questions before exiting the survey. You will be asked if you are sure you want to submit your answers when you reach the end of the questionnaire. Incomplete questionnaires will be unable to be submitted. Withdrawing from the study will not affect you in any way. Once you submit the survey, it will no longer be possible to withdraw from the study because the data will be fully anonymous. Please do not include any personal identifiable information in your responses.

Incentives

The usual Prolific rewards applies to this survey. Participants will be paid £7.56 per hour and and we expect the survey to take 5-10 mins.

Roberts E, *et al. BMJ Open* 2023; 13:e066826. doi: 10.1136/bmjopen-2022-066826

What are the possible risks of taking part?

We do not forsee any possible risks of taking part as this survey is completely anonymous. If however you feel uncomfortable or would like to speak with a trained person about any drug use, we have provided contact details of national helplines at the end of the survey. Data handling and confidentiality This research is anonymous. Prolific do not hold any data related to the survey, and the researchers will not have access to the any identifiable information that Prolific hold. This means that nobody, including the researchers, will be aware of your identity, and nobody will be able to connect you to the answers you provide, even indirectly. Your answers will nevertheless be treated confidentially and the information you provide will not allow you to be identified in any research outputs/publications. Your data will be held securely in on King's College London password protected and encrypted computer data storage drive. All data will only be available to the researchers and will be handled in accordance with the UK General Data Protection Regulation (UK GDPR) and the Data Protection Act 2018. Data will be kept within the research team and not shared with a third party. All data will be kept in line with the King's College London data retention schedule and securely disposed of within 4 years.

How is the project being funded?

This study is being funded by King's College London and the Maudsley Biomedical Research Centre.

What will happen to the results of the study?

The results of the study will be summarised in a report for a journal article, which we plan to publish. The anonymous data will be kept within the research team and not shared with a third party.

Who should I contact for further information?

If you have any questions or require more information about this study, please contact me using the following contact details: deborah.j.robson@kcl.ac.uk

What if I have further questions, or if something goes wrong?

If this study has harmed you in any way or if you wish to make a complaint about the conduct of the study you can contact King's College London using the details below for further advice and information: The Chair, Health Faculties Research Ethics Sub-Committee rec@kcl.ac.uk

Thank you for reading this information sheet and for considering taking part in this research.

Consent

I confirm that I have read and understood the online information sheet presented dated 20/01/22, version #3, for the above project and I agree to take part in this research project

- ◯ Yes
- ◯ No

I consent voluntarily to be a participant in this project and understand that I can refuse to take part and can withdraw from the project before I submit my answers to the survey questions, at any time, without having to give a reason

- ◯ Yes
- ◯ No

I consent to the processing of my personal information for the purposes explained to me in the online Information Sheet. I understand that such information will be handled under the terms of UK data protection law, including the UK General Data Protection Regulation (UK GDPR) and the Data Protection Act 2018

- Yes
- No

I understand that my information may be subject to review by responsible individuals from King's College London for monitoring and audit purposes.

- Yes
- No

I understand that confidentiality and anonymity will be maintained, and it will not be possible to identify me in any research outputs

- Yes
- No

I understand that I must not take part if I fall under the exclusion criteria as detailed in the information sheet and explained to me by the researcher in the online information sheet.

- Yes
- No

Block 62

What is your prolific ID?

Screening

How often, if at all, do you currently smoke ordinary tobacco cigarettes (either factory-made/packet or roll-your-own)?

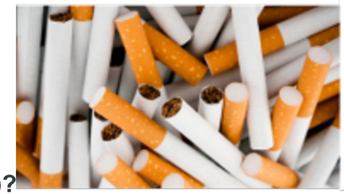

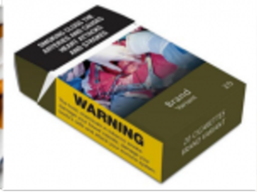

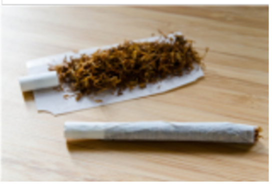

- Daily

- Less than daily, but at least once a week
- Less than weekly, but at least once a month
- Less than monthly, but occasionally
- I have quit smoking
- I have never been a smoker

Have you EVER used an electronic or e-cigarette?

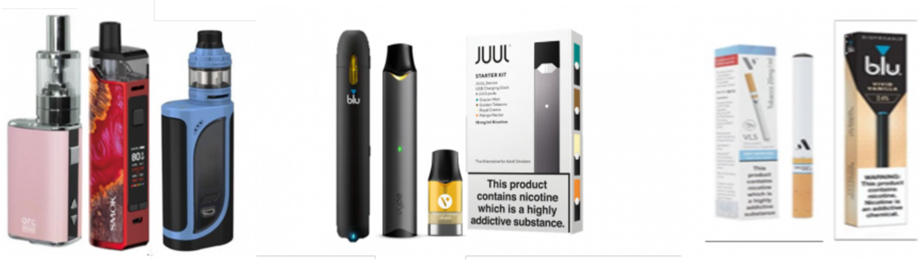

- Yes
- No

Have you EVER used electronic devices to vapourise liquids?

- Yes
- No

Have you EVER use an electronic devices to vapourise or dry herbs?

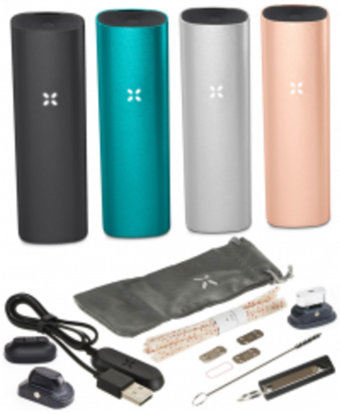

- Yes
- No

Have you EVER used an electronic table-top vaporizer?

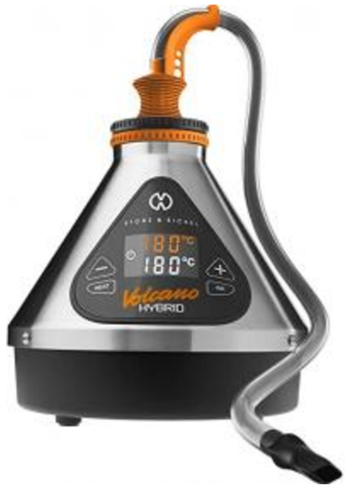

- Yes
- No

Substance use

You said that you have used an electronic vaping device. Which of the following substances have you EVER vaped using an electronic vaping device? (tick all that apply)

- Nicotine (i.e., nicotine e-liquid, salts etc.)
- Alcohol (i.e., alcohol in pure form, not as a flavouring or an additive)
- Caffeine (i.e., caffeine in pure form, not as a flavouring or an additive)
- Products containing cannabis (i.e., cannabis oils, cannabis concentrate, cannabis resin, THC, cannabidiol, CBD, butane hash oil, hash, skunk, dope, weed, pot, etc.)
- Synthetic cannabis products (i.e., spice, SCRA, black mamba etc.)
- Cocaine (i.e., powder, crack, rocks, coke, charlie etc.)
- MDMA (i.e., ecstasy, Mandy, methylenedioxymethamphetamine etc.)
- GBL (i.e., 'G', gamma-hydroxybutyrate, GHB, gamma-butyrolactone, liquid ecstasy etc.)
- Amphetamines (i.e., crystal methamphetamine, crystal, meth, ice, tina, speed, china white, ritalin, methylphenidate etc.)
- Opioids (i.e., heroin, fentanyl, morphine etc.)
- Ketamine (i.e., Special K, Ket etc.)
- Mephedrone (i.e., M-CAT, meow-meow etc.)
- DMT (i.e, dimethyltryptamine, ayahuasca etc.)
- Psilocybin (i.e., magic mushrooms, shrooms)
- Benzodiazepines (i.e, benzos, blues, xanax, vallium, rivitrol, diazepam, tempazepan, clonazepam etc.)
- 2C's (i.e, 2CB, 2CE, Seventh-Heaven etc.)
- alpha-PVP (i.e., alpha-pyrrolidinopentiophenone, flakka, gravel etc.)
- NBOMe (i.e, 2, 5-dimethoxy-4-bromophenethylamine, N-Bomb etc.)
- Other- please describe

Vaping Nicotine

You said that you had vaped nicotine. What type of nicotine have you vaped? (tick all that apply)

- ☐ Nicotine e-liquid (freebase)
- ☐ Nicotine salts
- ☐ Other, please describe

How old were you the first time you ever used nicotine? (by ANY method not only using an electronic vaping device)

What was the first route of administration by which you ever used nicotine?

- ○ Vaping
- ○ Smoking
- ○ Oral (ingesting by mouth)
- ○ Intravenous (injecting into a vein)
- ○ Sniffing/snorting (taking by nose)
- ○ Other, please describe

How old were you when you the first time you ever vaped nicotine?

What was your main reason for vaping nicotine the first time?

- ○ To quit smoking tobacco
- ○ To cut down smoking tobacco
- ○ To use when I could not or was not allowed to smoke tobacco
- ○ To avoid returning to smoking tobacco
- ○ Because I thought I would enjoy it
- ○ Because it I thought it would give me a more pleasurable effect than other routes
- ○ Because I was attracted to the different flavours you can get in nicotine e-liquid
- ○ Because it is less harmful than smoking tobacco

- Because I was concerned about my health
- Curiosity just wanted to try them
- To save money
- To protect others from second-hand exposure
- For some other reason -please explain
- Don't know
- Prefer not to say

What were any other reasons for vaping nicotine the first time?

- To quit smoking tobacco
- To cut down smoking tobacco
- To use when I cannot or am not allowed to smoke tobacco
- To avoid returning to smoking tobacco
- Because I enjoy it
- Because it gives me a more pleasurable effect than other routes
- Because I like the different flavours you can get in nicotine e-liquids
- Because it is less harmful than smoking tobacco
- Because I was concerned about my health
- Curiosity just wanted to try them
- To save money
- To protect others from second-hand exposure
- For some other reason -please explain
- Don't know
- Prefer not to say

Have you used nicotine in the last 30 days?

- Yes
- No

How did you use nicotine in past 30 days?

	Yes	No
Smoked	☐	☐
Vaped	☐	☐
Nicotine replacement therapy	☐	☐
Nicotine mouth pouches	☐	☐

Other source of nicotine (give details) ☐	☐	☐

Past 30 Nicotine

Do you think your use of nicotine is having a negative effect on your mental or physical health?

- ◯ Yes, on both my mental and physical health
- ◯ Yes, on my physical health only
- ◯ Yes, on my mental health only
- ◯ No

Which of the following best describes the frequency you used nicotine using any route of administration in the last 30 days?

- ◯ Daily
- ◯ Less than daily, but at least once a week
- ◯ Less than weekly, but at least once a month

Do you have any current intention to quit using nicotine?

	Yes	No	Not applicable
Quit smoking tobacco	☐	☐	☐
Quit vaping nicotine	☐	☐	☐
Quit Nicotine replacement therapy	☐	☐	☐
Quit Nicotine mouth pouches	☐	☐	☐
Quit other source of nicotine	☐	☐	☐

Have you used an electronic vaping device to use nicotine in the last 30 days?

- ◯ Yes
- ◯ No

Current Use Nicotine

What is your main reason for vaping nicotine now?

- ◯ To quit smoking tobacco
- ◯ To cut down smoking tobacco
- ◯ To use when I cannot or am not allowed to smoke tobacco
- ◯

- To avoid returning to smoking tobacco
- Because I enjoy it
- Because it gives me a more pleasurable effect than other routes
- Because it is less harmful than smoking tobacco
- Because I was concerned about my health
- Curiosity just wanted to try them
- To save money
- To protect others from second-hand exposure
- For some other reason -please explain
- Don't know
- Prefer not to say

What are any other reasons for vaping nicotine now?

- To quit smoking tobacco
- To cut down smoking tobacco
- To use when I cannot or am not allowed to smoke tobacco
- To avoid returning to smoking tobacco
- Because I enjoy it
- Because it gives me a more pleasurable effect than other routes
- Because it is less harmful than smoking tobacco
- Because I was concerned about my health
- Curiosity just wanted to try them
- To save money
- To protect others from second-hand exposure
- For some other reason -please explain
- Don't know
- Prefer not to say

Which of the following best describes the frequency you vaped nicotine in the last 30 days?

- Daily
- Less than daily, but at least once a week
- Less than weekly, but at least once a month

What is the main electronic vaping device by which you use nicotine?

- Commercially bought electronic or e-cigarette
- Commercially bought electronic device used to vaporise dry herbs
- Commercially bought tabletop vapouriser
- Commercially bought electronic device used to vape liquids (but not an e-cigarette or a tabletop vapouriser)
- Homemade electronic device
- Other, please describe

Are there any other electronic vaping devices by which you use nicotine?

- Commercially bought electronic or e-cigarette
- Commercially bought electronic device used to vaporise dry herbs
- Commercially bought tabletop vapouriser
- Commercially bought electronic device used to vape liquids (but not an e-cigarette or a tabletop vapouriser)
- Homemade electronic device
- Other, please describe

Vaping caffine

You said that you had vaped caffeine. What type of caffeine have you vaped? (tick all that apply)

- Caffeine
- Other, please describe

How old were you the first time you ever used caffeine? (by ANY method not only using an electronic vaping device)

What was the first route of administration by which you ever used caffeine?

- Vaping
- Smoking
- Oral (ingesting by mouth)
- Intravenous (injecting into a vein)
- Sniffing/snorting (taking by nose)
- Other, please describe

Roberts E, *et al. BMJ Open* 2023; 13:e066826. doi: 10.1136/bmjopen-2022-066826

How old were you when you the first time you ever vaped caffeine?

What was your main reason for vaping caffeine the first time?

- To quit other routes of administration
- To cut down other routes of administration
- To use when I could not or was not allowed to use via other routes of administration
- To avoid returning to other routes of administration
- Because I thought I would enjoy it
- Because I though it would give me a more pleasurable effect than other routes
- Because they are less harmful than other routes of administration
- Because I was concerned about my health
- Curiosity just wanted to try them
- To save money
- To protect others from second-hand exposure
- For some other reason -please explain
- Don't know
- Prefer not to say

What were any other reasons for vaping caffeine the first time?

- To quit other routes of administration
- To cut down other routes of administration
- To use when I cannot or am not allowed to use via other routes of administration
- To avoid returning to other routes of administration
- Because I enjoy it
- Because it gives me a more pleasurable effect than other routes
- Because they are less harmful than other routes of administration
- Because I was concerned about my health
- Curiosity just wanted to try them
- To save money
- To protect others from second-hand exposure
- For some other reason -please explain
- Don't know
- Prefer not to say

Have you used caffeine in the last 30 days?

- Yes
- No

Past 30 Caffeine

Do you think your use of caffeine is having a negative effect on your mental or physical health?

- Yes, on both my mental and physical health
- Yes, on my physical health only
- Yes, on my mental health only
- No

Which of the following best describes the frequency you used caffeine using any route of administration in the last 30 days?

- Daily
- Less than daily, but at least once a week
- Less than weekly, but at least once a month

Do you have any current intention to quit using caffeine?

- Yes
- No

Have you used an electronic vaping device to use caffeine in the last 30 days?

- Yes
- No

Current Use Caffeine

What is your main reason for vaping caffeine now?

- To quit other routes of administration
- To cut down other routes of administration

- To use when I cannot or am not allowed to use via other routes of administration
- To avoid returning to other routes of administration
- Because I enjoy it
- Because it gives me a more pleasurable effect than other routes
- Because they are less harmful than other routes of administration
- Because I was concerned about my health
- Curiosity just wanted to try them
- To save money
- To protect others from second-hand exposure
- For some other reason -please explain
- Don't know
- Prefer not to say

What are any other reasons for vaping caffeine now?

- To quit other routes of administration
- To cut down other routes of administration
- To use when I cannot or am not allowed to use via other routes of administration
- To avoid returning to other routes of administration
- Because I enjoy it
- Because it gives me a more pleasurable effect than other routes
- Because they are less harmful than other routes of administration
- Because I was concerned about my health
- Curiosity just wanted to try them
- To save money
- To protect others from second-hand exposure
- For some other reason -please explain
- Don't know
- Prefer not to say

Which of the following best describes the frequency you vaped caffeine in the last 30 days?

- Daily
- Less than daily, but at least once a week
- Less than weekly, but at least once a month

What is the main electronic vaping device by which you use caffeine?

- Commercially bought electronic or e-cigarette
- Commercially bought electronic device used to vaporise dry herbs
- Commercially bought tabletop vapouriser
- Commercially bought electronic device used to vape liquids (but not an e-cigarette or a tabletop vapouriser)
- Homemade electronic device
- Other, please describe

What is the main electronic vaping device by which you use caffeine?

- Commercially bought electronic or e-cigarette
- Commercially bought electronic device used to vaporise dry herbs
- Commercially bought tabletop vapouriser
- Commercially bought electronic device used to vape liquids (but not an e-cigarette or a tabletop vapouriser)
- Homemade electronic device
- Other, please describe

What is the main electronic vaping device by which you use caffeine?

- Commercially bought electronic or e-cigarette
- Commercially bought electronic device used to vaporise dry herbs
- Commercially bought tabletop vapouriser
- Commercially bought electronic device used to vape liquids (but not an e-cigarette or a tabletop vapouriser)
- Homemade electronic device
- Other, please describe

Are there any other electronic vaping devices by which you use caffeine?

- Commercially bought electronic or e-cigarette
- Commercially bought electronic device used to vaporise dry herbs
- Commercially bought tabletop vapouriser
- Commercially bought electronic device used to vape liquids (but not an e-cigarette or a tabletop vapouriser)
- Homemade electronic device
- Other, please describe

Vaping Alcohol

You said that you had vaped alcohol. What type of alcohol have you vaped? (tick all that apply)

- Alcohol liquid
- Other, please describe

How old were you the first time you ever used alcohol? (by ANY method not only using an electronic vaping device)

What was the first route of administration by which you ever used alcohol?

- Vaping
- Oral (ingesting by mouth)
- Intravenous (injecting into a vein)
- Sniffing/snorting (taking by nose)
- Other, please describe

How old were you when you the first time you ever vaped alcohol?

What was your main reason for vaping alcohol the first time?

- To quit other routes of administration
- To cut down other routes of administration
- To use when I could not or was not allowed to use via other routes of administration
- To avoid returning to other routes of administration
- Because I thought I would enjoy it
- Because I though it would give me a more pleasurable effect than other routes
- Because they are less harmful than other routes of administration
- Because I was concerned about my health
- Curiosity just wanted to try them
- To save money
- To protect others from second-hand exposure
- For some other reason -please explain

- Don't know
- Prefer not to say

What were any other reasons for vaping alcohol the first time?

- To quit other routes of administration
- To cut down other routes of administration
- To use when I cannot or am not allowed to use via other routes of administration
- To avoid returning to other routes of administration
- Because I enjoy it
- Because it gives me a more pleasurable effect than other routes
- Because it is less harmful than other routes of administration
- Because I was concerned about my health
- Curiosity just wanted to try them
- To save money
- For some other reason -please explain
- Don't know
- Prefer not to say

Have you used alcohol in the last 30 days?

- Yes
- No

Past 30 Alcohol

Do you think your use of alcohol is having a negative effect on your mental or physical health?

- Yes, on both my mental and physical health
- Yes, on my physical health only
- Yes, on my mental health only
- No

Which of the following best describes the frequency you used alcohol using any route of administration in the last 30 days?

- Daily
- Less than daily, but at least once a week

- Less than weekly, but at least once a month

Do you have any current intention to quit using alcohol?

- Yes
- No

Have you used an electronic vaping device to use alcohol in the last 30 days?

- Yes
- No

Current alcohol use

What is your main reason for vaping alcohol now?

- To quit other routes of administration
- To cut down other routes of administration
- To use when I cannot or am not allowed to use via other routes of administration
- To avoid returning to other routes of administration
- Because I enjoy it
- Because it gives me a more pleasurable effect than other routes
- Because it is less harmful than other routes of administration
- Because I was concerned about my health
- Curiosity just wanted to try them
- To save money
- For some other reason -please explain
- Don't know
- Prefer not to say

What are any other reasons for vaping alcohol now?

- To quit other routes of administration
- To cut down other routes of administration
- To use when I cannot or am not allowed to use via other routes of administration
- To avoid returning to other routes of administration
- Because I enjoy it
- Because it gives me a more pleasurable effect than other routes

- ☐ Because it is less harmful than other routes of administration
- ☐ Because I was concerned about my health
- ☐ Curiosity just wanted to try them
- ☐ To save money
- ☐ For some other reason -please explain
- ☐ Don't know
- ☐ Prefer not to say

Which of the following best describes the frequency you vaped alcohol in the last 30 days?

- ○ Daily
- ○ Less than daily, but at least once a week
- ○ Less than weekly, but at least once a month

What is the main electronic vaping device by which you use alcohol?

- ○ Commercially bought electronic or e-cigarette
- ○ Commercially bought electronic device used to vaporise dry herbs
- ○ Commercially bought tabletop vapouriser
- ○ Commercially bought electronic device used to vape liquids (but not an e-cigarette or a tabletop vapouriser)
- ○ Homemade electronic device
- ○ Other, please describe

Are there any other electronic vaping devices by which you use alcohol?

- ☐ Commercially bought electronic or e-cigarette
- ☐ Commercially bought electronic device used to vaporise dry herbs
- ☐ Commercially bought tabletop vapouriser
- ☐ Commercially bought electronic device used to vape liquids (but not an e-cigarette or a tabletop vapouriser)
- ☐ Homemade electronic device
- ☐ Other, please describe

Vaping Cannabis

You said that you had vaped cannabis. What type of cannabis have you vaped? (tick all that apply)

- ☐ Combined THC/CBD resin

- THC resin
- CBD resin
- Combined THC/CBD concentrate
- THC concentrate
- CBD concentrate
- Combined THC/CBD oil
- THC oil
- CBD oil
- Butane hash oil
- Dry herb/bud combined THC/CBD
- Dry herb/bud THC
- Dry herb/bud CBD
- Other, please describe

How old were you the first time you ever used cannabis? (by ANY method not only using an electronic vaping device)

What was the first route of administration by which you ever used cannabis?

- Vaping
- Smoking
- Oral (ingesting by mouth)
- Intravenous (injecting into a vein)
- Sniffing/snorting (taking by nose)
- Other, please describe

How old were you when you the first time you ever vaped cannabis?

What was your main reason for vaping cannabis the first time?

- To quit other routes of administration
- To cut down other routes of administration
- To use when I could not or was not allowed to use via other routes of administration

- To avoid returning to other routes of administration
- Because I thought I would enjoy it
- Because I though it would give me a more pleasurable effect than other routes
- Because they are less harmful than other routes of administration
- Because I was concerned about my health
- Curiosity just wanted to try them
- To save money
- To protect others from second-hand exposure
- For some other reason -please explain
- Don't know
- Prefer not to say

What were any other reasons for vaping cannabis the first time?

- To quit other routes of administration
- To cut down other routes of administration
- To use when I cannot or am not allowed to use via other routes of administration
- To avoid returning to other routes of administration
- Because I enjoy it
- Because it gives me a more pleasurable effect than other routes
- Because they are less harmful than other routes of administration
- Because I was concerned about my health
- Curiosity just wanted to try them
- To save money
- To protect others from second-hand exposure
- For some other reason -please explain
- Don't know
- Prefer not to say

Have you used cannabis in the last 30 days?

- Yes
- No

Past 30 cannabis

Do you think your use of cannabis is having a negative effect on your mental or physical health?

- Yes, on both my mental and physical health
- Yes, on my physical health only
- Yes, on my mental health only
- No

Which of the following best describes the frequency you used cannabis using any route of administration in the last 30 days?

- Daily
- Less than daily, but at least once a week
- Less than weekly, but at least once a month

Do you have any current intention to quit using cannabis?

- Yes
- No

Have you used an electronic vaping device to use cannabis in the last 30 days?

- Yes
- No

Current use cannabis

What is your main reason for vaping cannabis now?

- To quit other routes of administration
- To cut down other routes of administration
- To use when I cannot or am not allowed to use via other routes of administration
- To avoid returning to other routes of administration
- Because I enjoy it
- Because it gives me a more pleasurable effect than other routes
- Because they are less harmful than other routes of administration
- Because I was concerned about my health
- Curiosity just wanted to try them
- To save money
- To protect others from second-hand exposure
- For some other reason -please explain

- Don't know
- Prefer not to say

What are any other reasons for vaping cannabis now?

- To quit other routes of administration
- To cut down other routes of administration
- To use when I cannot or am not allowed to use via other routes of administration
- To avoid returning to other routes of administration
- Because I enjoy it
- Because it gives me a more pleasurable effect than other routes
- Because they are less harmful than other routes of administration
- Because I was concerned about my health
- Curiosity just wanted to try them
- To save money
- To protect others from second-hand exposure
- For some other reason -please explain
- Don't know
- Prefer not to say

Which of the following best describes the frequency you vaped cannabis in the last 30 days?

- Daily
- Less than daily, but at least once a week
- Less than weekly, but at least once a month

What is the main electronic vaping device by which you use cannabis?

- Commercially bought electronic or e-cigarette
- Commercially bought electronic device used to vaporise dry herbs
- Commercially bought tabletop vapouriser
- Commercially bought electronic device used to vape liquids (but not an e-cigarette or a tabletop vapouriser)
- Homemade electronic device
- Other, please describe

Are there any other electronic vaping devices by which you use cannabis?

- Commercially bought electronic or e-cigarette
- Commercially bought electronic device used to vaporise dry herbs
- Commercially bought tabletop vapouriser
- Commercially bought electronic device used to vape liquids (but not an e-cigarette or a tabletop vapouriser)
- Homemade electronic device
- Other, please describe

Vaping Synthetic cannabis

You said that you had vaped synthetic cannabis. What type of synthetic cannabis have you vaped?

- Spice
- Black mamba
- Spice gold
- Magic gold
- Exodus
- K2
- Herbal incense
- Spice arctic
- Bonzai
- Annihilation
- Thai high
- AB-FUMINACA
- ADB-PINACA
- AM-2201
- ADB-CHMICA
- JWH-015
- JWH-018
- CP-47-497
- 5F-ADB
- 5F-AKB48
- XLR-11
- UR-144
- HU-210
- MDMB-CHMICA
- Other, please describe

How old were you the first time you ever used synthetic cannabis? (by ANY method not only using an

electronic vaping device)

What was the first route of administration by which you ever used synthetic cannabis?

- Vaping
- Smoking
- Oral (ingesting by mouth)
- Intravenous (injecting into a vein)
- Sniffing/snorting (taking by nose)
- Other, please describe

How old were you when you the first time you ever vaped synthetic cannabis?

What was your main reason for vaping synthetic cannabis the first time?

- To quit other routes of administration
- To cut down other routes of administration
- To use when I could not or was not allowed to use via other routes of administration
- To avoid returning to other routes of administration
- Because I thought I would enjoy it
- Because I though it would give me a more pleasurable effect than other routes
- Because they are less harmful than other routes of administration
- Because I was concerned about my health
- Curiosity just wanted to try them
- To save money
- To protect others from second-hand exposure
- For some other reason -please explain
- Don't know
- Prefer not to say

What were any other reasons for vaping synthetic cannabis the first time?

- To quit other routes of administration

- [ ] To cut down other routes of administration
- [ ] To use when I cannot or am not allowed to use via other routes of administration
- [ ] To avoid returning to other routes of administration
- [ ] Because I enjoy it
- [ ] Because it gives me a more pleasurable effect than other routes
- [ ] Because they are less harmful than other routes of administration
- [ ] Because I was concerned about my health
- [ ] Curiosity just wanted to try them
- [ ] To save money
- [ ] To protect others from second-hand exposure
- [ ] For some other reason -please explain
- [ ] Don't know
- [ ] Prefer not to say

Have you used synthetic cannabis in the last 30 days?

- Yes
- No

Past 30 synthetic cannabis

Do you think your use of synthetic cannabis is having a negative effect on your mental or physical health?

- Yes, on both my mental and physical health
- Yes, on my physical health only
- Yes, on my mental health only
- No

Which of the following best describes the frequency you used synthetic cannabis using any route of administration in the last 30 days?

- Daily
- Less than daily, but at least once a week
- Less than weekly, but at least once a month

Do you have any current intention to quit using synthetic cannabis?

- Yes

No

Have you used an electronic vaping device to use synthetic cannabis in the last 30 days?

- Yes
- No

Current synthetic cannabis use

What is your main reason for vaping synthetic cannabis now?

- To quit other routes of administration
- To cut down other routes of administration
- To use when I cannot or am not allowed to use via other routes of administration
- To avoid returning to other routes of administration
- Because I enjoy it
- Because it gives me a more pleasurable effect than other routes
- Because they are less harmful than other routes of administration
- Because I was concerned about my health
- Curiosity just wanted to try them
- To save money
- To protect others from second-hand exposure
- For some other reason -please explain
- Don't know
- Prefer not to say

What are any other reasons for vaping synthetic cannabis now?

- To quit other routes of administration
- To cut down other routes of administration
- To use when I cannot or am not allowed to use via other routes of administration
- To avoid returning to other routes of administration
- Because I enjoy it
- Because it gives me a more pleasurable effect than other routes
- Because they are less harmful than other routes of administration
- Because I was concerned about my health
- Curiosity just wanted to try them
- To save money

Supplemental material BMJ Publishing Group Limited (BMJ) disclaims all liability and responsibility arising from any reliance placed on this supplemental material which has been supplied by the author(s) BMJ Open

- To protect others from second-hand exposure
- For some other reason -please explain
- Don't know
- Prefer not to say

Which of the following best describes the frequency you vaped synthetic cannabis in the last 30 days?

- Daily
- Less than daily, but at least once a week
- Less than weekly, but at least once a month

What is the main electronic vaping device by which you use synthetic cannabis?

- Commercially bought electronic or e-cigarette
- Commercially bought electronic device used to vaporise dry herbs
- Commercially bought tabletop vapouriser
- Commercially bought electronic device used to vape liquids (but not an e-cigarette or a tabletop vapouriser)
- Homemade electronic device
- Other, please describe

Are there any other electronic vaping devices by which you use synthetic cannabis?

- Commercially bought electronic or e-cigarette
- Commercially bought electronic device used to vaporise dry herbs
- Commercially bought tabletop vapouriser
- Commercially bought electronic device used to vape liquids (but not an e-cigarette or a tabletop vapouriser)
- Homemade electronic device
- Other, please describe

Cocaine vaping

You said that you had vaped cocaine. What type of cocaine have you vaped? (tick all that apply)

- Cocaine powder
- Crack cocaine
- Other, please describe

How old were you the first time you ever used cocaine? (by ANY method not only using an electronic vaping device)

What was the first route of administration by which you ever used cocaine?

- Vaping
- Smoking
- Oral (ingesting by mouth)
- Intravenous (injecting into a vein)
- Sniffing/snorting (taking by nose)
- Other, please describe

How old were you when you the first time you ever vaped cocaine?

What was your main reason for vaping cocaine the first time?

- To quit other routes of administration
- To cut down other routes of administration
- To use when I could not or was not allowed to use via other routes of administration
- To avoid returning to other routes of administration
- Because I thought I would enjoy it
- Because I though it would give me a more pleasurable effect than other routes
- Because they are less harmful than other routes of administration
- Because I was concerned about my health
- Curiosity just wanted to try them
- To save money
- To protect others from second-hand exposure
- For some other reason -please explain
- Don't know
- Prefer not to say

What were any other reasons for vaping cocaine the first time?

- To quit other routes of administration
- To cut down other routes of administration
- To use when I cannot or am not allowed to use via other routes of administration
- To avoid returning to other routes of administration
- Because I enjoy it
- Because it gives me a more pleasurable effect than other routes
- Because they are less harmful than other routes of administration
- Because I was concerned about my health
- Curiosity just wanted to try them
- To save money
- To protect others from second-hand exposure
- For some other reason -please explain
- Don't know
- Prefer not to say

Have you used cocaine in the last 30 days?

- Yes
- No

Past 30 cocaine

Do you think your use of cocaine is having a negative effect on your mental or physical health?

- Yes, on both my mental and physical health
- Yes, on my physical health only
- Yes, on my mental health only
- No

Which of the following best describes the frequency you used cocaine using any route of administration in the last 30 days?

- Daily
- Less than daily, but at least once a week
- Less than weekly, but at least once a month

Do you have any current intention to quit using cocaine?

- Yes
- No

Have you used an electronic vaping device to use cocaine in the last 30 days?

- Yes
- No

Current use cocaine

What is your main reason for vaping cocaine now?

- To quit other routes of administration
- To cut down other routes of administration
- To use when I cannot or am not allowed to use via other routes of administration
- To avoid returning to other routes of administration
- Because I enjoy it
- Because it gives me a more pleasurable effect than other routes
- Because they are less harmful than other routes of administration
- Because I was concerned about my health
- Curiosity just wanted to try them
- To save money
- To protect others from second-hand exposure
- For some other reason -please explain
- Don't know
- Prefer not to say

What are any other reasons for vaping cocaine now?

- To quit other routes of administration
- To cut down other routes of administration
- To use when I cannot or am not allowed to use via other routes of administration
- To avoid returning to other routes of administration
- Because I enjoy it
- Because it gives me a more pleasurable effect than other routes
- Because they are less harmful than other routes of administration
- Because I was concerned about my health
- Curiosity just wanted to try them

- To save money
- To protect others from second-hand exposure
- For some other reason -please explain
- Don't know
- Prefer not to say

Which of the following best describes the frequency you vaped cocaine in the last 30 days?

- Daily
- Less than daily, but at least once a week
- Less than weekly, but at least once a month

What is the main electronic vaping device by which you use cocaine?

- Commercially bought electronic or e-cigarette
- Commercially bought electronic device used to vaporise dry herbs
- Commercially bought tabletop vapouriser
- Commercially bought electronic device used to vape liquids (but not an e-cigarette or a tabletop vapouriser)
- Homemade electronic device
- Other, please describe

Are there any other electronic vaping devices by which you use cocaine?

- Commercially bought electronic or e-cigarette
- Commercially bought electronic device used to vaporise dry herbs
- Commercially bought tabletop vapouriser
- Commercially bought electronic device used to vape liquids (but not an e-cigarette or a tabletop vapouriser)
- Homemade electronic device
- Other, please describe

MDMA Vaping

You said that you had vaped MDMA. What type of MDMA have you vaped? (tick all that apply)

- MDMA (aka Ecstasy Molly, Mandy)
- Other, please describe

How old were you the first time you ever used MDMA? (by ANY method not only using an electronic vaping device)

What was the first route of administration by which you ever used MDMA?

- Vaping
- Smoking
- Oral (ingesting by mouth)
- Intravenous (injecting into a vein)
- Sniffing/snorting (taking by nose)
- Other, please describe

How old were you when you the first time you ever vaped MDMA?

What was your main reason for vaping MDMA the first time?

- To quit other routes of administration
- To cut down other routes of administration
- To use when I could not or was not allowed to use via other routes of administration
- To avoid returning to other routes of administration
- Because I thought I would enjoy it
- Because I though it would give me a more pleasurable effect than other routes
- Because they are less harmful than other routes of administration
- Because I was concerned about my health
- Curiosity just wanted to try them
- To save money
- To protect others from second-hand exposure
- For some other reason -please explain
- Don't know
- Prefer not to say

What were any other reasons for vaping MDMA the first time?

- To quit other routes of administration
- To cut down other routes of administration
- To use when I cannot or am not allowed to use via other routes of administration
- To avoid returning to other routes of administration
- Because I enjoy it
- Because it gives me a more pleasurable effect than other routes
- Because they are less harmful than other routes of administration
- Because I was concerned about my health
- Curiosity just wanted to try them
- To save money
- To protect others from second-hand exposure
- For some other reason -please explain
- Don't know
- Prefer not to say

Have you used MDMA in the last 30 days?

- Yes
- No

Past 30 MDMA

Do you think your use of MDMA is having a negative effect on your mental or physical health?

- Yes, on both my mental and physical health
- Yes, on my physical health only
- Yes, on my mental health only
- No

Which of the following best describes the frequency you used MDMA using any route of administration in the last 30 days?

- Daily
- Less than daily, but at least once a week
- Less than weekly, but at least once a month

Do you have any current intention to quit using MDMA?

- ◯ Yes
- ◯ No

Have you used an electronic vaping device to use MDMA in the last 30 days?

- ◯ Yes
- ◯ No

Current use MDMA

What is your main reason for vaping MDMA now?

- ◯ To quit other routes of administration
- ◯ To cut down other routes of administration
- ◯ To use when I cannot or am not allowed to use via other routes of administration
- ◯ To avoid returning to other routes of administration
- ◯ Because I enjoy it
- ◯ Because it gives me a more pleasurable effect than other routes
- ◯ Because they are less harmful than other routes of administration
- ◯ Because I was concerned about my health
- ◯ Curiosity just wanted to try them
- ◯ To save money
- ◯ To protect others from second-hand exposure
- ◯ For some other reason -please explain ________
- ◯ Don't know
- ◯ Prefer not to say

What are any other reasons for vaping MDMA now?

- ☐ To quit other routes of administration
- ☐ To cut down other routes of administration
- ☐ To use when I cannot or am not allowed to use via other routes of administration
- ☐ To avoid returning to other routes of administration
- ☐ Because I enjoy it
- ☐ Because it gives me a more pleasurable effect than other routes
- ☐ Because they are less harmful than other routes of administration
- ☐ Because I was concerned about my health

- ☐ Curiosity just wanted to try them
- ☐ To save money
- ☐ To protect others from second-hand exposure
- ☐ For some other reason -please explain
- ☐ Don't know
- ☐ Prefer not to say

Which of the following best describes the frequency you vaped MDMA in the last 30 days?

- ○ Daily
- ○ Less than daily, but at least once a week
- ○ Less than weekly, but at least once a month

What is the main electronic vaping device by which you use MDMA?

- ○ Commercially bought electronic or e-cigarette
- ○ Commercially bought electronic device used to vaporise dry herbs
- ○ Commercially bought tabletop vapouriser
- ○ Commercially bought electronic device used to vape liquids (but not an e-cigarette or a tabletop vapouriser)
- ○ Homemade electronic device
- ○ Other, please describe

Are there any other electronic vaping devices by which you use MDMA?

- ☐ Commercially bought electronic or e-cigarette
- ☐ Commercially bought electronic device used to vaporise dry herbs
- ☐ Commercially bought tabletop vapouriser
- ☐ Commercially bought electronic device used to vape liquids (but not an e-cigarette or a tabletop vapouriser)
- ☐ Homemade electronic device
- ☐ Other, please describe

GBL Vaping

You said that you had vaped GBL. What type of GBL have you vaped? (tick all that apply)

- ☐ GBL (aka liquid ecstasy)
- ☐ GHB

- Other, please describe

How old were you the first time you ever used GBL? (by ANY method not only using an electronic vaping device)

What was the first route of administration by which you ever used GBL?

- Vaping
- Smoking
- Oral (ingesting by mouth)
- Intravenous (injecting into a vein)
- Sniffing/snorting (taking by nose)
- Other, please describe

How old were you when you the first time you ever vaped GBL?

What was your main reason for vaping GBL the first time?

- To quit other routes of administration
- To cut down other routes of administration
- To use when I could not or was not allowed to use via other routes of administration
- To avoid returning to other routes of administration
- Because I thought I would enjoy it
- Because I though it would give me a more pleasurable effect than other routes
- Because they are less harmful than other routes of administration
- Because I was concerned about my health
- Curiosity just wanted to try them
- To save money
- To protect others from second-hand exposure
- For some other reason -please explain
- Don't know
- Prefer not to say

What were any other reasons for vaping GBL the first time?

- ☐ To quit other routes of administration
- ☐ To cut down other routes of administration
- ☐ To use when I cannot or am not allowed to use via other routes of administration
- ☐ To avoid returning to other routes of administration
- ☐ Because I enjoy it
- ☐ Because it gives me a more pleasurable effect than other routes
- ☐ Because they are less harmful than other routes of administration
- ☐ Because I was concerned about my health
- ☐ Curiosity just wanted to try them
- ☐ To save money
- ☐ To protect others from second-hand exposure
- ☐ For some other reason -please explain ________
- ☐ Don't know
- ☐ Prefer not to say

Have you used GBL in the last 30 days?

- ○ Yes
- ○ No

Past 30 day GLB

Do you think your use of GLB is having a negative effect on your mental or physical health?

- ○ Yes, on both my mental and physical health
- ○ Yes, on my physical health only
- ○ Yes, on my mental health only
- ○ No

Which of the following best describes the frequency you used GLB using any route of administration in the last 30 days?

- ○ Daily
- ○ Less than daily, but at least once a week
- ○ Less than weekly, but at least once a month

Do you have any current intention to quit using GLB?

- Yes
- No

Have you used an electronic vaping device to use GLB in the last 30 days?

- Yes
- No

Current use GBL

What is your main reason for vaping GBL now?

- To quit other routes of administration
- To cut down other routes of administration
- To use when I cannot or am not allowed to use via other routes of administration
- To avoid returning to other routes of administration
- Because I enjoy it
- Because it gives me a more pleasurable effect than other routes
- Because they are less harmful than other routes of administration
- Because I was concerned about my health
- Curiosity just wanted to try them
- To save money
- To protect others from second-hand exposure
- For some other reason -please explain
- Don't know
- Prefer not to say

What are any other reasons for vaping GBL now?

- To quit other routes of administration
- To cut down other routes of administration
- To use when I cannot or am not allowed to use via other routes of administration
- To avoid returning to other routes of administration
- Because I enjoy it
- Because it gives me a more pleasurable effect than other routes
- Because they are less harmful than other routes of administration

- Because I was concerned about my health
- Curiosity just wanted to try them
- To save money
- To protect others from second-hand exposure
- For some other reason -please explain
- Don't know
- Prefer not to say

Which of the following best describes the frequency you vaped GBL in the last 30 days?

- Daily
- Less than daily, but at least once a week
- Less than weekly, but at least once a month

What is the main electronic vaping device by which you use GBL?

- Commercially bought electronic or e-cigarette
- Commercially bought electronic device used to vaporise dry herbs
- Commercially bought tabletop vapouriser
- Commercially bought electronic device used to vape liquids (but not an e-cigarette or a tabletop vapouriser)
- Homemade electronic device
- Other, please describe

Are there any other electronic vaping devices by which you use GBL?

- Commercially bought electronic or e-cigarette
- Commercially bought electronic device used to vaporise dry herbs
- Commercially bought tabletop vapouriser
- Commercially bought electronic device used to vape liquids (but not an e-cigarette or a tabletop vapouriser)
- Homemade electronic device
- Other, please describe

Vaping Amphetamines

You said that you had vaped amphetamines. What type of amphetamines have you vaped? (tick all that apply)

- Ritalin (Methylphenidate)

- ☐ Crystal methamphetamine (aka Ice, Tina, Crystal)
- ☐ Other, please describe

How old were you the first time you ever used amphetamines? (by ANY method not only using an electronic vaping device)

What was the first route of administration by which you ever used amphetamines?

- ○ Vaping
- ○ Smoking
- ○ Oral (ingesting by mouth)
- ○ Intravenous (injecting into a vein)
- ○ Sniffing/snorting (taking by nose)
- ○ Other, please describe

How old were you when you the first time you ever vaped amphetamines?

What was your main reason for vaping amphetamines the first time?

- ○ To quit other routes of administration
- ○ To cut down other routes of administration
- ○ To use when I could not or was not allowed to use via other routes of administration
- ○ To avoid returning to other routes of administration
- ○ Because I thought I would enjoy it
- ○ Because I though it would give me a more pleasurable effect than other routes
- ○ Because they are less harmful than other routes of administration
- ○ Because I was concerned about my health
- ○ Curiosity just wanted to try them
- ○ To save money
- ○ To protect others from second-hand exposure
- ○ For some other reason -please explain
- ○ Don't know

Prefer not to say

What were any other reasons for vaping amphetamines the first time?

- To quit other routes of administration
- To cut down other routes of administration
- To use when I cannot or am not allowed to use via other routes of administration
- To avoid returning to other routes of administration
- Because I enjoy it
- Because it gives me a more pleasurable effect than other routes
- Because they are less harmful than other routes of administration
- Because I was concerned about my health
- Curiosity just wanted to try them
- To save money
- To protect others from second-hand exposure
- For some other reason -please explain
- Don't know
- Prefer not to say

Have you used amphetamines in the last 30 days?

- Yes
- No

Past 30 amphetamines

Do you think your use of amphetamines is having a negative effect on your mental or physical health?

- Yes, on both my mental and physical health
- Yes, on my physical health only
- Yes, on my mental health only
- No

Which of the following best describes the frequency you used amphetamines using any route of administration in the last 30 days?

- Daily
- Less than daily, but at least once a week

Less than weekly, but at least once a month

Do you have any current intention to quit using amphetamines?

- Yes
- No

Have you used an electronic vaping device to use amphetamines in the last 30 days?

- Yes
- No

Current use amphetamines

What is your main reason for vaping amphetamines now?

- To quit other routes of administration
- To cut down other routes of administration
- To use when I cannot or am not allowed to use via other routes of administration
- To avoid returning to other routes of administration
- Because I enjoy it
- Because it gives me a more pleasurable effect than other routes
- Because they are less harmful than other routes of administration
- Because I was concerned about my health
- Curiosity just wanted to try them
- To save money
- To protect others from second-hand exposure
- For some other reason -please explain
- Don't know
- Prefer not to say

What are any other reasons for vaping amphetamines now?

- To quit other routes of administration
- To cut down other routes of administration
- To use when I cannot or am not allowed to use via other routes of administration
- To avoid returning to other routes of administration
- Because I enjoy it

- ☐ Because it gives me a more pleasurable effect than other routes
- ☐ Because they are less harmful than other routes of administration
- ☐ Because I was concerned about my health
- ☐ Curiosity just wanted to try them
- ☐ To save money
- ☐ To protect others from second-hand exposure
- ☐ For some other reason -please explain
- ☐ Don't know
- ☐ Prefer not to say

Which of the following best describes the frequency you vaped amphetamines in the last 30 days?

- ○ Daily
- ○ Less than daily, but at least once a week
- ○ Less than weekly, but at least once a month

What is the main electronic vaping device by which you use amphetamines?

- ○ Commercially bought electronic or e-cigarette
- ○ Commercially bought electronic device used to vaporise dry herbs
- ○ Commercially bought tabletop vapouriser
- ○ Commercially bought electronic device used to vape liquids (but not an e-cigarette or a tabletop vapouriser)
- ○ Homemade electronic device
- ○ Other, please describe

Are there any other electronic vaping devices by which you use amphetamines?

- ☐ Commercially bought electronic or e-cigarette
- ☐ Commercially bought electronic device used to vaporise dry herbs
- ☐ Commercially bought tabletop vapouriser
- ☐ Commercially bought electronic device used to vape liquids (but not an e-cigarette or a tabletop vapouriser)
- ☐ Homemade electronic device
- ☐ Other, please describe

Vaping Opiods

You said that you had vaped opioids. What type of opioids have you vaped? (tick all that apply)

- Heroin
- Morphine
- Fentanyl
- Other, please describe

How old were you the first time you ever used opioids? (by ANY method not only using an electronic vaping device)

What was the first route of administration by which you ever used opioids?

- Vaping
- Smoking
- Oral (ingesting by mouth)
- Intravenous (injecting into a vein)
- Sniffing/snorting (taking by nose)
- Other, please describe

How old were you when you the first time you ever vaped opioids?

What was your main reason for vaping opioids the first time?

- To quit other routes of administration
- To cut down other routes of administration
- To use when I could not or was not allowed to use via other routes of administration
- To avoid returning to other routes of administration
- Because I thought I would enjoy it
- Because I though it would give me a more pleasurable effect than other routes
- Because they are less harmful than other routes of administration
- Because I was concerned about my health
- Curiosity just wanted to try them
- To save money

- To protect others from second-hand exposure
- For some other reason -please explain
- Don't know
- Prefer not to say

What were any other reasons for vaping opioids the first time?

- To quit other routes of administration
- To cut down other routes of administration
- To use when I cannot or am not allowed to use via other routes of administration
- To avoid returning to other routes of administration
- Because I enjoy it
- Because it gives me a more pleasurable effect than other routes
- Because they are less harmful than other routes of administration
- Because I was concerned about my health
- Curiosity just wanted to try them
- To save money
- To protect others from second-hand exposure
- For some other reason -please explain
- Don't know
- Prefer not to say

Have you used opioids in the last 30 days?

- Yes
- No

Past 30 opioids

Do you think your use of opioids is having a negative effect on your mental or physical health?

- Yes, on both my mental and physical health
- Yes, on my physical health only
- Yes, on my mental health only
- No

Which of the following best describes the frequency you used opioids using any route of administration in

the last 30 days?

- Daily
- Less than daily, but at least once a week
- Less than weekly, but at least once a month

Do you have any current intention to quit using opioids?

- Yes
- No

Have you used an electronic vaping device to use opioids in the last 30 days?

- Yes
- No

Current opioids use

What is your main reason for vaping opioids now?

- To quit other routes of administration
- To cut down other routes of administration
- To use when I cannot or am not allowed to use via other routes of administration
- To avoid returning to other routes of administration
- Because I enjoy it
- Because it gives me a more pleasurable effect than other routes
- Because they are less harmful than other routes of administration
- Because I was concerned about my health
- Curiosity just wanted to try them
- To save money
- To protect others from second-hand exposure
- For some other reason -please explain
- Don't know
- Prefer not to say

What are any other reasons for vaping opioids now?

- To quit other routes of administration
- To cut down other routes of administration

- ☐ To use when I cannot or am not allowed to use via other routes of administration
- ☐ To avoid returning to other routes of administration
- ☐ Because I enjoy it
- ☐ Because it gives me a more pleasurable effect than other routes
- ☐ Because they are less harmful than other routes of administration
- ☐ Because I was concerned about my health
- ☐ Curiosity just wanted to try them
- ☐ To save money
- ☐ To protect others from second-hand exposure
- ☐ For some other reason -please explain
- ☐ Don't know
- ☐ Prefer not to say

Which of the following best describes the frequency you vaped opioids in the last 30 days?

- ○ Daily
- ○ Less than daily, but at least once a week
- ○ Less than weekly, but at least once a month

What is the main electronic vaping device by which you use opioids?

- ○ Commercially bought electronic or e-cigarette
- ○ Commercially bought electronic device used to vaporise dry herbs
- ○ Commercially bought tabletop vapouriser
- ○ Commercially bought electronic device used to vape liquids (but not an e-cigarette or a tabletop vapouriser)
- ○ Homemade electronic device
- ○ Other, please describe

Are there any other electronic vaping devices by which you use opioids?

- ☐ Commercially bought electronic or e-cigarette
- ☐ Commercially bought electronic device used to vaporise dry herbs
- ☐ Commercially bought tabletop vapouriser
- ☐ Commercially bought electronic device used to vape liquids (but not an e-cigarette or a tabletop vapouriser)
- ☐ Homemade electronic device
- ☐ Other, please describe

Roberts E, *et al. BMJ Open* 2023; 13:e066826. doi: 10.1136/bmjopen-2022-066826

Vaping ketamine

You said that you had vaped ketamine. What type of ketamine have you vaped? (tick all that apply)

- ☐ Ketamine (aka Special-K, Ket)
- ☐ Other, please describe

How old were you the first time you ever used ketamine? (by ANY method not only using an electronic vaping device)

What was the first route of administration by which you ever used ketamine?

- ○ Vaping
- ○ Smoking
- ○ Oral (ingesting by mouth)
- ○ Intravenous (injecting into a vein)
- ○ Sniffing/snorting (taking by nose)
- ○ Other, please describe

How old were you when you the first time you ever vaped ketamine?

What was your main reason for vaping ketamine the first time?

- ○ To quit other routes of administration
- ○ To cut down other routes of administration
- ○ To use when I could not or was not allowed to use via other routes of administration
- ○ To avoid returning to other routes of administration
- ○ Because I thought I would enjoy it
- ○ Because I though it would give me a more pleasurable effect than other routes
- ○ Because they are less harmful than other routes of administration
- ○ Because I was concerned about my health
- ○ Curiosity just wanted to try them
- ○ To save money

- To protect others from second-hand exposure
- For some other reason -please explain
- Don't know
- Prefer not to say

What were any other reasons for vaping ketamine the first time?

- To quit other routes of administration
- To cut down other routes of administration
- To use when I cannot or am not allowed to use via other routes of administration
- To avoid returning to other routes of administration
- Because I enjoy it
- Because it gives me a more pleasurable effect than other routes
- Because they are less harmful than other routes of administration
- Because I was concerned about my health
- Curiosity just wanted to try them
- To save money
- To protect others from second-hand exposure
- For some other reason -please explain
- Don't know
- Prefer not to say

Have you used ketamine in the last 30 days?

- Yes
- No

Past 30 days ketamine

Do you think your use of ketamine is having a negative effect on your mental or physical health?

- Yes, on both my mental and physical health
- Yes, on my physical health only
- Yes, on my mental health only
- No

Which of the following best describes the frequency you used ketamine using any route of administration in the last 30 days?

- Daily
- Less than daily, but at least once a week
- Less than weekly, but at least once a month

Do you have any current intention to quit using ketamine?

- Yes
- No

Have you used an electronic vaping device to use ketamine in the last 30 days?

- Yes
- No

Current use ketamine

What is your main reason for vaping ketamine now?

- To quit other routes of administration
- To cut down other routes of administration
- To use when I cannot or am not allowed to use via other routes of administration
- To avoid returning to other routes of administration
- Because I enjoy it
- Because it gives me a more pleasurable effect than other routes
- Because they are less harmful than other routes of administration
- Because I was concerned about my health
- Curiosity just wanted to try them
- To save money
- To protect others from second-hand exposure
- For some other reason -please explain
- Don't know
- Prefer not to say

What are any other reasons for vaping ketamine now?

- To quit other routes of administration
- ☐ To cut down other routes of administration
- ☐ To use when I cannot or am not allowed to use via other routes of administration
- ☐ To avoid returning to other routes of administration
- ☐ Because I enjoy it
- ☐ Because it gives me a more pleasurable effect than other routes
- ☐ Because they are less harmful than other routes of administration
- ☐ Because I was concerned about my health
- ☐ Curiosity just wanted to try them
- ☐ J. To save money
- ☐ To protect others from second-hand exposure
- ☐ For some other reason -please explain
- ☐ Don't know
- ☐ Prefer not to say

Which of the following best describes the frequency you vaped ketamine in the last 30 days?

- ○ Daily
- ○ Less than daily, but at least once a week
- ○ Less than weekly, but at least once a month

What is the main electronic vaping device by which you use ketamine?

- ○ Commercially bought electronic or e-cigarette
- ○ Commercially bought electronic device used to vaporise dry herbs
- ○ Commercially bought tabletop vapouriser
- ○ Commercially bought electronic device used to vape liquids (but not an e-cigarette or a tabletop vapouriser)
- ○ Homemade electronic device
- ○ Other, please describe

Are there any other electronic vaping devices by which you use ketamine?

- ☐ Commercially bought electronic or e-cigarette
- ☐ Commercially bought electronic device used to vaporise dry herbs
- ☐ Commercially bought tabletop vapouriser
- ☐ Commercially bought electronic device used to vape liquids (but not an e-cigarette or a tabletop vapouriser)
- ☐ Homemade electronic device
- ☐

Supplemental material BMJ Publishing Group Limited (BMJ) disclaims all liability and responsibility arising from any reliance placed on this supplemental material which has been supplied by the author(s) BMJ Open

Other, please describe

Vaping Mephedrone

You said that you had vaped mephedrone. What type of mephedrone have you vaped? (tick all that apply)

- Mephedrone (aka meow-meow, M-CAT)
- Other, please describe

How old were you the first time you ever used mephedrone? (by ANY method not only using an electronic vaping device)

What was the first route of administration by which you ever used mephedrone?

- Vaping
- Smoking
- Oral (ingesting by mouth)
- Intravenous (injecting into a vein)
- Sniffing/snorting (taking by nose)
- Other, please describe

How old were you when you the first time you ever vaped mephedrone?

What was your main reason for vaping mephedrone the first time?

- To quit other routes of administration
- To cut down other routes of administration
- To use when I could not or was not allowed to use via other routes of administration
- To avoid returning to other routes of administration
- Because I thought I would enjoy it
- Because I though it would give me a more pleasurable effect than other routes
- Because they are less harmful than other routes of administration

Roberts E, *et al. BMJ Open* 2023; 13:e066826. doi: 10.1136/bmjopen-2022-066826

- Because I was concerned about my health
- Curiosity just wanted to try them
- To save money
- To protect others from second-hand exposure
- For some other reason -please explain
- Don't know
- Prefer not to say

What were any other reasons for vaping mephedrone the first time?

- To quit other routes of administration
- To cut down other routes of administration
- To use when I cannot or am not allowed to use via other routes of administration
- To avoid returning to other routes of administration
- Because I enjoy it
- Because it gives me a more pleasurable effect than other routes
- Because they are less harmful than other routes of administration
- Because I was concerned about my health
- Curiosity just wanted to try them
- To save money
- To protect others from second-hand exposure
- For some other reason -please explain
- Don't know
- Prefer not to say

Have you used mephedrone in the last 30 days?

- Yes
- No

Past 30 mephedrone

Do you think your use of mephedrone is having a negative effect on your mental or physical health?

- Yes, on both my mental and physical health
- Yes, on my physical health only
- Yes, on my mental health only
- No

Which of the following best describes the frequency you used mephedrone using any route of administration in the last 30 days?

- Daily
- Less than daily, but at least once a week
- Less than weekly, but at least once a month

Do you have any current intention to quit using mephedrone?

- Yes
- No

Have you used an electronic vaping device to use mephedrone in the last 30 days?

- Yes
- No

Current use mephedrone

What is your main reason for vaping mephedrone now?

- To quit other routes of administration
- To cut down other routes of administration
- To use when I cannot or am not allowed to use via other routes of administration
- To avoid returning to other routes of administration
- Because I enjoy it
- Because it gives me a more pleasurable effect than other routes
- Because they are less harmful than other routes of administration
- Because I was concerned about my health
- Curiosity just wanted to try them
- To save money
- To protect others from second-hand exposure
- For some other reason -please explain
- Don't know
- Prefer not to say

What are any other reasons for vaping mephedrone now?

- To quit other routes of administration
- To cut down other routes of administration
- To use when I cannot or am not allowed to use via other routes of administration
- To avoid returning to other routes of administration
- Because I enjoy it
- Because it gives me a more pleasurable effect than other routes
- Because they are less harmful than other routes of administration
- Because I was concerned about my health
- Curiosity just wanted to try them
- To save money
- To protect others from second-hand exposure
- For some other reason -please explain
- Don't know
- Prefer not to say

Which of the following best describes the frequency you vaped mephedrone in the last 30 days?

- Daily
- Less than daily, but at least once a week
- Less than weekly, but at least once a month

What is the main electronic vaping device by which you use mephedrone?

- Commercially bought electronic or e-cigarette
- Commercially bought electronic device used to vaporise dry herbs
- Commercially bought tabletop vapouriser
- Commercially bought electronic device used to vape liquids (but not an e-cigarette or a tabletop vapouriser)
- Homemade electronic device
- Other, please describe

Are there any other electronic vaping devices by which you use mephedrone?

- Commercially bought electronic or e-cigarette
- Commercially bought electronic device used to vaporise dry herbs
- Commercially bought tabletop vapouriser

- [ ] Commercially bought electronic device used to vape liquids (but not an e-cigarette or a tabletop vapouriser)
- [ ] Homemade electronic device
- [ ] Other, please describe

Vaping DMT

You said that you had vaped DMT. What type of DMT have you vaped? (tick all that apply)

- [ ] DMT
- [ ] Other, please describe

How old were you the first time you ever used DMT? (by ANY method not only using an electronic vaping device)

What was the first route of administration by which you ever used DMT?

- ○ Vaping
- ○ Smoking
- ○ Oral (ingesting by mouth)
- ○ Intravenous (injecting into a vein)
- ○ Sniffing/snorting (taking by nose)
- ○ Other, please describe

How old were you when you the first time you ever vaped DMT?

What was your main reason for vaping DMT the first time?

- ○ To quit other routes of administration
- ○ To cut down other routes of administration
- ○ To use when I could not or was not allowed to use via other routes of administration
- ○ To avoid returning to other routes of administration
- ○ Because I thought I would enjoy it

- Because I though it would give me a more pleasurable effect than other routes
- Because they are less harmful than other routes of administration
- Because I was concerned about my health
- Curiosity just wanted to try them
- To save money
- To protect others from second-hand exposure
- For some other reason -please explain
- Don't know
- Prefer not to say

What were any other reasons for vaping DMT the first time?

- To quit other routes of administration
- To cut down other routes of administration
- To use when I cannot or am not allowed to use via other routes of administration
- To avoid returning to other routes of administration
- Because I enjoy it
- Because it gives me a more pleasurable effect than other routes
- Because they are less harmful than other routes of administration
- Because I was concerned about my health
- Curiosity just wanted to try them
- To save money
- To protect others from second-hand exposure
- For some other reason -please explain
- Don't know
- Prefer not to say

Have you used DMT in the last 30 days?

- Yes
- No

Past 30 DMT

Do you think your use of DMT is having a negative effect on your mental or physical health?

- Yes, on both my mental and physical health
- Yes, on my physical health only

- Yes, on my mental health only
- No

Which of the following best describes the frequency you used DMT using any route of administration in the last 30 days?

- Daily
- Less than daily, but at least once a week
- Less than weekly, but at least once a month

Do you have any current intention to quit using DMT?

- Yes
- No

Have you used an electronic vaping device to use DMT in the last 30 days?

- Yes
- No

Current Use DMT

What is your main reason for vaping DMT now?

- To quit other routes of administration
- To cut down other routes of administration
- To use when I cannot or am not allowed to use via other routes of administration
- To avoid returning to other routes of administration
- Because I enjoy it
- Because it gives me a more pleasurable effect than other routes
- Because they are less harmful than other routes of administration
- Because I was concerned about my health
- Curiosity just wanted to try them
- To save money
- To protect others from second-hand exposure
- For some other reason -please explain
- Don't know
- Prefer not to say

What are any other reasons for vaping DMT now?

- To quit other routes of administration
- To cut down other routes of administration
- To use when I cannot or am not allowed to use via other routes of administration
- To avoid returning to other routes of administration
- Because I enjoy it
- Because it gives me a more pleasurable effect than other routes
- Because they are less harmful than other routes of administration
- Because I was concerned about my health
- Curiosity just wanted to try them
- J. To save money
- To protect others from second-hand exposure
- For some other reason -please explain
- Don't know
- Prefer not to say

Which of the following best describes the frequency you vaped DMT in the last 30 days?

- Daily
- Less than daily, but at least once a week
- Less than weekly, but at least once a month

What is the main electronic vaping device by which you use DMT?

- Commercially bought electronic or e-cigarette
- Commercially bought electronic device used to vaporise dry herbs
- Commercially bought tabletop vapouriser
- Commercially bought electronic device used to vape liquids (but not an e-cigarette or a tabletop vapouriser)
- Homemade electronic device
- Other, please describe

Are there any other electronic vaping devices by which you use DMT?

- Commercially bought electronic or e-cigarette
- Commercially bought electronic device used to vaporise dry herbs

Commercially bought tabletop vapouriser

- Commercially bought electronic device used to vape liquids (but not an e-cigarette or a tabletop vapouriser)
- Homemade electronic device
- Other, please describe

Vaping Psilocybin

You said that you had vaped psilocybin. What type of psilocybin have you vaped? (tick all that apply)

- Psilocybin
- Other, please describe

How old were you the first time you ever used psilocybin? (by ANY method not only using an electronic vaping device)

What was the first route of administration by which you ever used psilocybin?

- Vaping
- Smoking
- Oral (ingesting by mouth)
- Intravenous (injecting into a vein)
- Sniffing/snorting (taking by nose)
- Other, please describe

How old were you when you the first time you ever vaped psilocybin?

What was your main reason for vaping psilocybin the first time?

- To quit other routes of administration
- To cut down other routes of administration
- To use when I cannot or am not allowed to use via other routes of administration
- To avoid returning to other routes of administration

- Because I enjoy it
- Because it gives me a more pleasurable effect than other routes
- Because they are less harmful than other routes of administration
- Because I was concerned about my health
- Curiosity just wanted to try them
- To save money
- To protect others from second-hand exposure
- For some other reason -please explain
- Don't know
- Prefer not to say

What were any other reasons for vaping psilocybin the first time?

- To quit other routes of administration
- To cut down other routes of administration
- To use when I cannot or am not allowed to use via other routes of administration
- To avoid returning to other routes of administration
- Because I enjoy it
- Because it gives me a more pleasurable effect than other routes
- Because they are less harmful than other routes of administration
- Because I was concerned about my health
- Curiosity just wanted to try them
- To save money
- To protect others from second-hand exposure
- For some other reason -please explain
- Don't know
- Prefer not to say

Have you used psilocybin in the last 30 days?

- Yes
- No

Past 30 psilocybin

Do you think your use of psilocybin is having a negative effect on your mental or physical health?

- Yes, on both my mental and physical health

- Yes, on my physical health only
- Yes, on my mental health only
- No

Which of the following best describes the frequency you used psilocybin using any route of administration in the last 30 days?

- Daily
- Less than daily, but at least once a week
- Less than weekly, but at least once a month

Do you have any current intention to quit using psilocybin?

- Yes
- No

Have you used an electronic vaping device to use psilocybin in the last 30 days?

- Yes
- No

Current use psilocybin

What is your main reason for vaping psilocybin now?

- To quit other routes of administration
- To cut down other routes of administration
- To use when I cannot or am not allowed to use via other routes of administration
- To avoid returning to other routes of administration
- Because I enjoy it
- Because it gives me a more pleasurable effect than other routes
- Because they are less harmful than other routes of administration
- Because I was concerned about my health
- Curiosity just wanted to try them
- To save money
- To protect others from second-hand exposure
- For some other reason -please explain
- Don't know

Prefer not to say

What are any other reasons for vaping psilocybin now?

- To quit other routes of administration
- To cut down other routes of administration
- To use when I cannot or am not allowed to use via other routes of administration
- To avoid returning to other routes of administration
- Because I enjoy it
- Because it gives me a more pleasurable effect than other routes
- Because they are less harmful than other routes of administration
- Because I was concerned about my health
- Curiosity just wanted to try them
- J. To save money
- To protect others from second-hand exposure
- For some other reason -please explain
- Don't know
- Prefer not to say

Which of the following best describes the frequency you vaped psilocybin in the last 30 days?

- Daily
- Less than daily, but at least once a week
- Less than weekly, but at least once a month

What is the main electronic vaping device by which you use psilocybin?

- Commercially bought electronic or e-cigarette
- Commercially bought electronic device used to vaporise dry herbs
- Commercially bought tabletop vapouriser
- Commercially bought electronic device used to vape liquids (but not an e-cigarette or a tabletop vapouriser)
- Homemade electronic device
- Other, please describe

Are there any other electronic vaping devices by which you use psilocybin?

- Commercially bought electronic or e-cigarette

- ☐ Commercially bought electronic device used to vaporise dry herbs
- ☐ Commercially bought tabletop vapouriser
- ☐ Commercially bought electronic device used to vape liquids (but not an e-cigarette or a tabletop vapouriser)
- ☐ Homemade electronic device
- ☐ Other, please describe

Vaping Benzodiazepines

You said that you had vaped benzodiazepines. What type of benzodiazepines have you vaped? (tick all that apply)

- ☐ Diazepam (aka blues, Valium)
- ☐ Clonazepam (aka Rivotril)
- ☐ Alprazolam (aka Xanax)
- ☐ Other, please describe

How old were you the first time you ever used benzodiazepines? (by ANY method not only using an electronic vaping device)

What was the first route of administration by which you ever used benzodiazepines?

- ○ Vaping
- ○ Smoking
- ○ Oral (ingesting by mouth)
- ○ Intravenous (injecting into a vein)
- ○ Sniffing/snorting (taking by nose)
- ○ Other, please describe

How old were you when you the first time you ever vaped benzodiazepines?

What was your main reason for vaping benzodiazepines the first time?

- ○ To quit other routes of administration

- To cut down other routes of administration
- To use when I could not or was not allowed to use via other routes of administration
- To avoid returning to other routes of administration
- Because I thought I would enjoy it
- Because I though it would give me a more pleasurable effect than other routes
- Because they are less harmful than other routes of administration
- Because I was concerned about my health
- Curiosity just wanted to try them
- To save money
- To protect others from second-hand exposure
- For some other reason -please explain
- Don't know
- Prefer not to say

What were any other reasons for vaping benzodiazepines the first time?

- To quit other routes of administration
- To cut down other routes of administration
- To use when I cannot or am not allowed to use via other routes of administration
- To avoid returning to other routes of administration
- Because I enjoy it
- Because it gives me a more pleasurable effect than other routes
- Because they are less harmful than other routes of administration
- Because I was concerned about my health
- Curiosity just wanted to try them
- To save money
- To protect others from second-hand exposure
- For some other reason -please explain
- Don't know
- Prefer not to say

Have you used benzodiazepines in the last 30 days?

- Yes
- No

Past 30 benzodiazepines

Do you think your use of benzodiazepines is having a negative effect on your mental or physical health?

- Yes, on both my mental and physical health
- Yes, on my physical health only
- Yes, on my mental health only
- No

Which of the following best describes the frequency you used benzodiazepines using any route of administration in the last 30 days?

- Daily
- Less than daily, but at least once a week
- Less than weekly, but at least once a month

Do you have any current intention to quit using benzodiazepines?

- Yes
- No

Have you used an electronic vaping device to use benzodiazepines in the last 30 days?

- Yes
- No

Current use benzodiazepines

What is your main reason for vaping benzodiazepines now?

- To quit other routes of administration
- To cut down other routes of administration
- To use when I cannot or am not allowed to use via other routes of administration
- To avoid returning to other routes of administration
- Because I enjoy it
- Because it gives me a more pleasurable effect than other routes
- Because they are less harmful than other routes of administration
- Because I was concerned about my health
- Curiosity just wanted to try them
- To save money
- To protect others from second-hand exposure

- For some other reason -please explain
- Don't know
- Prefer not to say

What are any other reasons for vaping benzodiazepines now?

- To quit other routes of administration
- To cut down other routes of administration
- To use when I cannot or am not allowed to use via other routes of administration
- To avoid returning to other routes of administration
- Because I enjoy it
- Because it gives me a more pleasurable effect than other routes
- Because they are less harmful than other routes of administration
- Because I was concerned about my health
- Curiosity just wanted to try them
- To save money
- To protect others from second-hand exposure
- For some other reason -please explain
- Don't know
- Prefer not to say

Which of the following best describes the frequency you vaped benzodiazepines in the last 30 days?

- Daily
- Less than daily, but at least once a week
- Less than weekly, but at least once a month

What is the main electronic vaping device by which you use benzodiazepines?

- Commercially bought electronic or e-cigarette
- Commercially bought electronic device used to vaporise dry herbs
- Commercially bought tabletop vapouriser
- Commercially bought electronic device used to vape liquids (but not an e-cigarette or a tabletop vapouriser)
- Homemade electronic device
- Other, please describe

Are there any other electronic vaping devices by which you use benzodiazepines?

- ☐ Commercially bought electronic or e-cigarette
- ☐ Commercially bought electronic device used to vaporise dry herbs
- ☐ Commercially bought tabletop vapouriser
- ☐ Commercially bought electronic device used to vape liquids (but not an e-cigarette or a tabletop vapouriser)
- ☐ Homemade electronic device
- ☐ Other, please describe

Vaping 2C

You said that you had vaped 2C. What type of 2C have you vaped? (tick all that apply)

- ☐ 2CB
- ☐ 2CE
- ☐ Other, please describe

How old were you the first time you ever used 2C? (by ANY method not only using an electronic vaping device)

What was the first route of administration by which you ever used 2C?

- ○ Vaping
- ○ Smoking
- ○ Oral (ingesting by mouth)
- ○ Intravenous (injecting into a vein)
- ○ Sniffing/snorting (taking by nose)
- ○ Other, please describe

How old were you when you the first time you ever vaped 2C?

What was your main reason for vaping 2C the first time?

- To quit other routes of administration
- To cut down other routes of administration
- To use when I could not or was not allowed to use via other routes of administration
- To avoid returning to other routes of administration
- Because I thought I would enjoy it
- Because I though it would give me a more pleasurable effect than other routes
- Because they are less harmful than other routes of administration
- Because I was concerned about my health
- Curiosity just wanted to try them
- To save money
- To protect others from second-hand exposure
- For some other reason -please explain
- Don't know
- Prefer not to say

What were any other reasons for vaping 2C the first time?

- To quit other routes of administration
- To cut down other routes of administration
- To use when I cannot or am not allowed to use via other routes of administration
- To avoid returning to other routes of administration
- Because I enjoy it
- Because it gives me a more pleasurable effect than other routes
- Because they are less harmful than other routes of administration
- Because I was concerned about my health
- Curiosity just wanted to try them
- To save money
- To protect others from second-hand exposure
- For some other reason -please explain
- Don't know
- Prefer not to say

Have you used 2C in the last 30 days?

- Yes
- No

Past 30 2C

Do you think your use of 2C is having a negative effect on your mental or physical health?

- Yes, on both my mental and physical health
- Yes, on my physical health only
- Yes, on my mental health only
- No

Which of the following best describes the frequency you used 2C using any route of administration in the last 30 days?

- Daily
- Less than daily, but at least once a week
- Less than weekly, but at least once a month

Do you have any current intention to quit using 2C?

- Yes
- No

Have you used an electronic vaping device to use 2C in the last 30 days?

- Yes
- No

Current use 2C

What is your main reason for vaping 2C now?

- To quit other routes of administration
- To cut down other routes of administration
- To use when I cannot or am not allowed to use via other routes of administration
- To avoid returning to other routes of administration
- Because I enjoy it
- Because it gives me a more pleasurable effect than other routes
- Because they are less harmful than other routes of administration
- Because I was concerned about my health
- Curiosity just wanted to try them
- To save money

- To protect others from second-hand exposure
- For some other reason -please explain
- Don't know
- Prefer not to say

What are any other reasons for vaping 2C now?

- To quit other routes of administration
- To cut down other routes of administration
- To use when I cannot or am not allowed to use via other routes of administration
- To avoid returning to other routes of administration
- Because I enjoy it
- Because it gives me a more pleasurable effect than other routes
- Because they are less harmful than other routes of administration
- Because I was concerned about my health
- Curiosity just wanted to try them
- J. To save money
- To protect others from second-hand exposure
- For some other reason -please explain
- Don't know
- Prefer not to say

Which of the following best describes the frequency you vaped 2C in the last 30 days?

- Daily
- Less than daily, but at least once a week
- Less than weekly, but at least once a month

What is the main electronic vaping device by which you use 2C?

- Commercially bought electronic or e-cigarette
- Commercially bought electronic device used to vaporise dry herbs
- Commercially bought tabletop vapouriser
- Commercially bought electronic device used to vape liquids (but not an e-cigarette or a tabletop vapouriser)
- Homemade electronic device
- Other, please describe

Are there any other electronic vaping devices by which you use 2C?

- ☐ Commercially bought electronic or e-cigarette
- ☐ Commercially bought electronic device used to vaporise dry herbs
- ☐ Commercially bought tabletop vapouriser
- ☐ Commercially bought electronic device used to vape liquids (but not an e-cigarette or a tabletop vapouriser)
- ☐ Homemade electronic device
- ☐ Other, please describe

Vaping alpha-PVP

You said that you had vaped alpha-PVP. What type of alpha-PVP have you vaped? (tick all that apply)

- ☐ alpha-PVP (aka gravel, flakka)
- ☐ Other, please describe

How old were you the first time you ever used alpha-PVP? (by ANY method not only using an electronic vaping device)

What was the first route of administration by which you ever used alpha-PVP?

- ○ Vaping
- ○ Smoking
- ○ Oral (ingesting by mouth)
- ○ Intravenous (injecting into a vein)
- ○ Sniffing/snorting (taking by nose)
- ○ Other, please describe

How old were you when you the first time you ever vaped alpha-PVP?

What was your main reason for vaping alpha-PVP the first time?

- To quit other routes of administration
- To cut down other routes of administration
- To use when I could not or was not allowed to use via other routes of administration
- To avoid returning to other routes of administration
- Because I thought I would enjoy it
- Because I though it would give me a more pleasurable effect than other routes
- Because they are less harmful than other routes of administration
- Because I was concerned about my health
- Curiosity just wanted to try them
- To save money
- To protect others from second-hand exposure
- For some other reason -please explain
- Don't know
- Prefer not to say

What were any other reasons for vaping alpha-PVP the first time?

- To quit other routes of administration
- To cut down other routes of administration
- To use when I cannot or am not allowed to use via other routes of administration
- To avoid returning to other routes of administration
- Because I enjoy it
- Because it gives me a more pleasurable effect than other routes
- Because they are less harmful than other routes of administration
- Because I was concerned about my health
- Curiosity just wanted to try them
- To save money
- To protect others from second-hand exposure
- For some other reason -please explain
- Don't know
- Prefer not to say

Have you used alpha-PVP in the last 30 days?

- Yes
- No

Past 30 alpha-PVP

Do you think your use of alpha-PVP is having a negative effect on your mental or physical health?

- Yes, on both my mental and physical health
- Yes, on my physical health only
- Yes, on my mental health only
- No

Which of the following best describes the frequency you used alpha-PVP using any route of administration in the last 30 days?

- Daily
- Less than daily, but at least once a week
- Less than weekly, but at least once a month

Do you have any current intention to quit using alpha-PVP?

- Yes
- No

Have you used an electronic vaping device to use alpha-PVP in the last 30 days?

- Yes
- No

Current use alpha-PVP

What is your main reason for vaping alpha-PVP now?

- To quit other routes of administration
- To cut down other routes of administration
- To use when I cannot or am not allowed to use via other routes of administration
- To avoid returning to other routes of administration
- Because I enjoy it
- Because it gives me a more pleasurable effect than other routes
- Because they are less harmful than other routes of administration
- Because I was concerned about my health
- Curiosity just wanted to try them
- To save money

- To protect others from second-hand exposure
- For some other reason -please explain
- Don't know
- Prefer not to say

What are any other reasons for vaping alpha-PVP now?

- To quit other routes of administration
- To cut down other routes of administration
- To use when I cannot or am not allowed to use via other routes of administration
- To avoid returning to other routes of administration
- Because I enjoy it
- Because it gives me a more pleasurable effect than other routes
- Because they are less harmful than other routes of administration
- Because I was concerned about my health
- Curiosity just wanted to try them
- To save money
- To protect others from second-hand exposure
- For some other reason -please explain
- Don't know
- Prefer not to say

Which of the following best describes the frequency you vaped alpha-PVP in the last 30 days?

- Daily
- Less than daily, but at least once a week
- Less than weekly, but at least once a month

What is the main electronic vaping device by which you use alpha-PVP?

- Commercially bought electronic or e-cigarette
- Commercially bought electronic device used to vaporise dry herbs
- Commercially bought tabletop vapouriser
- Commercially bought electronic device used to vape liquids (but not an e-cigarette or a tabletop vapouriser)
- Homemade electronic device
- Other, please describe

Are there any other electronic vaping devices by which you use alpha-PVP?

- Commercially bought electronic or e-cigarette
- Commercially bought electronic device used to vaporise dry herbs
- Commercially bought tabletop vapouriser
- Commercially bought electronic device used to vape liquids (but not an e-cigarette or a tabletop vapouriser)
- Homemade electronic device
- Other, please describe

Vaping NBOMe

You said that you had vaped NBOMe What type of NBOMe have you vaped? (tick all that apply)

- NBOMe (aka N-Bomb)
- Other, please describe

How old were you the first time you ever used NBOMe? (by ANY method not only using an electronic vaping device)

What was the first route of administration by which you ever used NBOMe?

- Vaping
- Smoking
- Oral (ingesting by mouth)
- Intravenous (injecting into a vein)
- Sniffing/snorting (taking by nose)
- Other, please describe

How old were you when you the first time you ever vaped NBOMe?

What was your main reason for vaping NBOMe the first time?

- To quit other routes of administration
- To cut down other routes of administration
- To use when I could not or was not allowed to use via other routes of administration
- To avoid returning to other routes of administration
- Because I thought I would enjoy it
- Because I though it would give me a more pleasurable effect than other routes
- Because they are less harmful than other routes of administration
- Because I was concerned about my health
- Curiosity just wanted to try them
- To save money
- To protect others from second-hand exposure
- For some other reason -please explain
- Don't know
- Prefer not to say

What were any other reasons for vaping NBOMe the first time?

- To quit other routes of administration
- To cut down other routes of administration
- To use when I cannot or am not allowed to use via other routes of administration
- To avoid returning to other routes of administration
- Because I enjoy it
- Because it gives me a more pleasurable effect than other routes
- Because they are less harmful than other routes of administration
- Because I was concerned about my health
- Curiosity just wanted to try them
- To save money
- To protect others from second-hand exposure
- For some other reason -please explain
- Don't know
- Prefer not to say

Have you used NBOMe in the last 30 days?

- Yes
- No

Past 30 NBOMe

Do you think your use of NBOMe is having a negative effect on your mental or physical health?

- Yes, on both my mental and physical health
- Yes, on my physical health only
- Yes, on my mental health only
- No

Which of the following best describes the frequency you used NBOMe using any route of administration in the last 30 days?

- Daily
- Less than daily, but at least once a week
- Less than weekly, but at least once a month

Do you have any current intention to quit using NBOMe?

- Yes
- No

Have you used an electronic vaping device to use NBOMe in the last 30 days?

- Yes
- No

Current use NBOMe

What is your main reason for vaping NBOMe now?

- To quit other routes of administration
- To cut down other routes of administration
- To use when I cannot or am not allowed to use via other routes of administration
- To avoid returning to other routes of administration
- Because I enjoy it
- Because it gives me a more pleasurable effect than other routes
- Because they are less harmful than other routes of administration
- Because I was concerned about my health
- Curiosity just wanted to try them
- To save money

- To protect others from second-hand exposure
- For some other reason -please explain
- Don't know
- Prefer not to say

What are any other reasons for vaping NBOMe now?

- To quit other routes of administration
- To cut down other routes of administration
- To use when I cannot or am not allowed to use via other routes of administration
- To avoid returning to other routes of administration
- Because I enjoy it
- Because it gives me a more pleasurable effect than other routes
- Because they are less harmful than other routes of administration
- Because I was concerned about my health
- Curiosity just wanted to try them
- To save money
- To protect others from second-hand exposure
- For some other reason -please explain
- Don't know
- Prefer not to say

Which of the following best describes the frequency you vaped NBOMe in the last 30 days?

- Daily
- Less than daily, but at least once a week
- Less than weekly, but at least once a month

What is the main electronic vaping device by which you use NBOMe?

- Commercially bought electronic or e-cigarette
- Commercially bought electronic device used to vaporise dry herbs
- Commercially bought tabletop vapouriser
- Commercially bought electronic device used to vape liquids (but not an e-cigarette or a tabletop vapouriser)
- Homemade electronic device
- Other, please describe

Are there any other electronic vaping devices by which you use NBOMe?

- ☐ Commercially bought electronic or e-cigarette
- ☐ Commercially bought electronic device used to vaporise dry herbs
- ☐ Commercially bought tabletop vapouriser
- ☐ Commercially bought electronic device used to vape liquids (but not an e-cigarette or a tabletop vapouriser)
- ☐ Homemade electronic device
- ☐ Other, please describe

Vaping Other

You said that you had vaped other substances. What type of other substances have you vaped? (tick all that apply)

How old were you the first time you ever used these other substances? (by ANY method not only using an electronic vaping device)

What was the first route of administration by which you ever used these other substances?

- ○ Vaping
- ○ Smoking
- ○ Oral (ingesting by mouth)
- ○ Intravenous (injecting into a vein)
- ○ Sniffing/snorting (taking by nose)
- ○ Other, please describe

How old were you when you the first time you ever vaped these other substances?

What was your main reason for vaping these other substances the first time?

- To quit other routes of administration
- To cut down other routes of administration
- To use when I could not or was not allowed to use via other routes of administration
- To avoid returning to other routes of administration
- Because I thought I would enjoy it
- Because I though it would give me a more pleasurable effect than other routes
- Because they are less harmful than other routes of administration
- Because I was concerned about my health
- Curiosity just wanted to try them
- To save money
- To protect others from second-hand exposure
- For some other reason -please explain
- Don't know
- Prefer not to say

What were any other reasons for vaping these other substances the first time?

- To quit other routes of administration
- To cut down other routes of administration
- To use when I cannot or am not allowed to use via other routes of administration
- To avoid returning to other routes of administration
- Because I enjoy it
- Because it gives me a more pleasurable effect than other routes
- Because they are less harmful than other routes of administration
- Because I was concerned about my health
- Curiosity just wanted to try them
- To save money
- To protect others from second-hand exposure
- For some other reason -please explain
- Don't know
- Prefer not to say

Have you used these other substances in the last 30 days?

- Yes
- No

Past 30 other substance

Do you think your use of other substances is having a negative effect on your mental or physical health?

- Yes, on both my mental and physical health
- Yes, on my physical health only
- Yes, on my mental health only
- No

Which of the following best describes the frequency you used other substances using any route of administration in the last 30 days?

- Daily
- Less than daily, but at least once a week
- Less than weekly, but at least once a month

Do you have any current intention to quit using other substances?

- Yes
- No

Have you used an electronic vaping device to use other substances in the last 30 days?

- Yes
- No

Current use other substances

What is your main reason for vaping other substances now?

- To quit other routes of administration
- To cut down other routes of administration
- To use when I cannot or am not allowed to use via other routes of administration
- To avoid returning to other routes of administration
- Because I enjoy it
- Because it gives me a more pleasurable effect than other routes
- Because they are less harmful than other routes of administration
- Because I was concerned about my health
- Curiosity just wanted to try them
- To save money

- To protect others from second-hand exposure
- For some other reason -please explain
- Don't know
- Prefer not to say

What are any other reasons for vaping other substances now?

- To quit other routes of administration
- To cut down other routes of administration
- To use when I cannot or am not allowed to use via other routes of administration
- To avoid returning to other routes of administration
- Because I enjoy it
- Because it gives me a more pleasurable effect than other routes
- Because they are less harmful than other routes of administration
- Because I was concerned about my health
- Curiosity just wanted to try them
- To save money
- To protect others from second-hand exposure
- For some other reason -please explain
- Don't know
- Prefer not to say

Which of the following best describes the frequency you vaped other substances in the last 30 days?

- Daily
- Less than daily, but at least once a week
- Less than weekly, but at least once a month

What is the main electronic vaping device by which you use other substances ?

- Commercially bought electronic or e-cigarette
- Commercially bought electronic device used to vaporise dry herbs
- Commercially bought tabletop vapouriser
- Commercially bought electronic device used to vape liquids (but not an e-cigarette or a tabletop vapouriser)
- Homemade electronic device
- Other, please describe

Are there any other electronic vaping devices by which you use other substances ?

- ☐ Commercially bought electronic or e-cigarette
- ☐ Commercially bought electronic device used to vaporise dry herbs
- ☐ Commercially bought tabletop vapouriser
- ☐ Commercially bought electronic device used to vape liquids (but not an e-cigarette or a tabletop vapouriser)
- ☐ Homemade electronic device
- ☐ Other, please describe

Demographics

What is your age?

- ○ 18-24
- ○ 25-34
- ○ 35-44
- ○ 45-54
- ○ 55-64
- ○ 65-74
- ○ over 75

What is your sex?
Please use the sex recorded on your birth certificate or Gender Recognition Certificate

- ○ Female
- ○ Male
- ○ Prefer not to say

Is the gender you identify with the same as your sex registered at birth?

- ○ Yes
- ○ No, please give the term you use to describe your gender
- ○ Do not wish to say

What is your ethnic group?
Please choose one option that best describes your ethnic group or background

- ○

English/Welsh/Scottish/Northern Irish/British

- Irish
- Gypsy or Irish Traveller
- Any other White background, please describe
- White and Black Caribbean
- White and Black African
- White and Asian
- Any other Mixed/Multiple ethnic background, please describe
- Indian
- Pakistani
- Bangladeshi
- Chinese
- Any other Asian background, please describe
- African
- Caribbean
- Any other Black/African/Caribbean background, please describe
- Arab
- Any other ethnic group, please describe
- Do not wish to say

What is your religion?

- No religion
- Christian (including Church of England, Catholic, Protestant and all other Christian denominations)
- Buddhist
- Hindu
- Jewish
- Muslim
- Sikh
- Any other religion, please describe
- Do not wish to say

What is your legal marital or registered civil partnership status?

- Never married and never registered in a civil partnership

Supplemental material
BMJ Publishing Group Limited (BMJ) disclaims all liability and responsibility arising from any reliance placed on this supplemental material which has been supplied by the author(s)
BMJ Open

- Married
- In a registered civil partnership
- Separated, but still legally married
- Separated, but still legally in a civil partnership
- Divorced
- Formerly in a civil partnership which is now legally dissolved
- Widowed
- Surviving partner from a registered civil partnership
- Do not wish to say

Which of the following best describes your sexual orientation?

- Straight/Heterosexual
- Gay or Lesbian
- Bisexual
- Other sexual orientation, please give the term you use to describe your sexual orientation
- Do not wish to say

Have you achieved a qualification at degree level or above?

- Yes
- No
- Do not wish to say

Do you have any physical or mental health conditions or illnesses lasting or expected to last 12 months or more?

- Yes
- No
- Do not wish to say

Do any of your conditions or illnesses reduce your ability to carry out day-to-day activities?

- Yes, a lot
- Yes, a little
- Not at all

Roberts E, *et al. BMJ Open* 2023; 13:e066826. doi: 10.1136/bmjopen-2022-066826

Supplemental material
BMJ Publishing Group Limited (BMJ) disclaims all liability and responsibility arising from any reliance placed on this supplemental material which has been supplied by the author(s)
BMJ Open

Roberts E, *et al. BMJ Open* 2023; 13:e066826. doi: 10.1136/bmjopen-2022-066826
