# Supplementary material for: Pattern and prevalence of vaping nicotine and non-nicotine drugs in the United Kingdom: a cross-sectional study
Source: BMJ Open. 2023 Apr 25;13(4):e066826. doi: 10.1136/bmjopen-2022-066826 (PMC10151839; doi:10.1136/bmjopen-2022-066826)
Supplement: Supplementary data [file bmjopen-2022-066826supp001.pdf]

Online Supplementary Material

Contents

Figures

S1: The prevalence of ever vaping and current vaping of nicotine and non-nicotine drugs

Tables

S1: All non-nicotine drug types and subtypes asked about in the online cross-sectional survey

S2: Current use (within the last 30 days) profiles for all remaining non-nicotine drugs

S3: Sociodemographic breakdown of those participants who have never vaped, ever vaped any drug including nicotine and ever vaped any drug excluding nicotine

References

Figure S1: The prevalence of ever vaping and current vaping of nicotine and non-nicotine drugs

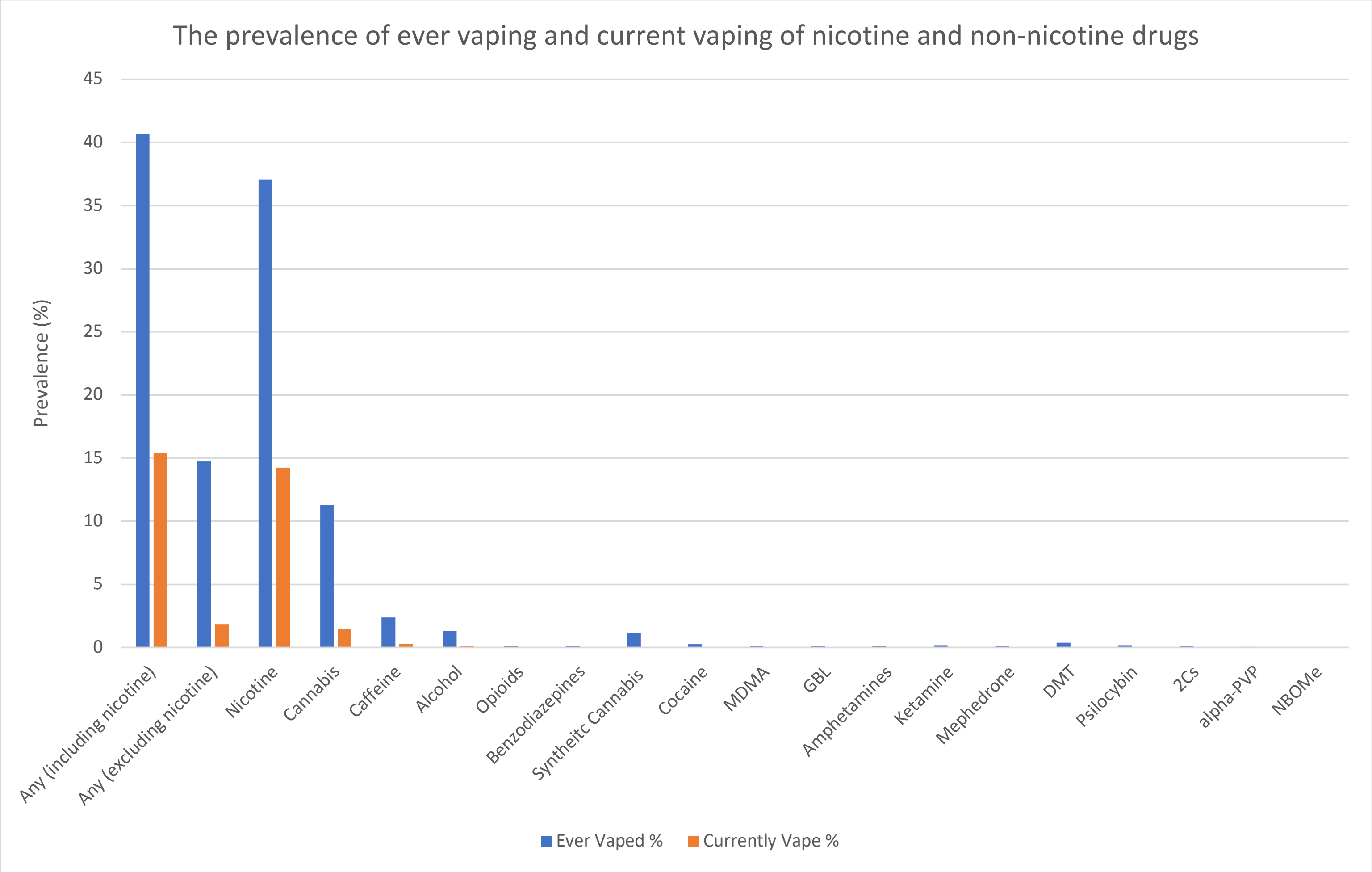

Tables

Table S1: All non-nicotine drug types and subtypes as they were detailed to participants in the online cross-sectional survey (adapted from Blundell et al. (1))

**Drug**	**Subtypes**
Nicotine	Nicotine e-liquid (freebase)<br>Nicotine salts
Caffeine	Caffeine
Alcohol	Alcohol liquid
Cannabis	Combined THC/CBD resin<br>THC resin<br>CBD resin<br>Combined THC/CBD concentrate<br>THC concentrate<br>CBD concentrate<br>Combined THC/CBD oil<br>THC oil<br>CBD oil<br>Butane hash oil<br>Dry herb/bud combined THC/CBD<br>Dry herb/bud THC<br>Dry herb/bud CBD
Synthetic Cannabis	Spice<br>Black mamba<br>Spice gold<br>Magic gold<br>Exodus<br>K2<br>Herbal incense<br>Spice arctic<br>Bonzai

**Drug**	**Subtypes**
	Annihilation<br>Thai high<br>AB-FUMINACA<br>ADB-PINACA<br>AM-2201<br>ADB-CHMICA<br>JWH-015<br>JWH-018<br>CP-47-497<br>5F-ADB<br>5F-AKB48<br>XLR-11<br>UR-144<br>HU-210<br>MDMB-CHMICA
Cocaine	Cocaine powder<br>Crack cocaine
MDMA	MDMA (aka Ecstasy Molly, Mandy)
GBL	GBL<br>GHB
Amphetamine	Ritalin (Methylphenidate)<br>Crystal methamphetamine (aka Ice, Tina, Crystal)
Opioids	Heroin<br>Morphine<br>Fentanyl
Ketamine	Ketamine (aka Special-K, Ket)
Mephedrone	Mephedrone (aka meow-meow, M-CAT)
DMT	DMT
Psilocybin	Psilocybin
Benzos	Diazepam (aka blues, Valium)<br>Clonazepam (aka Rivotril)<br>Alprazolam (aka Xanax)

**Drug**	**Subtypes**
2Cs	2CB<br>2CE
alpha-PVP	alpha-PVP aka gravelflakka
NBOMe	NBOMe (aka N-Bomb)
Other	Other, please describe

Table S2: Current use (within the last 30 days) profiles for all remaining non-nicotine drugs

	Ketamine	Synthetic Cannabis	Cocaine	Amphetamines	MDMA	GBL	Mephedrone	DMT	Psilocybin	2Cs	alpha-PVP	NBOMe
Currently use n (%)	1 (100%)	2 (100%)	2 (100%)	2 (100%)	0	0	0	0	0	0	0	0
Frequency of current use n (%)												
Daily	0 (0.0%)	0 (0.0%)	0 (0.0%)	1 (50.0%)	-	-	-	-	-	-	-	-
Weekly	0 (0.0%)	1 (50.0%)	1 (50.0%)	1 (50.0%)	-	-	-	-	-	-	-	-
Monthly	1 (100.0%)	1 (50.0%)	1 (50.0%)	0 (0.0%)	-	-	-	-	-	-	-	-
Currently use harmfully n (%)	0 (0.0%)	0 (0.0%)	1 (50.0%)	2 (100.0%)	-	-	-	-	-	-	-	-
Currently vape n (%)	0 (0.05)	0 (0.0%)	0 (0.0%)	0 (0.0%)	-	-	-	-	-	-	-	-
Frequency of current vaping n (%)												
Daily	-	-	-	-	-	-	-	-	-	-	-	-
Weekly	-	-	-	-	-	-	-	-	-	-	-	-
Monthly	-	-	-	-	-	-	-	-	-	-	-	-
Age first used mean (SE)	21.3 (1.38)	26.6 (1.37)	23.4 (2.14)	22.8 (3.12)	25.5 (5.17)	28 (2.74)	31.3 (4.81)	27.0 (2.51)	18.6 (1.02)	28.5 (4.14)	18.5 (3.40)	30 (N/A)
Age first vaped mean (SE)	24.4 (2.22)	27.0 (1.41)	31.3 (2.94)	25.8 (3.43)	26.5 (4.86)	30.5 (5.04)	31.7 (4.91)	27.2 (2.48)	27.4 (4.17)	35.7 (5.90)	18.5 (3.50)	30 (N/A)
t, P[1]	-1.9, 0.1	-2.3, 0.02	-2.7, 0.03	-1.4, 0.23	-2.2, 0.08	-1.0, 0.39	-1.0, 0.42	-1.0, 0.33	-1.9, 0.11	-1.1, 0.31	N/A	N/A
Modal reason for vaping initially	Because I thought I would enjoy it (42.8%)	Curiosity just wanted to try (31.1%)	Curiosity just wanted to try (30.0%)	Because I thought I would enjoy it (40.0%)	Because I thought I would enjoy it (50.0%)	Because I thought I would enjoy it (50.0%)	Curiosity just wanted to try (33.3%)	Curiosity just wanted to try (50.0%)	Curiosity just wanted to try (57.1%)	To avoid returning to other routes of administration (33.3%)	To quit other routes of administration (100.0%)	Don’t know (100.0%)
Stated an intention to quit use n (%)	0 (0.0%)	0 (0.0%)	0 (0.0%)	1 (50.0%)	-	-	-	-	-	-	-	-

1 Compared using an unpaired t-test

Table S3: Sociodemographic breakdown of those participants who have never vaped, ever vaped any drug including nicotine and ever vaped any drug excluding nicotine

		All n (%)	Never vaped n (%)	Ever vaped any (including nicotine) n (%)	Ever vaped any (excluding nicotine) n (%)	Ever vaped any non-nicotine compared to never vapers (p)[1]
All	All	4027 (100%)	2,390 (100%)	1,637 (100%)	593 (100%)	-
Gender identity	Gender identity is the same as their sex registered at birth	3984 (98.9%)	2369 (99.1%)	1615 (98.7%)	580 (97.8%)	0.02
	Gender identity is not the same as their sex registered at birth	26 (0.7%)	12 (0.5%)	14 (0.8%)	9 (1.5%)	
	Do not wish to say	17 (0.4%)	9 (0.4%)	8 (0.5%)	4 (0.7%)	
Sexual orientation	Straight/Heterosexual	3548 (88.1%)	2176 (91.1%)	1372 (83.8%)	453 (76.4%)	<0.001
	Gay or Lesbian	153 (3.8%)	69 (2.9%)	84 (5.1%)	34 (5.7%)	
	Bisexual	237 (5.9%)	90 (3.8%)	147 (9.0%)	89 (15.0%)	
	Other sexual orientation	42 (1.0%)	26 (1.1%)	16 (1.0%)	9 (1.5%)	
	Do not wish to say	47 (1.2%)	29 (1.2%)	18 (1.1%)	8 (1.4%)	
Religion	No religion	2502 (62.1%)	1403 (58.7%)	1099 (67.1%)	424 (71.5%)	<0.001[2]
	Christian (including Church of England, Catholic, Protestant and all other Christian denominations)	1233 (30.6%)	820 (34.3%)	413 (25.2%)	112 (18.9%)	
	Buddhist	25 (0.6%)	12 (0.5%)	13 (0.8%)	6 (1.0%)	
	Hindu	34 (0.8%)	14 (0.6%)	20 (1.2%)	9 (1.5%)	
	Jewish	21 (0.5%)	13 (0.5%)	8 (0.5%)	4 (0.7%)	
	Muslim	113 (2.8%)	72 (3.0%)	41 (2.5%)	16 (2.7%)	
	Sikh	13 (0.3%)	9 (0.4%)	4 (0.2%)	1 (0.2%)	
	Any other religion	30 (0.7%)	17 (0.7%)	13 (0.8%)	10 (1.7%)	
	Do not wish to say	56 (1.4%)	30 (1.3%)	26 (1.6%)	11 (1.9%)	
Marital status	Never married and never registered in a civil partnership	1980 (49.2%)	1052 (44.0%)	928 (56.7%)	376 (63.4%)	<0.001[3]
	Married	1580 (39.2%)	1088 (45.5%)	492 (30.1%)	152 (25.6%)	
	In a registered civil partnership	56 (1.4%)	19 (0.8%)	37 (2.2%)	12 (2.0%)	
	Separated, but still legally married	51 (1.3%)	22 (0.9%)	29 (1.8%)	9 (1.5%)	
	Separated, but still legally in a civil partnership	3 (0.1%)	2 (0.1%)	1 (0.1%)	1 (0.2%)	
	Divorced	247 (6.1%)	147 (6.2%)	100 (6.1%)	30 (5.0%)	
	Formerly in a civil partnership which is now legally dissolved	4 (0.1%)	2 (0.1%)	2 (0.1%)	0 (0.0%)	
	Widowed	46 (1.1%)	30 (1.3%)	16 (1.0%)	2 (0.3%)	
	Surviving partner from a registered civil partnership	2 (0.1%)	1 (0.1%)	1 (0.1%)	0 (0.0%)	
	Do not wish to say	58 (1.4%)	27 (1.1%)	31 (1.9%)	11 (1.9%)	
Disability	Has a condition or illnesses which reduces their ability to carry out day-to-day activities	721 (17.9%)	358 (15.0%)	363 (22.2%)	163 (27.5%)	<0.001
	Does not have a condition or illnesses which reduces their ability to carry out day-to-day activities	3214 (79.8%)	1983 (83.0%)	1231 (75.2%)	413 (69.6%)	
	Do not wish to say	92 (2.3%)	49 (2.1%)	43 (2.6%)	17 (2.9%)	
Level of education	Has achieved a qualification at degree level or above	2506 (62.2%)	1526 (63.9%)	980 (59.9%)	393 (66.3%)	0.51
	Has not achieved a qualification at degree level or above	1489 (37.0%)	846 (35.4%)	643 (39.3%)	195 (32.9%)	
	Do not wish to say	32 (0.8%)	18 (0.8%)	14 (0.9%)	5 (0.8%)	

1 Compared using Fishers exact test; 2 For the purpose of statistical testing this category was collapsed into three levels: No religion, Christian (including Church of England, Catholic, Protestant and all other Christian denominations) and any other religion (Buddhist, Hindu, Jewish, Muslim, Sikh, Any other religion) 3 For the purpose of statistical testing this category was collapsed into three levels; Never married and never registered in a civil partnership, Married or in a registered civil partnership and any form of dissolution (Separated, but still legally married, Separated, but still legally in a civil partnership Divorced, Formerly in a civil partnership which is now legally dissolved, Widowed or Surviving partner from a registered civil partnership)

References

1. Blundell M, Dargan P, Wood D. A cloud on the horizon–a survey into the use of electronic vaping devices for recreational drug and new psychoactive substance (NPS) administration. QJM: An International Journal of Medicine. 2018;111(1):9-14.
