# Supplementary material for: Pattern and prevalence of vaping nicotine and non-nicotine drugs in the United Kingdom: a cross-sectional study
Source: BMJ Open. 2023 Apr 25;13(4):e066826. doi: 10.1136/bmjopen-2022-066826 (PMC10151839; doi:10.1136/bmjopen-2022-066826)
Supplement: Author's manuscript [file bmjopen-2022-066826.draft_revisions.pdf]

BMJ Open

BMJ Open is committed to open peer review. As part of this commitment we make the peer review history of every article we publish publicly available.

When an article is published we post the peer reviewers' comments and the authors' responses online. We also post the versions of the paper that were used during peer review. These are the versions that the peer review comments apply to.

The versions of the paper that follow are the versions that were submitted during the peer review process. They are not the versions of record or the final published versions. They should not be cited or distributed as the published version of this manuscript.

BMJ Open is an open access journal and the full, final, typeset and author-corrected version of record of the manuscript is available on our site with no access controls, subscription charges or pay-per-view fees (http://bmjopen.bmj.com).

If you have any questions on BMJ Open's open peer review process please email info.bmjopen@bmj.com

BMJ Open

The pattern and prevalence of vaping nicotine and non-nicotine drugs in the United Kingdom: A cross-sectional study

Journal:	*BMJ Open*
Manuscript ID	bmjopen-2022-066826
Article Type:	Original research
Date Submitted by the Author:	21-Jul-2022
Complete List of Authors:	Roberts , Emmert; King's College London, National Addiction Centre; South London and Maudsley NHS Foundation Trust,<br>Taylor, Eve; King's College London<br>Cox, Sharon; University College London, Behavioural Science and Health; SPECTRUM Research Consortium,<br>Brose, Leonie; King's College London, Addictions Department<br>McNeill, Ann; King’s College London, UK Centre for Tobacco Control Studies, National Addiction Centre, Institute of Psychiatry<br>Robson, Deborah; Kings College London, Psychiatry
Keywords:	EPIDEMIOLOGY, Substance misuse < PSYCHIATRY, PUBLIC HEALTH

SCHOLARONE™
Manuscripts

BMJ

I, the Submitting Author has the right to grant and does grant on behalf of all authors of the Work (as defined in the below author licence), an exclusive licence and/or a non-exclusive licence for contributions from authors who are: i) UK Crown employees; ii) where BMJ has agreed a CC-BY licence shall apply, and/or iii) in accordance with the terms applicable for US Federal Government officers or employees acting as part of their official duties; on a worldwide, perpetual, irrevocable, royalty-free basis to BMJ Publishing Group Ltd ("BMJ") its licensees and where the relevant Journal is co-owned by BMJ to the co-owners of the Journal, to publish the Work in this journal and any other BMJ products and to exploit all rights, as set out in our licence.

The Submitting Author accepts and understands that any supply made under these terms is made by BMJ to the Submitting Author unless you are acting as an employee on behalf of your employer or a postgraduate student of an affiliated institution which is paying any applicable article publishing charge ("APC") for Open Access articles. Where the Submitting Author wishes to make the Work available on an Open Access basis (and intends to pay the relevant APC), the terms of reuse of such Open Access shall be governed by a Creative Commons licence – details of these licences and which Creative Commons licence will apply to this Work are set out in our licence referred to above.

Other than as permitted in any relevant BMJ Author's Self Archiving Policies, I confirm this Work has not been accepted for publication elsewhere, is not being considered for publication elsewhere and does not duplicate material already published. I confirm all authors consent to publication of this Work and authorise the granting of this licence.

For peer review only

Title: The pattern and prevalence of vaping nicotine and non-nicotine drugs in the United Kingdom: A cross-sectional study

Authors:

Dr Emmert Roberts, National Addiction Centre, Institute of Psychiatry, Psychology and Neuroscience, King's College London, 4 Windsor Walk, London, SE5 8AF, United Kingdom and the South London and the Maudsley NHS Foundation Trust, Denmark Hill, London, United Kingdom SE5 8AZ

Eve Taylor, National Addiction Centre, Psychology and Neuroscience, King's College London, 4 Windsor Walk, London, SE5 8AF, United Kingdom

Dr Sharon Cox, Institute of Epidemiology & Health, Faculty of Population Health Sciences, University College London, 30 Guilford Street London WC1N 1EH

Dr Leonie Brose, National Addiction Centre, Institute of Psychiatry, Psychology and Neuroscience, King's College London, United Kingdom 4 Windsor Walk, London, SE5 8AF, United Kingdom

Professor Ann McNeill, National Addiction Centre, Institute of Psychiatry, Psychology and Neuroscience, King's College London, 4 Windsor Walk, London, SE5 8AF, United Kingdom

Dr Debbie Robson, National Addiction Centre, Institute of Psychiatry, Psychology and Neuroscience, King's College London, 4 Windsor Walk, London, SE5 8AF, United Kingdom

Corresponding author:

Dr Emmert Roberts
National Addiction Centre, Psychology and Neuroscience, King's College London, 4 Windsor Walk, London, SE5 8AF, United Kingdom
+447754156145
emmert.roberts@kcl.ac.uk

Running Head:

Vaping nicotine and non-nicotine drugs in UK

For peer review only - http://bmjopen.bmj.com/site/about/guidelines.xhtml

Word Count: 1942

For peer review only

For peer review only - http://bmjopen.bmj.com/site/about/guidelines.xhtml

Abstract (Word Count: 296) (Max 300)

Objectives: Electronic vaping devices are being used to consume nicotine and non-nicotine drugs. We aimed to determine the pattern and prevalence of using vaping devices for nicotine and/or non-nicotine drug administration in the United Kingdom and how these differ by drug type and individual sociodemographic characteristics. We explored reasons for vaping onset and continuation.

Design: An online cross-sectional survey

Participants: A convenience sample of adults in the UK

Primary and Secondary Outcome Measures: The primary outcome was prevalence of current use (within the last 30 days) of a vaping device to administer either nicotine or eighteen types of non-nicotine drug. We additionally evaluated reasons for onset and continuation of vaping. Sociodemographic and vaping characteristics were compared between the UK general population using census data and those vaping non-nicotine drugs.

Results: We recruited 4027 participants of whom 1637 (40.7%) had ever used an electronic vaping device; 1495 (37.1%) had ever vaped nicotine and 593 (14.7%) had ever vaped a non-nicotine drug. Overall, 574 (14.3%) currently vaped nicotine and 74 (1.8%) currently vaped a non-nicotine drug. The most common currently vaped non-nicotine drug was cannabis (n=58, 1.4%). For nicotine, people's modal reasons to start and continue vaping was to quit smoking tobacco. For almost all other drugs people's modal reason to start vaping was curiosity and to continue was enjoyment. Compared to the general population, the population who had ever vaped a non-nicotine drug were significantly younger, more male, had more disabilities and fewer identified as white, heterosexual or religious.

Conclusions: A non-trivial number of people report current use and ever use of an electronic vaping device for non-nicotine drug administration. As vaping technology advances and drug consumption changes, understanding patterns of use and associated behaviours is likely to be increasingly important to both users and healthcare professionals.

For peer review only - http://bmjopen.bmj.com/site/about/guidelines.xhtml

Strengths and limitations of this study

- The study reports, to our knowledge, the largest cross-sectional sample in the published literature of people ever vaping a range of non-nicotine drugs.
- The study reports, to our knowledge, the only evaluation of the socio-demographic profile of people using electronic vaping devices to consume non-nicotine drugs, alongside people's reasons for onset and continuation of vaping, and the type of devices they use.
- The study relied on self-report to identify non-nicotine drug use and may be subject to social desirability bias.
- The study was conducted using an online convenience sample and several populations who have historically higher rates of non-nicotine drug consumption may have been excluded due to the study's digital nature or other life circumstances (e.g., people experiencing homelessness or currently serving a prison sentence).

For peer review only

Introduction

In the last decade the advent of electronic nicotine vaping products has changed how some people use nicotine and recent reports also suggest use of electronic vaping devices to administer non-nicotine drugs. (1-3) As innovation in technology develops, the prevalence and patterns of using electronic vaping devices to facilitate non-nicotine drug use are subject to change. (4) Whilst in some circumstances vaping non-nicotine drugs may present fewer harms when compared to other routes of administration (e.g., smoking) in some cases vaping could potentially be more harmful due to the speed of onset of drug effects, alongside the concomitant use of any adulterants. (4-6) Most notably the 2019 United States outbreak of acute lung injury associated with vaping illicit cannabinoid products contaminated with vitamin E acetate highlights the potential for associated harms. (7)

Monitoring of nicotine vaping in the United Kingdom is well documented, (8) whilst data on vaping of non-nicotine drugs is sparse. (9) Understanding the prevalence and patterns of vaping non-nicotine drugs is important for healthcare professionals to both facilitate discussions regarding consumers' preferred routes of drug administration, and to provide appropriate harm reduction advice. Whilst a recent review of online drug user forums has identified over 300 reports related to the manufacture and/or use of e-liquids containing non-nicotine drugs, (2) few epidemiological studies have been conducted to determine the prevalence and patterns of using electronic vaping devices to administer different non-nicotine drugs. Although some studies have examined vaping in the context of nicotine and cannabis administration, (9) to our knowledge, only a single cross-sectional study conducted in 2017 in adults in the United Kingdom (UK) reported that 340 participants (13.6%) had ever used an electronic vaping device for any type of recreational drug administration during their lifetime. (3) However, this estimate has not been reproduced, the sociodemographic characteristics of the cohort of people vaping non-nicotine drugs have not been fully evaluated and there has been little examination of why people use vaping as a route of non-nicotine drug administration.

To address this gap, we aimed to determine the pattern and prevalence of using electronic vaping devices for nicotine and non-nicotine drug administration in the UK and how these differ by drug type and individual sociodemographic characteristics. In addition, we aimed to examine individual reasons for the onset of vaping non-nicotine drugs and why people continue vaping as a route of administration.

For peer review only

Methods

An anonymous voluntary cross-sectional survey recruiting a convenience sample of adult participants (aged ≥18 years) through the online Prolific platform. (10) Prolific advertised the study to all adults on their platform and all data were collected on March 10th 2022. The study protocol, questionnaire and statistical analysis plan were pre-registered on the Open Science Framework (OSF) and are available at https://osf.io/bdfhp/.

The primary outcome was the prevalence of current use of a vaping device to administer either nicotine or eighteen specific types of non-nicotine drugs, defined as use in the past 30 days (A complete list of all non-nicotine drug types and subtypes studied can be found in the online supplementary material as table S1). Secondary outcomes included prevalence of ever using a vaping device to administer either nicotine or non-nicotine drugs and current use of nicotine or non-nicotine drugs by routes of administration other than vaping. We additionally collected if participants considered any currently consumed drug was having a negative consequence on their mental or physical health (harmful use) and on frequency of current use and vaping, age of first use and vaping onset, reasons for onset of vaping and why people continue vaping as a route of administration.

Individual UK census defined sociodemographic characteristics were also collected including level of educational attainment and eight 'protected' characteristics as defined by the UK Equality Act (age, sex, disability, gender identity, sexual orientation, ethnicity, religion and marital status). (11)

Sociodemographic and vaping characteristics were compared between those that had used an electronic vaping device to vape a non-nicotine drug, those who had never used an electronic vaping device and the UK general population (using publicly available data from the UK census). (12) Continuous variables were compared using an unpaired t-test, and categorical variables using Fisher's exact test. All analyses were conducted using STATA SE version 15.1 with the significance level set at 0.05. The study was reported in accordance with the STROBE statement for observational studies. (13)

Patient and Public Involvement

No patients or public involvement

For peer review only - http://bmjopen.bmj.com/site/about/guidelines.xhtml

Results

There were a total of 4027 participants of whom 1637 (40.7%) had ever used an electronic vaping device; 1495 people (37.1%) had ever vaped nicotine and 593 (14.7%) had ever vaped a non-nicotine drug. 1044 (25.9%) had only ever exclusively vaped nicotine, 142 (3.5%) had only ever exclusively vaped a non-nicotine drug and 451 (11.2%) had ever vaped both nicotine and a non-nicotine drug (Table 1)

For peer review only

For peer review only - http://bmjopen.bmj.com/site/about/guidelines.xhtml

table 1: The age, sex and ethnicity breakdown of those participants who have never vaped, ever vaped any drug including nicotine and ever vaped any non-nicotine drug

Sociodemographic characteristic		All n (%)	Never vaped n (%)	Ever vaped any drug (including nicotine) n (%)	Ever vaped any non-nicotine drug n (%)	Ever vaped any non-nicotine drug compared to never vapers (p)[1]
All	All	4027 (100%)	2390 (100%)	1637 (100%)	593 (100%)	-
Age	18-24	441 (11.0%)	199 (8.3%)	242 (14.8%)	112 (18.9%)	<0.001[2]
	25-34	1265 (31.4%)	670 (28.0%)	595 (36.4%)	244 (41.2%)	
	35-44	1067 (26.5%)	632 (26.4%)	435 (26.6%)	146 (24.6%)	
	45-54	629 (15.6%)	410 (17.2%)	219 (13.4%)	57 (9.6%)	
	55-64	428 (10.6%)	319 (12.4%)	109 (6.7%)	25 (4.2%)	
	65-74	171 (4.3%)	138 (5.8%)	33 (2.0%)	8 (1.4%)	
	over 75	26 (0.7%)	22 (0.9%)	4 (0.2%)	1 (0.2%)	
Sex	Male	1400 (34.8%)	754 (31.2%)	655 (40.1%)	271 (45.7%)	<0.001
	Female	2614 (64.9%)	1641 (68.7%)	973 (59.4%)	317 (53.5%)	
	Do not wish to say	13 (0.3%)	4 (0.2%)	9 (0.6%)	5 (0.8%)	
Ethnicity	English/Welsh/Scottish/ Northern Irish/British	3254 (80.8%)	1952 (81.7%)	1302 (79.5%)	437 (73.7%)	<0.001[3]
	Irish	42 (1.0%)	27 (1.1%)	15 (0.9%)	9 (1.5%)	
	Gypsy or Irish Traveller	2 (0.1%)	0 (0.0%)	2 (0.1%)	1 (0.2%)	
	Any other White background	227 (5.6%)	128 (5.4%)	99 (6.1%)	42 (7.1%)	
	White and Black Caribbean	37 (0.9%)	17 (0.7%)	20 (1.2%)	7 (1.2%)	
	White and Black African	14 (0.4%)	8 (0.3%)	6 (0.4%)	4 (0.7%)	
	White and Asian	38 (0.9%)	14 (0.6%)	24 (1.5%)	15 (2.5%)	
	Any other Mixed/ Multiple ethnic background	21 (0.5%)	12 (0.5%)	9 (0.6%)	4 (0.7%)	
	Indian	78 (1.9%)	42 (1.7%)	36 (2.2%)	16 (2.7%)	
	Pakistani	52 (1.3%)	27 (1.1%)	25 (1.5%)	11 (1.9%)	
	Bangladeshi	17 (0.4%)	12 (0.5%)	5 (0.3%)	2 (0.3%)	
	Chinese	58 (1.4%)	37 (1.6%)	21 (1.3%)	12 (2.0%)	
	Any other Asian background	24 (0.6%)	13 (0.5%)	11 (0.7%)	5 (0.8%)	
	African	69 (1.7%)	52 (2.2%)	17 (1.0%)	7 (1.2%)	
	Caribbean	29 (0.7%)	18 (0.8%)	11 (0.7%)	3 (0.5%)	
	Any other Black/African/ Caribbean background	5 (0.1%)	1 (0.1%)	4 (0.2%)	2 (0.3%)	
	Arab	14 (0.4%)	7 (0.3%)	7 (0.4%)	4 (0.7%)	
	Any other ethnic group	21 (0.5%)	8 (0.3%)	13 (0.8%)	7 (1.2%)	
	Do not wish to say	25 (0.6%)	15 (0.6%)	10 (0.6%)	5 (0.8%)	

1 Compared using Fishers exact test; 2 For the purpose of statistical testing this category was collapsed into five levels: 18-24, 25-34, 35-44, 45-54 and over 55 3 For the purpose of statistical testing this category was collapsed into two levels: white (English/Welsh/Scottish/Northern Irish/British, Irish, Gypsy or Irish Traveller or any other White background) and non-white (White and Black Caribbean, White and Black African, White and Asian, Any other Mixed/Multiple ethnic background, Indian, Pakistani, Bangladeshi, Chinese, Any other Asian background, African, Caribbean, Any other Black/African/Caribbean background, Arab or any other ethnic group)

For peer review only

About a fifth (n=834, 20.7%) were currently using a nicotine product; 595 (14.8%) were currently smoking tobacco, 574 (14.3%) were currently vaping nicotine and 280 (7.0%) were currently concurrently smoking tobacco and vaping nicotine. Fewer (n=217, 5.4%) participants were currently using a non-nicotine drug, of whom 74 (1.8%) were currently vaping a non-nicotine drug. The only non-nicotine drugs currently being vaped were cannabis 58 (1.4%), caffeine 12 (0.3%), alcohol 5 (0.1%), opioids 1 (0.02%) and benzodiazepines 1 (0.02%).

Participants' mean age at first use by any route was statistically significantly lower than their mean age at first vaping for nicotine, alcohol, caffeine, cannabis, synthetic cannabinoids and cocaine. For nicotine, people's modal reason to try vaping and their current reason to vape was to quit smoking tobacco. For almost all other drugs, people's modal reason to try vaping was curiosity and their modal reason to currently vape was because of enjoyment. The modal type of electronic vaping device currently used was a commercially bought electronic or e-cigarette for all studied drugs except cannabis for which the model type of device currently used was a commercially bought electronic device to vaporise dry herbs. A profile of current use for those drugs currently being vaped is available in table 2 with a breakdown for all other drugs available in the online supplementary material as table S2.

For peer review only

table 2: Current use profiles for all drugs which participants are currently vaping (within the last 30 days)

	Any (including nicotine)	Any non-nicotine drug	Nicotine	Caffeine	Alcohol	Cannabis	Opioids	Benzodiazepines
Currently use n (%)	929 (100%)	217 (100%)	834 (100%)	45 (100%)	29 (100%)	146 (100%)	2 (100%)	1 (100%)
Frequency of current use n (%)								
Daily	-	-	601 (72.1%)	28 (62.2%)	4 (13.8%)	58 (39.7%)	1 (50.0%)	0 (0.0%)
Weekly	-	-	118 (14.2%)	16 (35.6%)	14 (48.3%)	45 (30.8%)	1 (50.0%)	0 (0.0%)
Monthly	-	-	115 (13.8%)	1 (2.2%)	11 (37.9%)	43 (29.5%)	0 (0.0%)	1 (100.0%)
Currently use harmfully n (%)	542 (58.3%)	73 (33.6%)	508 (60.9%)	14 (31.1%)	13 (44.8%)	47 (32.2%)	1 (50.0%)	0 (0.0%)
Currently vape n (%)	621 (68.8%)	74 (34.1%)	574 (68.8%)	12 (26.7%)	5 (17.2%)	58 (39.7%)	1 (50.0%)	1 (100.0%)
Frequency of current vaping n (%)								
Daily	-	-	363 (43.5%)	7 (15.6%)	0 (0.0%)	14 (9.6%)	0 (0.0%)	0 (0.0%)
Weekly	-	-	130 (15.6%)	4 (8.9%)	2 (6.9%)	22 (15.1%)	1 (50.0%)	1 (100.0%)
Monthly	-	-	81 (9.7%)	1 (2.2%)	3 (10.3%)	22 (15.1%)	0 (0.0%)	0 (0.0%)
Age first used mean (SE)	17.2 (0.15)	19.8 (0.36)	17.1 (0.15)	22.3 (1.24)	18.3 (1.09)	19.1 (0.34)	24.2 (5.40)	25.0 (4.56)
Age first vaped mean (SE)	29.8 (0.28)	27.9 (0.42)	30.2 (0.30)	27.6 (1.83)	24.6 (1.4)	28.1 (0.46)	35.3 (5.90)	29.8 (7.86)
t, p[1]	-41.5, <0.001	-20.3, <0.001	-41.2, <0.001	-6.8, <0.001	-4.3, <0.001	-19.7, <0.001	-2.2, 0.08	-1.3, 0.27
Modal reason for vaping initially	-	-	To quit smoking tobacco (31.7%)	Curiosity just wanted to try (37.5%)	Curiosity just wanted to try (50.9%)	Curiosity just wanted to try (41.6%)	Curiosity just wanted to try (50.0%)	Curiosity just wanted to try (25.0%)
Modal reason for vaping currently	-	-	To quit smoking tobacco (23.0%)	Because I enjoy it (41.7%)	Because I enjoy it (60.0%)	Because I enjoy it (34.5%)	For some other reason (100.0%)	Because I enjoy it (100.0%)
Stated an intention to quit n (%)	567 (61.0%)	53 (24.4%)	541 (64.9%)	14 (31.1%)	9 (31.0%)	29 (19.9%)	1 (50.0%)	1 (100%)
Modal type of electronic vaping device currently used (%)	-	-	Commercially bought e-cigarette (86.4%)	Commercially bought e-cigarette (75.0%)	Commercially bought e-cigarette (40.0%)	Commercially bought electronic device used to vaporise dry herbs (53.5%)	Commercially bought e-cigarette (100.0%)	Commercially bought e-cigarette (100.0%)

1 Compared using an unpaired t-test

For peer review only - http://bmjopen.bmj.com/site/about/guidelines.xhtml

Of the 593 people who had ever vaped a non-nicotine drug, 484 (12.0% of the overall sample) had only ever vaped one type of non-nicotine drug and 109 (2.7%) had vaped two or more types of non-nicotine drugs. The most frequently ever vaped non-nicotine drug was cannabis (n=454, 11.3%), of whom 129 (3.2%) people had only ever vaped cannabidiol (CBD) products. This was followed by caffeine (n=96, 2.4%), alcohol (n=53, 1.3%), synthetic cannabinoids (n=45, 1.1%), Dimethyltryptamine (DMT) (n=16, 0.4%), cocaine (n=10, 0.2%), psilocybin (n=7, 0.2%), ketamine (n=7, 0.2%), opioids (n=6, 0.1%), MDMA (n=6, 0.1%), 2Cs (n=6, 0.1%), amphetamines (n=5, 0.1%), Gamma-butyrolactone (GBL) (n=4, 0.1%), benzodiazepines (n=4, 0.1%), mephedrone (n=3, 0.1%), alpha-PVP (n=2, 0.1%) and NBOMe (n=1, 0.02%). A bar chart depicting the prevalence of ever and current vaping for each drug is available in the online supplementary material as figure S1.

When compared to both the UK general population and to people who had never vaped, those who had ever vaped a non-nicotine drug were significantly different in every studied protected characteristic sociodemographic category. This group was significantly younger, had a higher proportion of males, there were fewer people identifying with white ethnicities, there were more individuals who identified as LGBT+, more non-religious individuals, more people with disabilities, and fewer married people. However, there was no statistically significant difference by level of educational attainment. A breakdown by age, sex and ethnicity can be found in table 1, with the remaining sociodemographic differences available in the online supplementary material as table S3.

For peer review only

For peer review only - http://bmjopen.bmj.com/site/about/guidelines.xhtml

Discussion

In this study of 4027 respondents, 1.8% reported they were currently vaping a non-nicotine drug and 14.7% reported ever having vaped a non-nicotine drug. About one in six respondents were currently vaping nicotine around half of whom were also smoking tobacco.

The current prevalence of smoking tobacco was similar to recent estimates (14.8% vs 13.8%-16.0% in 2020), (8) whilst the current prevalence of vaping nicotine was considerably higher (14.3% vs 5.5%-6.3% in 2020), (8) perhaps reflecting sampling differences or that the day prior to data collection was national 'No Smoking Day' in the UK.

The prevalence of ever using a vaping device to administer a non-nicotine drug was similar to estimates from a previous study (14.7% vs. 13.6%), (3) however prevalence estimates of currently vaping a non-nicotine drug were substantially lower (1.8% vs. 9.4%). (3, 9) This could be explained by sampling differences or may perhaps demonstrate that current prevalence may fluctuate dependent on technological or other changes in the vaping device market with new products potentially leading to curiosity-driven experimentation that may not be sustained over time. (14) Temporal prevalence trends are likely to reveal more about these relationships than isolated cross-sectional sampling.

When compared to the general population, the non-nicotine drug vaping population were significantly different across all studied protected characteristics, however their sociodemographic profile was broadly comparable to those of non-nicotine drug users in publicly available UK cross-sectional data, e.g., the Crime Survey for England and Wales. (15) Participants' mean age at first use by any route was consistently lower than respondents mean age at first vaping for all studied drugs, although lack of statistical power may account for the fact this was only significant for a few substances. This suggests that vaping is generally a rare first route of drug administration.

Similar to other studies, cannabis was the most commonly vaped non-nicotine drug with 11.3% of total respondents having ever vaped a cannabis product, however just over a quarter of respondents had only ever vaped a product containing CBD which, at the time of the survey, was legal to purchase and consume in the UK. (3, 16) This underscores vaping should routinely be enquired about as a potential method of administration among cannabis users, and any future work on understanding relative cannabis-related harms should include vaping when considering routes of administration. Concerningly 45 individuals reported

For peer review only

For peer review only - http://bmjopen.bmj.com/site/about/guidelines.xhtml

vaping synthetic cannabinoids, which have been linked to two vaping product related deaths in the UK. (6)

Whilst the study is, to our knowledge, the largest cross-sectional sample in the published literature of people ever vaping a range of non-nicotine drugs there are several limitations. The study relied on self-report to identify drug use and was conducted using an online convenience sample. Several populations who have historically higher rates of non-nicotine drug consumption may have been excluded due to the study's digital nature or other life circumstances (e.g., people experiencing homelessness or currently serving a prison sentence). This is particularly important given the harms associated with drug use are disproportionately higher within these populations. (6) Additionally, despite the survey questions stating that flavoured e-liquids such as coffee and alcohol flavours do not contain either caffeine or alcohol, we cannot be sure that respondents did not misinterpret these questions and thus reported vaping prevalence results for alcohol and caffeine may represent overestimates.

Understanding people's experiences and perceptions of vaping as a means of administering non-nicotine drugs is likely to be increasingly important both to users and to healthcare and policy professionals. This study demonstrates that a non-trivial number of people have ever consumed non-nicotine drugs using vaping devices and understanding how this prevalence changes over time and which individuals may be most at risk of harm or benefit from this administration method requires further exploration. Future research should be conducted in larger non-UK samples, examine any potential trends in use of vaping products to administer non-nicotine drugs and examine vaping in samples known to experience disproportionate drug-related harms.

For peer review only

Ethics Approval

Informed consent was obtained from all participants and ethical approval gained from the King's College London Health Faculties Research Ethics Subcommittee (Reference: HR-21/22-26249).

Contributorship statement:

ER: Conceptualization; Methodology; Investigation; Data curation; Formal analysis; Project administration; Supervision; Funding acquisition; Writing - original draft.
ET: Methodology; Investigation; Data curation; Formal analysis; Project administration; Supervision; Writing - review & editing.
SC: Investigation; Writing - review & editing.
LB: Investigation; Writing - review & editing.
AM: Conceptualization; Supervision; Writing - review & editing.
DR: Conceptualization; Methodology; Investigation; Supervision; Funding acquisition; Writing - review & editing.

Funding:

This study was supported by the National Institute of Health Research (NIHR) Biomedical Research Centre at South London and Maudsley NHS Foundation Trust and King's College London. ER is funded by a Medical Research Council (MRC) Addiction Research Clinical Fellowship (MR/N00616X/1). SC receives salary support from Cancer Research UK (PRCRPG-Nov21\100002). DR is supported by the National Institute for Health Research (NIHR) Applied Research Collaboration (ARC) South London at King's College Hospital NHS Foundation Trust. AM, DR and LB are members of SPECTRUM, a UK Prevention Research Partnership Consortium. UKPRP is an initiative funded by the UK Research and Innovation Councils, the Department of Health and Social Care (England) and the UK devolved administrations, and leading health research charities. AM is a National Institute for Health and Care Research (NIHR) Senior Investigator. The funders had no role in: study design, in the collection, analysis, or interpretation of data; in the writing of the report; or in the decision to submit the paper for publication. The views expressed in this article are those of the authors and not necessarily those of the MRC, NIHR, or the Department of Health and Social Care.

Competing Interests:

For peer review only - http://bmjopen.bmj.com/site/about/guidelines.xhtml

All authors have completed the ICJME Unified Competing Interest form (available on request from the corresponding author) and declare: None

Data sharing statement:

The study protocol and questionnaire are available at https://osf.io/bdfhp/

For peer review only

References

1. Gravely S, Driezen P, Ouimet J, Quah AC, Cummings KM, Thompson ME, et al. Prevalence of awareness, ever-use and current use of nicotine vaping products (NVPs) among adult current smokers and ex-smokers in 14 countries with differing regulations on sales and marketing of NVPs: cross-sectional findings from the ITC Project. Addiction. 2019;114(6):1060-73.
2. Blundell M, Dargan P, Wood D. The dark cloud of recreational drugs and vaping. QJM: An International Journal of Medicine. 2018;111(3):145-8.
3. Blundell M, Dargan P, Wood D. A cloud on the horizon–a survey into the use of electronic vaping devices for recreational drug and new psychoactive substance (NPS) administration. QJM: An International Journal of Medicine. 2018;111(1):9-14.
4. Breitbarth AK, Morgan J, Jones AL. E-cigarettes—an unintended illicit drug delivery system. Drug and alcohol dependence. 2018;192:98-111.
5. Abrams DI, Vizoso HP, Shade SB, Jay C, Kelly ME, Benowitz NL. Vaporization as a smokeless cannabis delivery system: a pilot study. Clinical Pharmacology & Therapeutics. 2007;82(5):572-8.
6. Roberts E, Copeland C, Robson D, McNeill A. Drug-related deaths associated with vaping product use in the United Kingdom. Addiction. 2021;n/a(n/a).
7. King BA, Jones CM, Baldwin GT, Briss PA. E-cigarette, or Vaping, Product Use-Associated Lung Injury: Looking Back, Moving Forward. Nicotine and Tobacco Research. 2020;22(Supplement_1):S96-S9.
8. McNeill A, Brose L, Calder R, Bauld L, Robson D. Vaping in England: an evidence update including mental health and pregnancy, March 2020: a report commissioned by Public Health England. London: Public Health England. 2020.
9. Hindocha C, Brose LS, Walsh H, Cheeseman H. Cannabis use and co-use in tobacco smokers and non-smokers: prevalence and associations with mental health in a cross-sectional, nationally representative sample of adults in Great Britain, 2020. Addiction. 2021;116(8):2209-19.
10. www.prolific.co.
11. Equality Act 2010 https://www.legislation.gov.uk/ukpga/2010/15/contents.
12. https://www.ons.gov.uk/census/censustransformationprogramme/census2021outputs/ukcensusdata.
13. Von Elm E, Altman DG, Egger M, Pocock SJ, Gøtzsche PC, Vandenbroucke JP. The Strengthening the Reporting of Observational Studies in Epidemiology (STROBE) statement: guidelines for reporting observational studies. Annals of internal medicine. 2007;147(8):573-7.
14. Williams M, Talbot P. Design features in multiple generations of electronic cigarette atomizers. International journal of environmental research and public health. 2019;16(16):2904.
15. https://www.ons.gov.uk/peoplepopulationandcommunity/healthandsocialcare/drugusealcoholandsmoking/.
16. Lim CC, Leung JK, Connor JP, Hall WD, Gartner C, Cheng BH, et al. Availability of substances for use in personal vaporisers on three online cryptomarkets. Drug and Alcohol Dependence. 2020;217:108254.

For peer review only

For peer review only

Online Supplementary Material

Contents

Figures

S1: The prevalence of ever vaping and current vaping of nicotine and non-nicotine drugs

Tables

S1: All non-nicotine drug types and subtypes asked about in the online cross-sectional survey

S2: Current use (within the last 30 days) profiles for all remaining non-nicotine drugs

S3: Sociodemographic breakdown of those participants who have never vaped, ever vaped any drug including nicotine and ever vaped any drug excluding nicotine

References

For peer review only

Figure S1: The prevalence of ever vaping and current vaping of nicotine and non-nicotine drugs

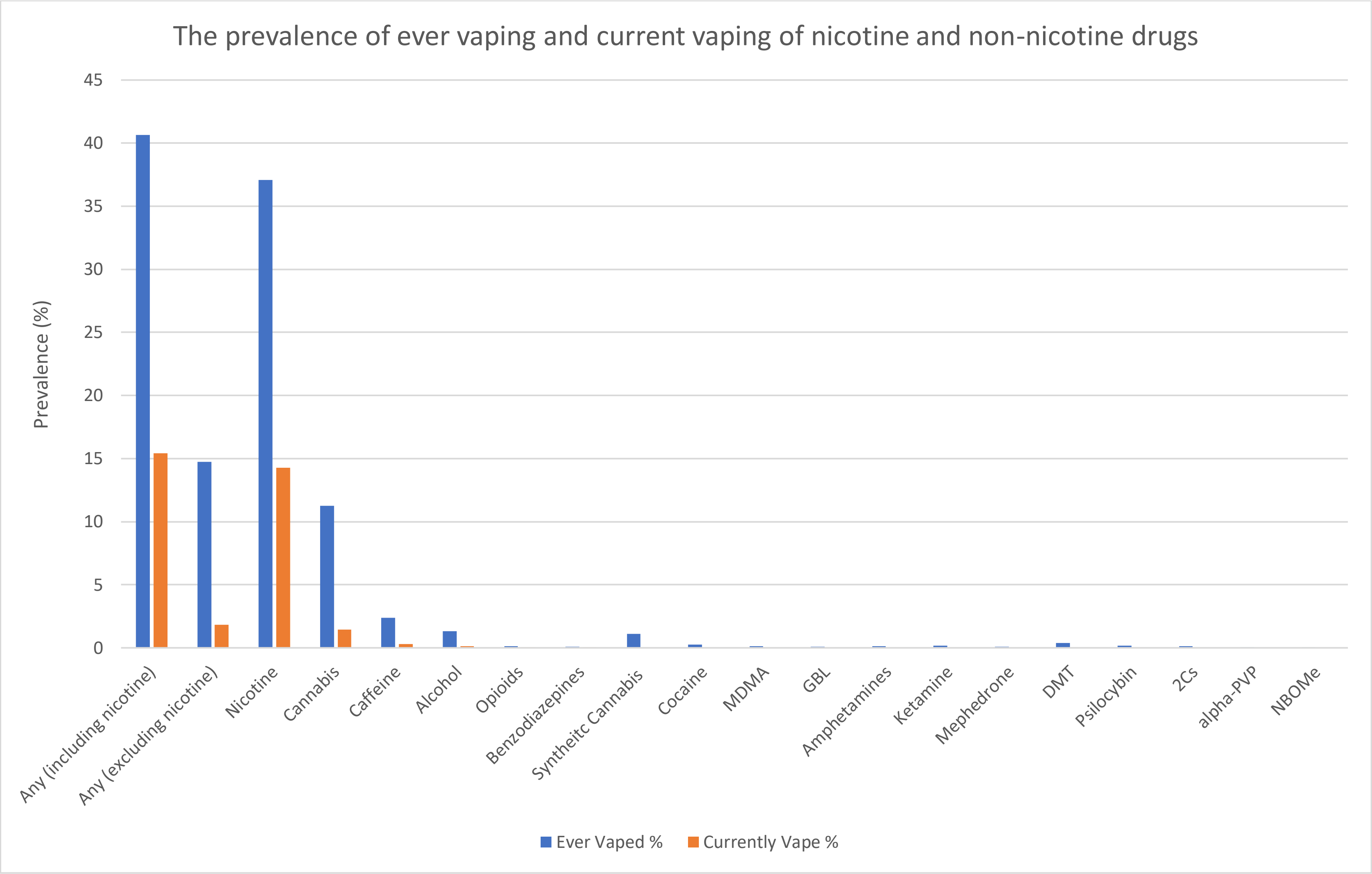

Tables

Table S1: All non-nicotine drug types and subtypes as they were detailed to participants in the online cross-sectional survey (adapted from Blundell et al. (1))

**Drug**	**Subtypes**
Nicotine	Nicotine e-liquid (freebase)<br>Nicotine salts
Caffeine	Caffeine
Alcohol	Alcohol liquid
Cannabis	Combined THC/CBD resin<br>THC resin<br>CBD resin<br>Combined THC/CBD concentrate<br>THC concentrate<br>CBD concentrate<br>Combined THC/CBD oil<br>THC oil<br>CBD oil<br>Butane hash oil<br>Dry herb/bud combined THC/CBD<br>Dry herb/bud THC<br>Dry herb/bud CBD
Synthetic Cannabis	Spice<br>Black mamba<br>Spice gold<br>Magic gold<br>Exodus<br>K2<br>Herbal incense<br>Spice arctic<br>Bonzai

For peer review only

Drug	Subtypes
	Annihilation<br>Thai high<br>AB-FUMINACA<br>ADB-PINACA<br>AM-2201<br>ADB-CHMICA<br>JWH-015<br>JWH-018<br>CP-47-497<br>5F-ADB<br>5F-AKB48<br>XLR-11<br>UR-144<br>HU-210<br>MDMB-CHMICA
Cocaine	Cocaine powder<br>Crack cocaine
MDMA	MDMA (aka Ecstasy Molly, Mandy)
GBL	GBL<br>GHB
Amphetamine	Ritalin (Methylphenidate)<br>Crystal methamphetamine (aka Ice, Tina, Crystal)
Opioids	Heroin<br>Morphine<br>Fentanyl
Ketamine	Ketamine (aka Special-K, Ket)
Mephedrone	Mephedrone (aka meow-meow, M-CAT)
DMT	DMT
Psilocybin	Psilocybin
Benzos	Diazepam (aka blues, Valium)<br>Clonazepam (aka Rivotril)<br>Alprazolam (aka Xanax)

For peer review only

Drug	Subtypes
2Cs	2CB<br>2CE
alpha-PVP	alpha-PVP aka gravelflakka
NBOMe	NBOMe (aka N-Bomb)
Other	Other, please describe

For peer review only

Table S2: Current use (within the last 30 days) profiles for all remaining non-nicotine drugs

	Ketamine	Synthetic Cannabis	Cocaine	Amphetamines	MDMA	GBL	Mephedrone	DMT	Psilocybin	2Cs	alpha-PVP	NBOMe
Currently use n (%)	1 (100%)	2 (100%)	2 (100%)	2 (100%)	0	0	0	0	0	0	0	0
Frequency of current use n (%)												
Daily	0 (0.0%)	0 (0.0%)	0 (0.0%)	1 (50.0%)	-	-	-	-	-	-	-	-
Weekly	0 (0.0%)	1 (50.0%)	1 (50.0%)	1 (50.0%)	-	-	-	-	-	-	-	-
Monthly	1 (100.0%)	1 (50.0%)	1 (50.0%)	0 (0.0%)	-	-	-	-	-	-	-	-
Currently use harmfully n (%)	0 (0.0%)	0 (0.0%)	1 (50.0%)	2 (100.0%)	-	-	-	-	-	-	-	-
Currently vape n (%)	0 (0.05)	0 (0.0%)	0 (0.0%)	0 (0.0%)	-	-	-	-	-	-	-	-
Frequency of current vaping n (%)												
Daily	-	-	-	-	-	-	-	-	-	-	-	-
Weekly	-	-	-	-	-	-	-	-	-	-	-	-
Monthly	-	-	-	-	-	-	-	-	-	-	-	-
Age first used mean (SE)	21.3 (1.38)	26.6 (1.37)	23.4 (2.14)	22.8 (3.12)	25.5 (5.17)	28 (2.74)	31.3 (4.81)	27.0 (2.51)	18.6 (1.02)	28.5 (4.14)	18.5 (3.40)	30 (N/A)
Age first vaped mean (SE)	24.4 (2.22)	27.0 (1.41)	31.3 (2.94)	25.8 (3.43)	26.5 (4.86)	30.5 (5.04)	31.7 (4.91)	27.2 (2.48)	27.4 (4.17)	35.7 (5.90)	18.5 (3.50)	30 (N/A)
t, P[1]	-1.9, 0.1	-2.3, 0.02	-2.7, 0.03	-1.4, 0.23	-2.2, 0.08	-1.0, 0.39	-1.0, 0.42	-1.0, 0.33	-1.9, 0.11	-1.1, 0.31	N/A	N/A
Modal reason for vaping initially	Because I thought I would enjoy it (42.8%)	Curiosity just wanted to try (31.1%)	Curiosity just wanted to try (30.0%)	Because I thought I would enjoy it (40.0%)	Because I thought I would enjoy it (50.0%)	Because I thought I would enjoy it (50.0%)	Curiosity just wanted to try (33.3%)	Curiosity just wanted to try (50.0%)	Curiosity just wanted to try (57.1%)	To avoid returning to other routes of administration (33.3%)	To quit other routes of administration (100.0%)	Don't know (100.0%)
Stated an intention to quit use n (%)	0 (0.0%)	0 (0.0%)	0 (0.0%)	1 (50.0%)	-	-	-	-	-	-	-	-

1 Compared using an unpaired t-test

For peer review only

Table S3: Sociodemographic breakdown of those participants who have never vaped, ever vaped any drug including nicotine and ever vaped any drug excluding nicotine

		All n (%)	Never vaped n (%)	Ever vaped any (including nicotine) n (%)	Ever vaped any (excluding nicotine) n (%)	Ever vaped any non-nicotine compared to never vapers (p)[1]
All	All	4027 (100%)	2,390 (100%)	1,637 (100%)	593 (100%)	-
Gender identity	Gender identity is the same as their sex registered at birth	3984 (98.9%)	2369 (99.1%)	1615 (98.7%)	580 (97.8%)	0.02
	Gender identity is not the same as their sex registered at birth	26 (0.7%)	12 (0.5%)	14 (0.8%)	9 (1.5%)	
	Do not wish to say	17 (0.4%)	9 (0.4%)	8 (0.5%)	4 (0.7%)	
Sexual orientation	Straight/Heterosexual	3548 (88.1%)	2176 (91.1%)	1372 (83.8%)	453 (76.4%)	<0.001
	Gay or Lesbian	153 (3.8%)	69 (2.9%)	84 (5.1%)	34 (5.7%)	
	Bisexual	237 (5.9%)	90 (3.8%)	147 (9.0%)	89 (15.0%)	
	Other sexual orientation	42 (1.0%)	26 (1.1%)	16 (1.0%)	9 (1.5%)	
	Do not wish to say	47 (1.2%)	29 (1.2%)	18 (1.1%)	8 (1.4%)	
Religion	No religion	2502 (62.1%)	1403 (58.7%)	1099 (67.1%)	424 (71.5%)	<0.001[2]
	Christian (including Church of England, Catholic, Protestant and all other Christian denominations)	1233 (30.6%)	820 (34.3%)	413 (25.2%)	112 (18.9%)	
	Buddhist	25 (0.6%)	12 (0.5%)	13 (0.8%)	6 (1.0%)	
	Hindu	34 (0.8%)	14 (0.6%)	20 (1.2%)	9 (1.5%)	
	Jewish	21 (0.5%)	13 (0.5%)	8 (0.5%)	4 (0.7%)	
	Muslim	113 (2.8%)	72 (3.0%)	41 (2.5%)	16 (2.7%)	
	Sikh	13 (0.3%)	9 (0.4%)	4 (0.2%)	1 (0.2%)	
	Any other religion	30 (0.7%)	17 (0.7%)	13 (0.8%)	10 (1.7%)	
	Do not wish to say	56 (1.4%)	30 (1.3%)	26 (1.6%)	11 (1.9%)	
Marital status	Never married and never registered in a civil partnership	1980 (49.2%)	1052 (44.0%)	928 (56.7%)	376 (63.4%)	<0.001[3]
	Married	1580 (39.2%)	1088 (45.5%)	492 (30.1%)	152 (25.6%)	
	In a registered civil partnership	56 (1.4%)	19 (0.8%)	37 (2.2%)	12 (2.0%)	
	Separated, but still legally married	51 (1.3%)	22 (0.9%)	29 (1.8%)	9 (1.5%)	
	Separated, but still legally in a civil partnership	3 (0.1%)	2 (0.1%)	1 (0.1%)	1 (0.2%)	
	Divorced	247 (6.1%)	147 (6.2%)	100 (6.1%)	30 (5.0%)	
	Formerly in a civil partnership which is now legally dissolved	4 (0.1%)	2 (0.1%)	2 (0.1%)	0 (0.0%)	
	Widowed	46 (1.1%)	30 (1.3%)	16 (1.0%)	2 (0.3%)	
	Surviving partner from a registered civil partnership	2 (0.1%)	1 (0.1%)	1 (0.1%)	0 (0.0%)	
	Do not wish to say	58 (1.4%)	27 (1.1%)	31 (1.9%)	11 (1.9%)	
Disability	Has a condition or illnesses which reduces their ability to carry out day-to-day activities	721 (17.9%)	358 (15.0%)	363 (22.2%)	163 (27.5%)	<0.001
	Does not have a condition or illnesses which reduces their ability to carry out day-to-day activities	3214 (79.8%)	1983 (83.0%)	1231 (75.2%)	413 (69.6%)	
	Do not wish to say	92 (2.3%)	49 (2.1%)	43 (2.6%)	17 (2.9%)	
Level of education	Has achieved a qualification at degree level or above	2506 (62.2%)	1526 (63.9%)	980 (59.9%)	393 (66.3%)	0.51
	Has not achieved a qualification at degree level or above	1489 (37.0%)	846 (35.4%)	643 (39.3%)	195 (32.9%)	
	Do not wish to say	32 (0.8%)	18 (0.8%)	14 (0.9%)	5 (0.8%)	

For peer review only

1 Compared using Fishers exact test; 2 For the purpose of statistical testing this category was collapsed into three levels: No religion, Christian (including Church of England, Catholic, Protestant and all other Christian denominations) and any other religion (Buddhist, Hindu, Jewish, Muslim, Sikh, Any other religion) 3 For the purpose of statistical testing this category was collapsed into three levels; Never married and never registered in a civil partnership, Married or in a registered civil partnership and any form of dissolution (Separated, but still legally married, Separated, but still legally in a civil partnership Divorced, Formerly in a civil partnership which is now legally dissolved, Widowed or Surviving partner from a registered civil partnership)

References

1. Blundell M, Dargan P, Wood D. A cloud on the horizon–a survey into the use of electronic vaping devices for recreational drug and new psychoactive substance (NPS) administration. QJM: An International Journal of Medicine. 2018;111(1):9-14.

For peer review only

STROBE Statement—Checklist of items that should be included in reports of ***cross-sectional studies***

	Item No	Recommendation	Page No
**Title and abstract**	1	(*a*) Indicate the study's design with a commonly used term in the title or the abstract	1
		(*b*) Provide in the abstract an informative and balanced summary of what was done and what was found	3
**Introduction**			
Background/rationale	2	Explain the scientific background and rationale for the investigation being reported	5
Objectives	3	State specific objectives, including any prespecified hypotheses	5
**Methods**			
Study design	4	Present key elements of study design early in the paper	6
Setting	5	Describe the setting, locations, and relevant dates, including periods of recruitment, exposure, follow-up, and data collection	6
Participants	6	(*a*) Give the eligibility criteria, and the sources and methods of selection of participants	6
Variables	7	Clearly define all outcomes, exposures, predictors, potential confounders, and effect modifiers. Give diagnostic criteria, if applicable	6
Data sources/ measurement	8*	For each variable of interest, give sources of data and details of methods of assessment (measurement). Describe comparability of assessment methods if there is more than one group	*6*
Bias	9	Describe any efforts to address potential sources of bias	6
Study size	10	Explain how the study size was arrived at	6/7
Quantitative variables	11	Explain how quantitative variables were handled in the analyses. If applicable, describe which groupings were chosen and why	6
Statistical methods	12	(*a*) Describe all statistical methods, including those used to control for confounding	6
		(*b*) Describe any methods used to examine subgroups and interactions	6
		(*c*) Explain how missing data were addressed	N/A
		(*d*) If applicable, describe analytical methods taking account of sampling strategy	6
		(*e*) Describe any sensitivity analyses	N/A
**Results**			
Participants	13*	(a) Report numbers of individuals at each stage of study—eg numbers potentially eligible, examined for eligibility, confirmed eligible, included in the study, completing follow-up, and analysed	7
		(b) Give reasons for non-participation at each stage	N/A
		(c) Consider use of a flow diagram	N/A
Descriptive data	14*	(a) Give characteristics of study participants (eg demographic, clinical, social) and information on exposures and potential confounders	7
		(b) Indicate number of participants with missing data for each variable of interest	N/A
Outcome data	15*	Report numbers of outcome events or summary measures	7
Main results	16	(*a*) Give unadjusted estimates and, if applicable, confounder-adjusted estimates and their precision (eg, 95% confidence interval). Make clear which confounders were adjusted for and why they were included	7

For peer review only - http://bmjopen.bmj.com/site/about/guidelines.xhtml

		(*b*) Report category boundaries when continuous variables were categorized	N/A
		(*c*) If relevant, consider translating estimates of relative risk into absolute risk for a meaningful time period	N/A
Other analyses	17	Report other analyses done—eg analyses of subgroups and interactions, and sensitivity analyses	7
**Discussion**			
Key results	18	Summarise key results with reference to study objectives	9
Limitations	19	Discuss limitations of the study, taking into account sources of potential bias or imprecision. Discuss both direction and magnitude of any potential bias	10
Interpretation	20	Give a cautious overall interpretation of results considering objectives, limitations, multiplicity of analyses, results from similar studies, and other relevant evidence	9/10
Generalisability	21	Discuss the generalisability (external validity) of the study results	9/10
**Other information**			
Funding	22	Give the source of funding and the role of the funders for the present study and, if applicable, for the original study on which the present article is based	2

*Give information separately for exposed and unexposed groups.

Note: An Explanation and Elaboration article discusses each checklist item and gives methodological background and published examples of transparent reporting. The STROBE checklist is best used in conjunction with this article (freely available on the Web sites of PLoS Medicine at http://www.plosmedicine.org/, Annals of Internal Medicine at http://www.annals.org/, and Epidemiology at http://www.epidem.com/). Information on the STROBE Initiative is available at www.strobe-statement.org.

For peer review only

For peer review only - http://bmjopen.bmj.com/site/about/guidelines.xhtml

BMJ Open

The pattern and prevalence of vaping nicotine and non-nicotine drugs in the United Kingdom: A cross-sectional study

Journal:	*BMJ Open*
Manuscript ID	bmjopen-2022-066826.R1
Article Type:	Original research
Date Submitted by the Author:	02-Feb-2023
Complete List of Authors:	Roberts , Emmert; King's College London, National Addiction Centre; South London and Maudsley NHS Foundation Trust,<br>Taylor, Eve; King's College London<br>Cox, Sharon; University College London, Behavioural Science and Health; SPECTRUM Research Consortium,<br>Brose, Leonie; King's College London, Addictions Department<br>McNeill, Ann; King’s College London, UK Centre for Tobacco Control Studies, National Addiction Centre, Institute of Psychiatry<br>Robson, Deborah; Kings College London, Psychiatry
**Primary Subject Heading**:	Addiction
Secondary Subject Heading:	Epidemiology, Smoking and tobacco
Keywords:	EPIDEMIOLOGY, Substance misuse < PSYCHIATRY, PUBLIC HEALTH

SCHOLARONE™
Manuscripts

BMJ

I, the Submitting Author has the right to grant and does grant on behalf of all authors of the Work (as defined in the below author licence), an exclusive licence and/or a non-exclusive licence for contributions from authors who are: i) UK Crown employees; ii) where BMJ has agreed a CC-BY licence shall apply, and/or iii) in accordance with the terms applicable for US Federal Government officers or employees acting as part of their official duties; on a worldwide, perpetual, irrevocable, royalty-free basis to BMJ Publishing Group Ltd ("BMJ") its licensees and where the relevant Journal is co-owned by BMJ to the co-owners of the Journal, to publish the Work in this journal and any other BMJ products and to exploit all rights, as set out in our licence.

The Submitting Author accepts and understands that any supply made under these terms is made by BMJ to the Submitting Author unless you are acting as an employee on behalf of your employer or a postgraduate student of an affiliated institution which is paying any applicable article publishing charge ("APC") for Open Access articles. Where the Submitting Author wishes to make the Work available on an Open Access basis (and intends to pay the relevant APC), the terms of reuse of such Open Access shall be governed by a Creative Commons licence – details of these licences and which Creative Commons licence will apply to this Work are set out in our licence referred to above.

Other than as permitted in any relevant BMJ Author's Self Archiving Policies, I confirm this Work has not been accepted for publication elsewhere, is not being considered for publication elsewhere and does not duplicate material already published. I confirm all authors consent to publication of this Work and authorise the granting of this licence.

For peer review only

Title: The pattern and prevalence of vaping nicotine and non-nicotine drugs in the United Kingdom: A cross-sectional study

Authors:

Dr Emmert Roberts, National Addiction Centre, Institute of Psychiatry, Psychology and Neuroscience, King's College London, 4 Windsor Walk, London, SE5 8AF, United Kingdom and the South London and the Maudsley NHS Foundation Trust, Denmark Hill, London, United Kingdom SE5 8AZ

Eve Taylor, National Addiction Centre, Psychology and Neuroscience, King's College London, 4 Windsor Walk, London, SE5 8AF, United Kingdom

Dr Sharon Cox, Institute of Epidemiology & Health, Faculty of Population Health Sciences, University College London, 30 Guilford Street London WC1N 1EH

Dr Leonie Brose, National Addiction Centre, Institute of Psychiatry, Psychology and Neuroscience, King's College London, United Kingdom 4 Windsor Walk, London, SE5 8AF, United Kingdom

Professor Ann McNeill, National Addiction Centre, Institute of Psychiatry, Psychology and Neuroscience, King's College London, 4 Windsor Walk, London, SE5 8AF, United Kingdom

Dr Debbie Robson, National Addiction Centre, Institute of Psychiatry, Psychology and Neuroscience, King's College London, 4 Windsor Walk, London, SE5 8AF, United Kingdom

Corresponding author:

Dr Emmert Roberts
National Addiction Centre, Psychology and Neuroscience, King's College London, 4 Windsor Walk, London, SE5 8AF, United Kingdom
+447754156145
emmert.roberts@kcl.ac.uk

Running Head:

Vaping nicotine and non-nicotine drugs in UK

For peer review only

Word Count: 2431

For peer review only

For peer review only - http://bmjopen.bmj.com/site/about/guidelines.xhtml

Abstract (Word Count: 298) (Max 300)

Objectives: Electronic vaping devices are being used to consume nicotine and non-nicotine psychoactive drugs. We aimed to determine the pattern and prevalence of using vaping devices for nicotine and/or non-nicotine drug administration in the United Kingdom and how these differ by drug type and individual sociodemographic characteristics. We explored reasons for vaping onset and continuation.

Design: An online cross-sectional survey

Participants: A convenience sample of adults (aged ≥18 years) in the UK

Primary and Secondary Outcome Measures: The primary outcome was prevalence of current use (within the last 30 days) of a vaping device to administer either nicotine or eighteen types of non-nicotine drug. We additionally evaluated reasons for onset and continuation of vaping. Sociodemographic characteristics were compared between the UK general population using census data and those vaping non-nicotine drugs.

Results: We recruited 4027 participants of whom 1637 (40.7%) had ever used an electronic vaping device; 1495 (37.1%) had ever vaped nicotine and 593 (14.7%) had ever vaped a non-nicotine drug. Overall, 574 (14.3%) currently vaped nicotine and 74 (1.8%) currently vaped a non-nicotine drug. The most common currently vaped non-nicotine drug was cannabis (n=58, 1.4%). For nicotine, people's modal reasons to start and continue vaping was to quit smoking tobacco. For almost all other drugs people's modal reason to start vaping was curiosity and to continue was enjoyment. Compared to the general population, the population who had ever vaped a non-nicotine drug were significantly younger, had more disabilities and fewer identified as white, female, heterosexual or religious.

Conclusions: A non-trivial number of people report current use and ever use of an electronic vaping device for non-nicotine drug administration. As vaping technology advances and drug consumption changes, understanding patterns of use and associated behaviours is likely to be increasingly important to both users and healthcare professionals.

For peer review only - http://bmjopen.bmj.com/site/about/guidelines.xhtml

Strengths and limitations of this study

- The study reports, to our knowledge, the largest cross-sectional adult sample of the sociodemographic profile of people using electronic vaping devices to consume non-nicotine drugs, alongside people's reasons for onset and continuation of vaping, and the type of devices they use.
- The study relied on self-report to identify non-nicotine drug use and may be subject to social desirability bias or participant misinterpretation.
- The study was conducted using an online convenience sample and several populations who have historically higher rates of non-nicotine drug consumption may have been excluded due to its digital nature or other life circumstances (e.g., people experiencing homelessness or currently serving a prison sentence).

For peer review only

Introduction

In the last decade the advent of electronic nicotine vaping products has changed how some people use nicotine and recent reports also confirm use of electronic vaping devices to administer non-nicotine psychoactive drugs. (1-4) As innovation in technology develops, the prevalence and patterns of using electronic vaping devices to facilitate non-nicotine drug use are subject to change. (5) Whilst in some circumstances vaping non-nicotine drugs may present fewer harms when compared to other routes of administration (e.g., smoking), in some cases vaping could potentially pose unique harms due to the speed of onset of drug effects, alongside the concomitant use of any adulterants. (5-7) Most notably the 2019 United States outbreak of acute lung injury associated with vaping illicit cannabinoid products contaminated with vitamin E acetate highlights the potential for associated harms. (8)

Monitoring of nicotine vaping in the United Kingdom is well documented, (9) whilst data on vaping of non-nicotine drugs is sparse. (10-12) Understanding the prevalence and patterns of vaping non-nicotine drugs is important for healthcare professionals to both facilitate discussions regarding consumers' preferred routes of drug administration, and to provide appropriate harm reduction advice. Whilst a recent review of online drug user forums has identified over 300 reports related to the manufacture and/or use of e-liquids containing non-nicotine drugs, (2) few epidemiological studies have been conducted to determine the prevalence and patterns of using electronic vaping devices to administer different non-nicotine drugs. Although some studies have examined vaping in the context of nicotine and cannabis administration, (10-12) to our knowledge, only a single cross-sectional study conducted in 2017 in adults in the United Kingdom (UK) reported that 340 participants (13.6%) had ever used an electronic vaping device for any type of recreational drug administration during their lifetime. (3) However, this estimate has not been reproduced, the sociodemographic characteristics of the cohort of people vaping non-nicotine drugs have not been fully evaluated and there has been little examination of why people use vaping as a route of non-nicotine drug administration.

To address this gap, we aimed to determine the pattern and prevalence of using electronic vaping devices for nicotine and non-nicotine drug administration in the UK and how these differ by drug type and individual sociodemographic characteristics. In addition, we aimed to examine individual reasons for the onset of vaping non-nicotine drugs and why people continue vaping as a route of administration.

For peer review only - http://bmjopen.bmj.com/site/about/guidelines.xhtml

Methods

An anonymous voluntary cross-sectional survey recruiting a convenience sample of adult participants (aged ≥18 years) through the online Prolific platform. (13) Prolific, using their survey panel of research respondents based in the UK, advertised the study to all adults on their platform. (14) The study protocol, questionnaire and statistical analysis plan were pre-registered on the Open Science Framework (OSF) and are available at https://osf.io/bdfhp/. The survey went live on March 10th 2022 with the pre-specified minimum sample size of 3,275 being reached within twenty-four hours.

The survey used sociodemographic questions identical to those within the UK census and questions relating to vaping were based on the previous survey by Blundell et al. and adapted by the research team to include questions relating to additional substances not previously captured, vaping device type, and reasons behind vaping. (3, 15) The final published questionnaire is additionally available in the online supplementary material. Participants were required only to complete sections of the questionnaire applicable to their self-described vaping status of each substance and answers forced prior to moving onto the subsequent section thus no missing data could be generated.

The primary outcome was the prevalence of current use of a vaping device to administer either nicotine or eighteen specific types of non-nicotine drugs, defined as use in the past 30 days (A complete list of all non-nicotine drug types and subtypes studied can be found in the online supplementary material as table S1). Secondary outcomes included prevalence of ever using a vaping device to administer either nicotine or non-nicotine drugs and current use of nicotine or non-nicotine drugs by routes of administration other than vaping. We additionally collected if participants considered any currently consumed drug was having a negative consequence on their mental or physical health (harmful use) and on frequency of current use and vaping, age of first use and vaping onset, reasons for onset of vaping and why people continue vaping as a route of administration.

Individual UK census defined sociodemographic characteristics were also collected including level of educational attainment and eight 'protected' characteristics as defined by the UK Equality Act (age, sex, disability, gender identity, sexual orientation, ethnicity, religion and marital status). (16)

Sociodemographic and vaping characteristics were compared between those that had used an electronic vaping device to vape a non-nicotine drug and those who had never used an

For peer review only - http://bmjopen.bmj.com/site/about/guidelines.xhtml

electronic vaping device. Additionally, study sample sociodemographic characteristics were compared with the UK general population using publicly available data from the UK census. (17) Continuous variables were compared using an unpaired t-test, and categorical variables using Fisher's exact test, all comparisons were bivariate. Where there were more than two levels to a categorical variable, adjusted residuals, where the significance level of $\alpha = 0.01$, were calculated to identify which individual cells contributed to between group differences. (18) All analyses were conducted using STATA SE version 15.1 with the significance level set at 0.05. The study was reported in accordance with the STrengthening the Reporting of OBservational studies in Epidemiology STROBE statement for observational studies. (19)

Patient and Public Involvement

No patient or public involvement.

For peer review only

For peer review only - http://bmjopen.bmj.com/site/about/guidelines.xhtml

Results

There were a total of 4027 participants of whom 1637 (40.7%) had ever used an electronic vaping device; 1495 people (37.1%) had ever vaped nicotine and 593 (14.7%) had ever vaped a non-nicotine drug. 1044 (25.9%) had only ever exclusively vaped nicotine, 142 (3.5%) had only ever exclusively vaped a non-nicotine drug and 451 (11.2%) had ever vaped both nicotine and a non-nicotine drug. The majority of the overall sample were aged between 25 and 44 (57.9%), predominantly identified as female (64.9%), and predominantly identified as being from a white ethnicity (80.8%). (Table 1)

For peer review only

For peer review only - http://bmjopen.bmj.com/site/about/guidelines.xhtml

Table 1: The age, sex and ethnicity breakdown of those participants who have never vaped, ever vaped any drug including nicotine and ever vaped any non-nicotine drug

Sociodemographic characteristic		All n (%)	Never vaped n (%)	Ever vaped any drug (including nicotine) n (%)	Ever vaped any non-nicotine drug n (%)	Ever vaped any non-nicotine drug compared to never vaped (p)[1]	Ever vaped any non-nicotine drug compared to UK general population (p)[1]
All	All	4027 (100%)	2390 (100%)	1637 (100%)	593 (100%)	-	-
Age	18-24	441 (11.0%)	199 (8.3%)	242 (14.8%)	112 (18.9%)	<0.001[2]	<0.001[2]
	25-34	1265 (31.4%)	670 (28.0%)	595 (36.4%)	244 (41.2%)		
	35-44	1067 (26.5%)	632 (26.4%)	435 (26.6%)	146 (24.6%)		
	45-54	629 (15.6%)	410 (17.2%)	219 (13.4%)	57 (9.6%)		
	55-64	428 (10.6%)	319 (12.4%)	109 (6.7%)	25 (4.2%)		
	65-74	171 (4.3%)	138 (5.8%)	33 (2.0%)	8 (1.4%)		
	over 75	26 (0.7%)	22 (0.9%)	4 (0.2%)	1 (0.2%)		
Sex	Male	1400 (34.8%)	754 (31.2%)	655 (40.1%)	271 (45.7%)	<0.001[3]	<0.001[3]
	Female	2614 (64.9%)	1641 (68.7%)	973 (59.4%)	317 (53.5%)		
	Do not wish to say	13 (0.3%)	4 (0.2%)	9 (0.6%)	5 (0.8%)		
Ethnicity	English/Welsh/Scottish/ Northern Irish/British	3254 (80.8%)	1952 (81.7%)	1302 (79.5%)	437 (73.7%)	<0.001[4]	<0.001[4]
	Irish	42 (1.0%)	27 (1.1%)	15 (0.9%)	9 (1.5%)		
	Gypsy or Irish Traveller	2 (0.1%)	0 (0.0%)	2 (0.1%)	1 (0.2%)		
	Any other White background	227 (5.6%)	128 (5.4%)	99 (6.1%)	42 (7.1%)		
	White and Black Caribbean	37 (0.9%)	17 (0.7%)	20 (1.2%)	7 (1.2%)		
	White and Black African	14 (0.4%)	8 (0.3%)	6 (0.4%)	4 (0.7%)		
	White and Asian	38 (0.9%)	14 (0.6%)	24 (1.5%)	15 (2.5%)		
	Any other Mixed/ Multiple ethnic background	21 (0.5%)	12 (0.5%)	9 (0.6%)	4 (0.7%)		
	Indian	78 (1.9%)	42 (1.7%)	36 (2.2%)	16 (2.7%)		
	Pakistani	52 (1.3%)	27 (1.1%)	25 (1.5%)	11 (1.9%)		
	Bangladeshi	17 (0.4%)	12 (0.5%)	5 (0.3%)	2 (0.3%)		
	Chinese	58 (1.4%)	37 (1.6%)	21 (1.3%)	12 (2.0%)		
	Any other Asian background	24 (0.6%)	13 (0.5%)	11 (0.7%)	5 (0.8%)		
	African	69 (1.7%)	52 (2.2%)	17 (1.0%)	7 (1.2%)		
	Caribbean	29 (0.7%)	18 (0.8%)	11 (0.7%)	3 (0.5%)		
	Any other Black/African/ Caribbean background	5 (0.1%)	1 (0.1%)	4 (0.2%)	2 (0.3%)		
	Arab	14 (0.4%)	7 (0.3%)	7 (0.4%)	4 (0.7%)		
	Any other ethnic group	21 (0.5%)	8 (0.3%)	13 (0.8%)	7 (1.2%)		
	Do not wish to say	25 (0.6%)	15 (0.6%)	10 (0.6%)	5 (0.8%)		

[1] Compared using Fishers exact test; [2] For the purpose of statistical testing this category was collapsed into four levels: 18-24, 25-34, 35-44 and over 45; [3] For the purpose of statistical testing this category was collapsed into two levels: Male and Female; [4] For the purpose of statistical testing this category was collapsed into two levels: white (English/Welsh/Scottish/Northern Irish/British, Irish, Gypsy or Irish Traveller or any other White background) and non-white (White and Black Caribbean, White and Black African, White and Asian, Any other Mixed/Multiple ethnic background, Indian, Pakistani, Bangladeshi, Chinese, Any other Asian background, African, Caribbean, Any other Black/African/Caribbean background, Arab or any other ethnic group)

For peer review only

For peer review only - http://bmjopen.bmj.com/site/about/guidelines.xhtml

About a fifth (n=834, 20.7%) were currently using a nicotine product; 595 (14.8%) were currently smoking tobacco, 574 (14.3%) were currently vaping nicotine and 280 (7.0%) were currently concurrently smoking tobacco and vaping nicotine. Fewer (n=217, 5.4%) participants were currently using a non-nicotine drug, of whom 74 (1.8%) were currently vaping a non-nicotine drug. The only non-nicotine drugs currently being vaped were cannabis 58 (1.4%), caffeine 12 (0.3%), alcohol 5 (0.1%), opioids 1 (0.02%) and benzodiazepines 1 (0.02%).

A profile of current use for those drugs currently being vaped is available in table 2 with a breakdown for all other drugs available in the online supplementary material as table S2. Participants' mean age at first use by any route was statistically significantly lower than their mean age at first vaping for nicotine, alcohol, caffeine, cannabis, synthetic cannabinoids and cocaine. For nicotine, people's modal reason to try vaping and their current reason to vape was to quit smoking tobacco. For almost all other drugs, people's modal reason to try vaping was curiosity and their modal reason to currently vape was because of enjoyment. The modal type of electronic vaping device currently used was a commercially bought electronic or e-cigarette for all studied drugs except cannabis for which the model type of device currently used was a commercially bought electronic device to vaporise dry herbs.

For peer review only - http://bmjopen.bmj.com/site/about/guidelines.xhtml

Table 2: Current use profiles for all drugs which participants were currently vaping (within the last 30 days)

	Any (including nicotine)	Any non-nicotine drug	Nicotine	Caffeine	Alcohol	Cannabis	Opioids	Benzodiazepines
Currently use n (%)	929 (100%)	217 (100%)	834 (100%)	45 (100%)	29 (100%)	146 (100%)	2 (100%)	1 (100%)
Frequency of current use n (%)								
Daily	-	-	601 (72.1%)	28 (62.2%)	4 (13.8%)	58 (39.7%)	1 (50.0%)	0 (0.0%)
Weekly	-	-	118 (14.2%)	16 (35.6%)	14 (48.3%)	45 (30.8%)	1 (50.0%)	0 (0.0%)
Monthly	-	-	115 (13.8%)	1 (2.2%)	11 (37.9%)	43 (29.5%)	0 (0.0%)	1 (100.0%)
Currently self-report harmful use n (%)	542 (58.3%)	73 (33.6%)	508 (60.9%)	14 (31.1%)	13 (44.8%)	47 (32.2%)	1 (50.0%)	0 (0.0%)
Currently vape n (%)	621 (68.8%)	74 (34.1%)	574 (68.8%)	12 (26.7%)	5 (17.2%)	58 (39.7%)	1 (50.0%)	1 (100.0%)
Frequency of current vaping n (%)								
Daily	-	-	363 (43.5%)	7 (15.6%)	0 (0.0%)	14 (9.6%)	0 (0.0%)	0 (0.0%)
Weekly	-	-	130 (15.6%)	4 (8.9%)	2 (6.9%)	22 (15.1%)	1 (50.0%)	1 (100.0%)
Monthly	-	-	81 (9.7%)	1 (2.2%)	3 (10.3%)	22 (15.1%)	0 (0.0%)	0 (0.0%)
Age first used mean (SE)	17.2 (0.15)	19.8 (0.36)	17.1 (0.15)	22.3 (1.24)	18.3 (1.09)	19.1 (0.34)	24.2 (5.40)	25.0 (4.56)
Age first vaped mean (SE)	29.8 (0.28)	27.9 (0.42)	30.2 (0.30)	27.6 (1.83)	24.6 (1.4)	28.1 (0.46)	35.3 (5.90)	29.8 (7.86)
t, p[1]	-41.5, <0.001	-20.3, <0.001	-41.2, <0.001	-6.8, <0.001	-4.3, <0.001	-19.7, <0.001	-2.2, 0.08	-1.3, 0.27
Modal reason for vaping initially	-	-	To quit smoking tobacco (31.7%)	Curiosity just wanted to try (37.5%)	Curiosity just wanted to try (50.9%)	Curiosity just wanted to try (41.6%)	Curiosity just wanted to try (50.0%)	Curiosity just wanted to try (25.0%)
Modal reason for vaping currently	-	-	To quit smoking tobacco (23.0%)	Because I enjoy it (41.7%)	Because I enjoy it (60.0%)	Because I enjoy it (34.5%)	For some other reason (100.0%)	Because I enjoy it (100.0%)
Stated an intention to quit n (%)	567 (61.0%)	53 (24.4%)	541 (64.9%)	14 (31.1%)	9 (31.0%)	29 (19.9%)	1 (50.0%)	1 (100%)
Modal type of electronic vaping device currently used (%)	-	-	Commercially bought e-cigarette (86.4%)	Commercially bought e-cigarette (75.0%)	Commercially bought e-cigarette (40.0%)	Commercially bought electronic device used to vaporise dry herbs (53.5%)	Commercially bought e-cigarette (100.0%)	Commercially bought e-cigarette (100.0%)

[1] Compared using an unpaired t-test

For peer review only - http://bmjopen.bmj.com/site/about/guidelines.xhtml

Of the 593 people who had ever vaped a non-nicotine drug, 484 (12.0% of the overall sample) had only ever vaped one type of non-nicotine drug and 109 (2.7%) had vaped two or more types of non-nicotine drugs. The most frequently ever vaped non-nicotine drug was cannabis (n=454, 11.3%), of whom 129 (3.2%) people had only ever vaped cannabidiol (CBD) products. This was followed by caffeine (n=96, 2.4%), alcohol (n=53, 1.3%), synthetic cannabinoids (n=45, 1.1%), Dimethyltryptamine (DMT) (n=16, 0.4%), cocaine (n=10, 0.2%), psilocybin (n=7, 0.2%), ketamine (n=7, 0.2%), opioids (n=6, 0.1%), MDMA (n=6, 0.1%), 2Cs (n=6, 0.1%), amphetamines (n=5, 0.1%), Gamma-butyrolactone (GBL) (n=4, 0.1%), benzodiazepines (n=4, 0.1%), mephedrone (n=3, 0.1%), alpha-PVP (n=2, 0.1%) and NBOMe (n=1, 0.02%). A bar chart depicting the prevalence of ever and current vaping for each drug is available in the online supplementary material as figure S1.

When comparing the study participants who had ever vaped a non-nicotine drug to both the UK general population and to those study participants who had never vaped, they were significantly different in every studied protected characteristic sociodemographic category. This group was significantly younger (with significantly more people aged <35 and significantly fewer aged >45), had a higher proportion of males, there were fewer people identifying with white ethnicities, there were more individuals who identified as LGBT+, more non-religious individuals, more people with disabilities, and fewer married people. However, there was no statistically significant difference by level of educational attainment. A breakdown by age, sex and ethnicity can be found in table 1, with the remaining sociodemographic differences available in the online supplementary material as table S3.

For peer review only

Discussion

In this study of 4027 respondents, 1.8% reported they were currently vaping a non-nicotine drug and 14.7% reported ever having vaped a non-nicotine drug. About one in six respondents were currently vaping nicotine around half of whom were also smoking tobacco.

Current prevalence of smoking tobacco among participants in our study (14.8%) was similar to those from other surveys in 2022 in England, such as estimates from the Smoking Toolkit Study (STS) (15.0%) and the Action on Smoking and Health (ASH) adult survey (13.2%). (20, 21) However, our estimate of current vaping was higher, at 14.5%, than estimates from the STS survey (9.3%) and ASH-Adult survey (8.3%). (20, 21) This is possibly due to sampling differences or how prevalence was estimated, with our survey including all adults who have vaped any substance in the past 30 days as current vapers, therefore likely capturing occasional/social/one-off use among people who would not identify themselves as 'vapers'. Additionally, the day prior to data collection was national 'No Smoking Day' in the UK which may have affected estimates.

The prevalence of ever using a vaping device to administer a non-nicotine drug was similar to estimates from a previous study (14.7% vs. 13.6%), (3) however prevalence estimates of currently vaping a non-nicotine drug were substantially lower (1.8% vs. 9.4%). (3, 10) This could be explained by sampling differences or may perhaps demonstrate that current prevalence may fluctuate dependent on technological or other changes in the vaping device market with new products potentially leading to curiosity-driven experimentation that may not be sustained over time. (22) Temporal prevalence trends are likely to reveal more about these relationships than isolated cross-sectional sampling.

When compared to the general population, the non-nicotine drug vaping population were significantly different across all studied protected characteristics, however their sociodemographic profile was broadly comparable to those of non-nicotine drug users in publicly available UK cross-sectional data, e.g., the Crime Survey for England and Wales. (23) Participants' mean age at first use by any route was consistently lower than respondents mean age at first vaping for all studied drugs, although lack of statistical power may account for the fact this was only significant for a few substances. This suggests that vaping is generally a rare first route of drug administration.

Similar to other studies, cannabis was the most commonly vaped non-nicotine drug with 11.3% of total respondents having ever vaped a cannabis product, however just over a

For peer review only - http://bmjopen.bmj.com/site/about/guidelines.xhtml

quarter of respondents had only ever vaped a product containing CBD which, at the time of the survey, was legal to purchase and consume in the UK. (3, 24) This underscores vaping should routinely be enquired about as a potential method of administration among cannabis users, and any future work on understanding relative cannabis-related harms should include vaping when considering routes of administration. Concerningly 45 individuals reported vaping synthetic cannabinoids, which have been linked to two vaping product related deaths in the UK. (7)

Whilst the study is, to our knowledge, the largest cross-sectional sample in the UK published literature of people ever vaping a range of non-nicotine drugs there are several limitations. The study relied on self-report to identify drug use and may therefore be subject to social desirability bias and was conducted using an online convenience sample. Several populations who have historically higher rates of non-nicotine drug consumption may have been excluded due to the study's digital nature or other life circumstances (e.g., people experiencing homelessness or currently serving a prison sentence). This is particularly important given the harms associated with drug use are disproportionately higher within these populations. (7) The small numbers of people vaping specific substances prevent robust statistical analyses due to small cell sizes and as such statistical testing should be interpreted with caution. Future studies with larger sample sizes or meta-analyses may be able to overcome these limitations. Additionally, despite the survey questions stating that flavoured e-liquids such as coffee and alcohol flavours do not contain either caffeine or alcohol, we cannot be sure that respondents did not misinterpret these questions and thus reported vaping prevalence results for alcohol and caffeine may represent overestimates.

Understanding people's experiences and perceptions of vaping as a means of administering non-nicotine drugs is likely to be increasingly important both to users and to healthcare and policy professionals. This study demonstrates that a non-trivial number of people have ever consumed non-nicotine drugs using vaping devices and understanding how this prevalence changes over time and which individuals may be most at risk of harm or benefit from this administration method requires further exploration. Future research should be conducted in larger non-UK samples, examine any potential trends in use of vaping products to administer non-nicotine drugs and examine vaping in samples known to experience disproportionate drug-related harms.

For peer review only

Ethics Approval

Informed consent was obtained from all participants and ethical approval gained from the King's College London Health Faculties Research Ethics Subcommittee (Reference: HR-21/22-26249).

Contributorship statement:

ER: Conceptualization; Methodology; Investigation; Data curation; Formal analysis; Project administration; Supervision; Funding acquisition; Writing - original draft.
ET: Methodology; Investigation; Data curation; Formal analysis; Project administration; Supervision; Writing - review & editing.
SC: Investigation; Writing - review & editing.
LB: Investigation; Writing - review & editing.
AM: Conceptualization; Supervision; Writing - review & editing.
DR: Conceptualization; Methodology; Investigation; Supervision; Funding acquisition; Writing - review & editing.

Funding:

This study was supported by the National Institute of Health Research (NIHR) Biomedical Research Centre at South London and Maudsley NHS Foundation Trust and King's College London. ER is funded by a Commonwealth Fund Harkness Fellowship in Healthcare Policy and Practice. SC receives salary support from Cancer Research UK (PRCRPG-Nov21\100002). ET, DR and AM are funded by the National Institute for Health and Care Research (NIHR) Health Protection Research Unit in Environmental Exposures and Health, a partnership between the UK Health Security Agency and Imperial College London. DR and AM are also funded by the National Institute for Health Research (NIHR) Applied Research Collaboration (ARC) South London at King's College Hospital NHS Foundation Trust. AM, DR and LB are members of SPECTRUM, a UK Prevention Research Partnership Consortium. UKPRP is an initiative funded by the UK Research and Innovation Councils, the Department of Health and Social Care (England) and the UK devolved administrations, and leading health research charities. AM is a National Institute for Health and Care Research (NIHR) Senior Investigator. The funders had no role in: study design, in the collection, analysis, or interpretation of data; in the writing of the report; or in the decision to submit the paper for publication. The views expressed in this article are those of the authors and not

For peer review only - http://bmjopen.bmj.com/site/about/guidelines.xhtml

necessarily those of the Commonwealth Fund, NIHR, or the Department of Health and Social Care.

Competing Interests:

All authors have completed the ICJME Unified Competing Interest form (available on request from the corresponding author) and declare: None

Data sharing statement:

The study protocol, questionnaire and statistical analysis plan are available at https://osf.io/bdfhp/.

For peer review only

References

1. Gravely S, Driezen P, Ouimet J, Quah AC, Cummings KM, Thompson ME, et al. Prevalence of awareness, ever-use and current use of nicotine vaping products (NVPs) among adult current smokers and ex-smokers in 14 countries with differing regulations on sales and marketing of NVPs: cross-sectional findings from the ITC Project. Addiction. 2019;114(6):1060-73.
2. Blundell M, Dargan P, Wood D. The dark cloud of recreational drugs and vaping. QJM: An International Journal of Medicine. 2018;111(3):145-8.
3. Blundell M, Dargan P, Wood D. A cloud on the horizon–a survey into the use of electronic vaping devices for recreational drug and new psychoactive substance (NPS) administration. QJM: An International Journal of Medicine. 2018;111(1):9-14.
4. Keyes KM, Kreski NT, Ankrum H, Cerdá M, Chen Q, Hasin DS, et al. Frequency of adolescent cannabis smoking and vaping in the United States: Trends, disparities and concurrent substance use, 2017–19. Addiction. 2022.
5. Breitbarth AK, Morgan J, Jones AL. E-cigarettes—an unintended illicit drug delivery system. Drug and alcohol dependence. 2018;192:98-111.
6. Abrams DI, Vizoso HP, Shade SB, Jay C, Kelly ME, Benowitz NL. Vaporization as a smokeless cannabis delivery system: a pilot study. Clinical Pharmacology & Therapeutics. 2007;82(5):572-8.
7. Roberts E, Copeland C, Robson D, McNeill A. Drug-related deaths associated with vaping product use in the United Kingdom. Addiction. 2021;n/a(n/a).
8. King BA, Jones CM, Baldwin GT, Briss PA. E-cigarette, or Vaping, Product Use-Associated Lung Injury: Looking Back, Moving Forward. Nicotine and Tobacco Research. 2020;22(Supplement_1):S96-S9.
9. McNeill A, Brose L, Calder R, Bauld L, Robson D. Vaping in England: an evidence update including mental health and pregnancy, March 2020: a report commissioned by Public Health England. London: Public Health England. 2020.
10. Hindocha C, Brose LS, Walsh H, Cheeseman H. Cannabis use and co-use in tobacco smokers and non-smokers: prevalence and associations with mental health in a cross-sectional, nationally representative sample of adults in Great Britain, 2020. Addiction. 2021;116(8):2209-19.
11. Fataar F, Hammond D. The prevalence of vaping and smoking as modes of delivery for nicotine and cannabis among youth in Canada, England and the United States. International journal of environmental research and public health. 2019;16(21):4111.
12. Sund LJ, Wood DM, Archer JR, Blundell MS, Dargan PI. The unseen cloud: a survey of vaping practices and the acquisition of vaping products within the UK. QJM: An International Journal of Medicine. 2022.
13. www.prolific.co.
14. Newman A, Bavik YL, Mount M, Shao B. Data collection via online platforms: Challenges and recommendations for future research. Applied Psychology. 2021;70(3):1380-402.
15. Cox S, West R, Notley C, Soar K, Hastings J. Toward an ontology of tobacco, nicotine and vaping products. Wiley Online Library; 2022.
16. Equality Act 2010 https://www.legislation.gov.uk/ukpga/2010/15/contents.

For peer review only - http://bmjopen.bmj.com/site/about/guidelines.xhtml

17. https://www.ons.gov.uk/census/censustransformationprogramme/census2021outputs/ukcensusdata.
18. Sharpe D. Your chi-square test is statistically significant: now what? Pract Assess Res Eval. 2015; 20: 1–10.
19. Von Elm E, Altman DG, Egger M, Pocock SJ, Gøtzsche PC, Vandenbroucke JP. The Strengthening the Reporting of Observational Studies in Epidemiology (STROBE) statement: guidelines for reporting observational studies. Annals of internal medicine. 2007;147(8):573-7.
20. Action on Smoking and Health (ASH) Adult Survey https://ash.org.uk/resources/view/use-of-e-cigarettes-among-adults-in-great-britain-2021.
21. Smoking Toolkit Study https://smokinginengland.info/graphs/top-line-findings.
22. Williams M, Talbot P. Design features in multiple generations of electronic cigarette atomizers. International journal of environmental research and public health. 2019;16(16):2904.
23. https://www.ons.gov.uk/peoplepopulationandcommunity/healthandsocialcare/drugusealcoholandsmoking/.
24. Lim CC, Leung JK, Connor JP, Hall WD, Gartner C, Cheng BH, et al. Availability of substances for use in personal vaporisers on three online cryptomarkets. Drug and Alcohol Dependence. 2020;217:108254.

For peer review only

Online Supplementary Material

Contents

Figures

S1: The prevalence of ever vaping and current vaping of nicotine and non-nicotine drugs

Tables

S1: All non-nicotine drug types and subtypes asked about in the online cross-sectional survey

S2: Current use (within the last 30 days) profiles for all remaining non-nicotine drugs

S3: Sociodemographic breakdown of those participants who have never vaped, ever vaped any drug including nicotine and ever vaped any drug excluding nicotine

References

For peer review only

Figure S1: The prevalence of ever vaping and current vaping of nicotine and non-nicotine drugs

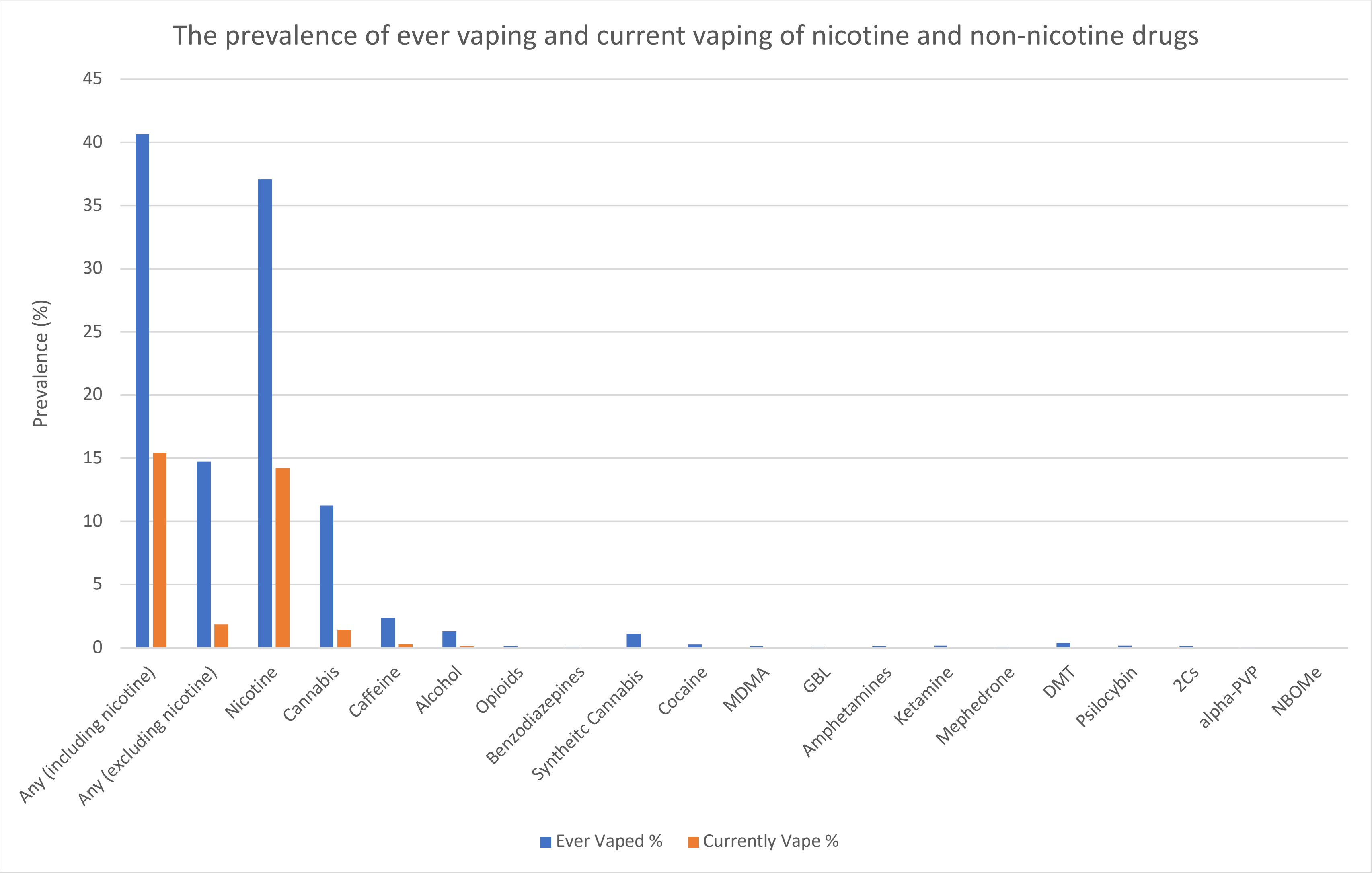

Tables

Table S1: All non-nicotine drug types and subtypes as they were detailed to participants in the online cross-sectional survey (adapted from Blundell et al. (1))

**Drug**	**Subtypes**
Nicotine	Nicotine e-liquid (freebase)<br>Nicotine salts
Caffeine	Caffeine
Alcohol	Alcohol liquid
Cannabis	Combined THC/CBD resin<br>THC resin<br>CBD resin<br>Combined THC/CBD concentrate<br>THC concentrate<br>CBD concentrate<br>Combined THC/CBD oil<br>THC oil<br>CBD oil<br>Butane hash oil<br>Dry herb/bud combined THC/CBD<br>Dry herb/bud THC<br>Dry herb/bud CBD
Synthetic Cannabis	Spice<br>Black mamba<br>Spice gold<br>Magic gold<br>Exodus<br>K2<br>Herbal incense<br>Spice arctic<br>Bonzai

For peer review only

Drug	Subtypes
	Annihilation<br>Thai high<br>AB-FUMINACA<br>ADB-PINACA<br>AM-2201<br>ADB-CHMICA<br>JWH-015<br>JWH-018<br>CP-47-497<br>5F-ADB<br>5F-AKB48<br>XLR-11<br>UR-144<br>HU-210<br>MDMB-CHMICA
Cocaine	Cocaine powder<br>Crack cocaine
MDMA	MDMA (aka Ecstasy Molly, Mandy)
GBL	GBL<br>GHB
Amphetamine	Ritalin (Methylphenidate)<br>Crystal methamphetamine (aka Ice, Tina, Crystal)
Opioids	Heroin<br>Morphine<br>Fentanyl
Ketamine	Ketamine (aka Special-K, Ket)
Mephedrone	Mephedrone (aka meow-meow, M-CAT)
DMT	DMT
Psilocybin	Psilocybin
Benzos	Diazepam (aka blues, Valium)<br>Clonazepam (aka Rivotril)<br>Alprazolam (aka Xanax)

For peer review only

Drug	Subtypes
2Cs	2CB<br>2CE
alpha-PVP	alpha-PVP aka gravelflakka
NBOMe	NBOMe (aka N-Bomb)
Other	Other, please describe

For peer review only

Table S2: Current use (within the last 30 days) profiles for all remaining non-nicotine drugs

	Ketamine	Synthetic Cannabis	Cocaine	Amphetamines	MDMA	GBL	Mephedrone	DMT	Psilocybin	2Cs	alpha-PVP	NBOMe
Currently use n (%)	1 (100%)	2 (100%)	2 (100%)	2 (100%)	0	0	0	0	0	0	0	0
Frequency of current use n (%)												
Daily	0 (0.0%)	0 (0.0%)	0 (0.0%)	1 (50.0%)	-	-	-	-	-	-	-	-
Weekly	0 (0.0%)	1 (50.0%)	1 (50.0%)	1 (50.0%)	-	-	-	-	-	-	-	-
Monthly	1 (100.0%)	1 (50.0%)	1 (50.0%)	0 (0.0%)	-	-	-	-	-	-	-	-
Currently use harmfully n (%)	0 (0.0%)	0 (0.0%)	1 (50.0%)	2 (100.0%)	-	-	-	-	-	-	-	-
Currently vape n (%)	0 (0.05)	0 (0.0%)	0 (0.0%)	0 (0.0%)	-	-	-	-	-	-	-	-
Frequency of current vaping n (%)												
Daily	-	-	-	-	-	-	-	-	-	-	-	-
Weekly	-	-	-	-	-	-	-	-	-	-	-	-
Monthly	-	-	-	-	-	-	-	-	-	-	-	-
Age first used mean (SE)	21.3 (1.38)	26.6 (1.37)	23.4 (2.14)	22.8 (3.12)	25.5 (5.17)	28 (2.74)	31.3 (4.81)	27.0 (2.51)	18.6 (1.02)	28.5 (4.14)	18.5 (3.40)	30 (N/A)
Age first vaped mean (SE)	24.4 (2.22)	27.0 (1.41)	31.3 (2.94)	25.8 (3.43)	26.5 (4.86)	30.5 (5.04)	31.7 (4.91)	27.2 (2.48)	27.4 (4.17)	35.7 (5.90)	18.5 (3.50)	30 (N/A)
t, P[1]	-1.9, 0.1	-2.3, 0.02	-2.7, 0.03	-1.4, 0.23	-2.2, 0.08	-1.0, 0.39	-1.0, 0.42	-1.0, 0.33	-1.9, 0.11	-1.1, 0.31	N/A	N/A
Modal reason for vaping initially	Because I thought I would enjoy it (42.8%)	Curiosity just wanted to try (31.1%)	Curiosity just wanted to try (30.0%)	Because I thought I would enjoy it (40.0%)	Because I thought I would enjoy it (50.0%)	Because I thought I would enjoy it (50.0%)	Curiosity just wanted to try (33.3%)	Curiosity just wanted to try (50.0%)	Curiosity just wanted to try (57.1%)	To avoid returning to other routes of administration (33.3%)	To quit other routes of administration (100.0%)	Don't know (100.0%)
Stated an intention to quit use n (%)	0 (0.0%)	0 (0.0%)	0 (0.0%)	1 (50.0%)	-	-	-	-	-	-	-	-

1 Compared using an unpaired t-test

For peer review only

Table S3: Sociodemographic breakdown of those participants who have never vaped, ever vaped any drug including nicotine and ever vaped any drug excluding nicotine

		All n (%)	Never vaped n (%)	Ever vaped any (including nicotine) n (%)	Ever vaped any (excluding nicotine) n (%)	Ever vaped any non-nicotine compared to never vapers (p)[1]
All	All	4027 (100%)	2,390 (100%)	1,637 (100%)	593 (100%)	-
Gender identity	Gender identity is the same as their sex registered at birth	3984 (98.9%)	2369 (99.1%)	1615 (98.7%)	580 (97.8%)	0.02
	Gender identity is not the same as their sex registered at birth	26 (0.7%)	12 (0.5%)	14 (0.8%)	9 (1.5%)	
	Do not wish to say	17 (0.4%)	9 (0.4%)	8 (0.5%)	4 (0.7%)	
Sexual orientation	Straight/Heterosexual	3548 (88.1%)	2176 (91.1%)	1372 (83.8%)	453 (76.4%)	<0.001
	Gay or Lesbian	153 (3.8%)	69 (2.9%)	84 (5.1%)	34 (5.7%)	
	Bisexual	237 (5.9%)	90 (3.8%)	147 (9.0%)	89 (15.0%)	
	Other sexual orientation	42 (1.0%)	26 (1.1%)	16 (1.0%)	9 (1.5%)	
	Do not wish to say	47 (1.2%)	29 (1.2%)	18 (1.1%)	8 (1.4%)	
Religion	No religion	2502 (62.1%)	1403 (58.7%)	1099 (67.1%)	424 (71.5%)	<0.001[2]
	Christian (including Church of England, Catholic, Protestant and all other Christian denominations)	1233 (30.6%)	820 (34.3%)	413 (25.2%)	112 (18.9%)	
	Buddhist	25 (0.6%)	12 (0.5%)	13 (0.8%)	6 (1.0%)	
	Hindu	34 (0.8%)	14 (0.6%)	20 (1.2%)	9 (1.5%)	
	Jewish	21 (0.5%)	13 (0.5%)	8 (0.5%)	4 (0.7%)	
	Muslim	113 (2.8%)	72 (3.0%)	41 (2.5%)	16 (2.7%)	
	Sikh	13 (0.3%)	9 (0.4%)	4 (0.2%)	1 (0.2%)	
	Any other religion	30 (0.7%)	17 (0.7%)	13 (0.8%)	10 (1.7%)	
	Do not wish to say	56 (1.4%)	30 (1.3%)	26 (1.6%)	11 (1.9%)	
Marital status	Never married and never registered in a civil partnership	1980 (49.2%)	1052 (44.0%)	928 (56.7%)	376 (63.4%)	<0.001[3]
	Married	1580 (39.2%)	1088 (45.5%)	492 (30.1%)	152 (25.6%)	
	In a registered civil partnership	56 (1.4%)	19 (0.8%)	37 (2.2%)	12 (2.0%)	
	Separated, but still legally married	51 (1.3%)	22 (0.9%)	29 (1.8%)	9 (1.5%)	
	Separated, but still legally in a civil partnership	3 (0.1%)	2 (0.1%)	1 (0.1%)	1 (0.2%)	
	Divorced	247 (6.1%)	147 (6.2%)	100 (6.1%)	30 (5.0%)	
	Formerly in a civil partnership which is now legally dissolved	4 (0.1%)	2 (0.1%)	2 (0.1%)	0 (0.0%)	
	Widowed	46 (1.1%)	30 (1.3%)	16 (1.0%)	2 (0.3%)	
	Surviving partner from a registered civil partnership	2 (0.1%)	1 (0.1%)	1 (0.1%)	0 (0.0%)	
	Do not wish to say	58 (1.4%)	27 (1.1%)	31 (1.9%)	11 (1.9%)	
Disability	Has a condition or illnesses which reduces their ability to carry out day-to-day activities	721 (17.9%)	358 (15.0%)	363 (22.2%)	163 (27.5%)	<0.001
	Does not have a condition or illnesses which reduces their ability to carry out day-to-day activities	3214 (79.8%)	1983 (83.0%)	1231 (75.2%)	413 (69.6%)	
	Do not wish to say	92 (2.3%)	49 (2.1%)	43 (2.6%)	17 (2.9%)	
Level of education	Has achieved a qualification at degree level or above	2506 (62.2%)	1526 (63.9%)	980 (59.9%)	393 (66.3%)	0.51
	Has not achieved a qualification at degree level or above	1489 (37.0%)	846 (35.4%)	643 (39.3%)	195 (32.9%)	
	Do not wish to say	32 (0.8%)	18 (0.8%)	14 (0.9%)	5 (0.8%)	

For peer review only

1 Compared using Fishers exact test; 2 For the purpose of statistical testing this category was collapsed into three levels: No religion, Christian (including Church of England, Catholic, Protestant and all other Christian denominations) and any other religion (Buddhist, Hindu, Jewish, Muslim, Sikh, Any other religion) 3 For the purpose of statistical testing this category was collapsed into three levels; Never married and never registered in a civil partnership, Married or in a registered civil partnership and any form of dissolution (Separated, but still legally married, Separated, but still legally in a civil partnership Divorced, Formerly in a civil partnership which is now legally dissolved, Widowed or Surviving partner from a registered civil partnership)

References

1. Blundell M, Dargan P, Wood D. A cloud on the horizon–a survey into the use of electronic vaping devices for recreational drug and new psychoactive substance (NPS) administration. QJM: An International Journal of Medicine. 2018;111(1):9-14.

For peer review only

Background Info

INFORMATION SHEET FOR PARTICIPANTS
Ethical Clearance Reference Number: HR-21/22-26249

Title of study : A survey about vaping drugs in the UK

Invitation Paragraph

I would like to invite you to participate in this research project. Before you decide whether you want to take part, it is important for you to understand why the research is being done and what your participation will involve. Please take time to read the following information carefully and discuss it with others if you wish. My contact details are at the end of this information sheet if you need to ask any questions before you decide to take part.

What is the purpose of the study?

The purpose of the study is to find out how common vaping is. In recent years more and more people have begun to vape nicotine and drugs that do not contain nicotine such as cannabis. People have been switching from smoking drugs to vaping drugs for several reasons e.g. it is thought to be a safer way of taking drugs. We are interested to know how common this is and how regular people vape drugs that do not contain nicotine and their reasons for vaping drugs instead of other ways of taking drugs. This will help our team of researchers, who study people who use different types of drugs, to have a better understanding of people's vaping behaviours.

Why have I been invited to take part?

You are being invited to participate in this study because you are registered with Prolific and they have identified that you meet the inclusion criteria. Our inclusion criteria is very simple, it includes anyone over the age of 18. So you don't have to have experience of vaping either nicotine or non-nicotine drugs to take part. We are trying to understand how common vaping in is the population, so it's important we get a snap shot of people who do and do not vape drugs.

What will happen if I take part?

If you agree to take part you will complete a survey anonymously. The survey will ask you questions about whether you have smoked or vaped nicotine or non-nicotine drugs and if you currently do so. If you do vape, we'll ask you questions about what substances; for example *have you ever used any type of vaping device? If you have, which of the following substances have you ever vaped? cannabis, alcohol, cocaine etc, how old were you when you first vaped each substance?* The survey will take you approximately 5-10 mins to complete, depending on your experience of vaping. We will not be asking for your name, date of birth or any other personal data that can identify you. We will ask your age category, eg 18-24, 25-34 and your sex.

Do I have to take part?

Participation is completely voluntary. You should only take part if you want to and choosing not to take part will not disadvantage you in anyway. If you choose to take part you will be asked to provide your consent. To do this you will be asked to indicate that you have read and understand the information provided and that you consent to your anonymous data being used for the purposes explained. You are free to withdraw at any point during completion of the survey, without having to give a reason. You can delete your responses to the questions before exiting the survey. You will be asked if you are sure you want to submit your answers when you reach the end of the questionnaire. Incomplete questionnaires will be unable to be submitted. Withdrawing from the study will not affect you in any way. Once you submit the survey, it will no longer be possible to withdraw from the study because the data will be fully anonymous. Please do not include any personal identifiable information in your responses.

Incentives

The usual Prolific rewards applies to this survey. Participants will be paid £7.56 per hour and and we expect the survey to take 5-10 mins.

For peer review only - http://bmjopen.bmj.com/site/about/guidelines.xhtml

What are the possible risks of taking part?

We do not forsee any possible risks of taking part as this survey is completely anonymous. If however you feel uncomfortable or would like to speak with a trained person about any drug use, we have provided contact details of national helplines at the end of the survey. Data handling and confidentiality This research is anonymous. Prolific do not hold any data related to the survey, and the researchers will not have access to the any identifiable information that Prolific hold. This means that nobody, including the researchers, will be aware of your identity, and nobody will be able to connect you to the answers you provide, even indirectly. Your answers will nevertheless be treated confidentially and the information you provide will not allow you to be identified in any research outputs/publications. Your data will be held securely in on King's College London password protected and encrypted computer data storage drive. All data will only be available to the researchers and will be handled in accordance with the UK General Data Protection Regulation (UK GDPR) and the Data Protection Act 2018. Data will be kept within the research team and not shared with a third party. All data will be kept in line with the King's College London data retention schedule and securely disposed of within 4 years.

How is the project being funded?

This study is being funded by King's College London and the Maudsley Biomedical Research Centre.

What will happen to the results of the study?

The results of the study will be summarised in a report for a journal article, which we plan to publish. The anonymous data will be kept within the research team and not shared with a third party.

Who should I contact for further information?

If you have any questions or require more information about this study, please contact me using the following contact details: deborah.j.robson@kcl.ac.uk

What if I have further questions, or if something goes wrong?

If this study has harmed you in any way or if you wish to make a complaint about the conduct of the study you can contact King's College London using the details below for further advice and information: The Chair, Health Faculties Research Ethics Sub-Committee rec@kcl.ac.uk

Thank you for reading this information sheet and for considering taking part in this research.

Consent

I confirm that I have read and understood the online information sheet presented dated 20/01/22, version #3, for the above project and I agree to take part in this research project

- ◯ Yes
- ◯ No

I consent voluntarily to be a participant in this project and understand that I can refuse to take part and can withdraw from the project before I submit my answers to the survey questions, at any time, without having to give a reason

- ◯ Yes
- ◯ No

For peer review only - http://bmjopen.bmj.com/site/about/guidelines.xhtml

I consent to the processing of my personal information for the purposes explained to me in the online Information Sheet. I understand that such information will be handled under the terms of UK data protection law, including the UK General Data Protection Regulation (UK GDPR) and the Data Protection Act 2018

- ◯ Yes
- ◯ No

I understand that my information may be subject to review by responsible individuals from King's College London for monitoring and audit purposes.

- ◯ Yes
- ◯ No

I understand that confidentiality and anonymity will be maintained, and it will not be possible to identify me in any research outputs

- ◯ Yes
- ◯ No

I understand that I must not take part if I fall under the exclusion criteria as detailed in the information sheet and explained to me by the researcher in the online information sheet.

- ◯ Yes
- ◯ No

Block 62

What is your prolific ID?

[ ]

Screening

How often, if at all, do you currently smoke ordinary tobacco cigarettes (either factory-made/packet or roll-your-own)?

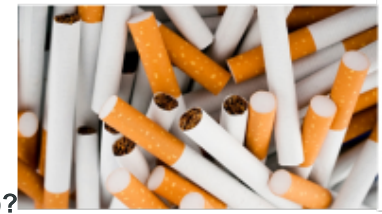

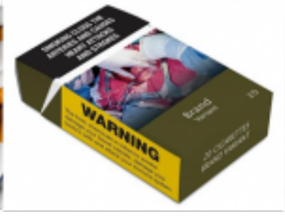

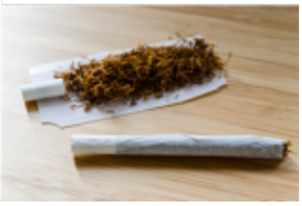

- ◯ Daily

For peer review only - http://bmjopen.bmj.com/site/about/guidelines.xhtml

- Less than daily, but at least once a week
- Less than weekly, but at least once a month
- Less than monthly, but occasionally
- I have quit smoking
- I have never been a smoker

Have you EVER used an electronic or e-cigarette?

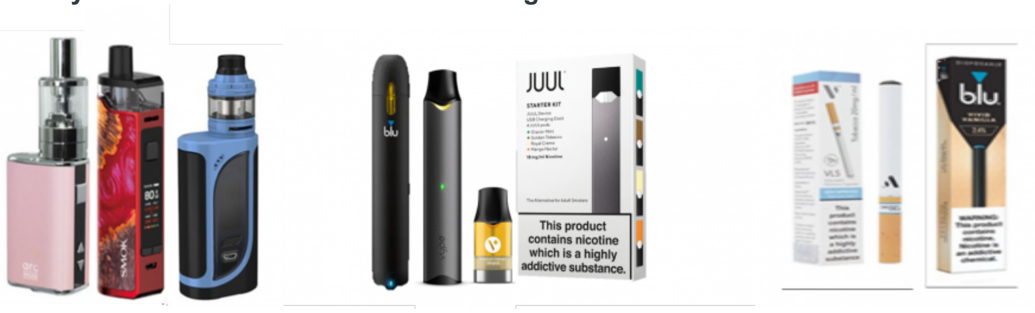

- Yes
- No

Have you EVER used electronic devices to vapourise liquids?

- Yes
- No

Have you EVER use an electronic devices to vapourise or dry herbs?

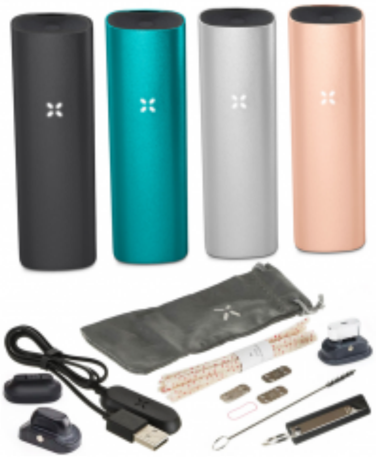

- Yes
- No

Have you EVER used an electronic table-top vaporizer?

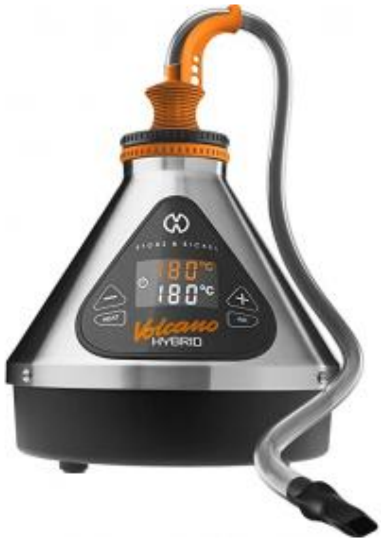

- ◯ Yes
- ◯ No

Substance use

You said that you have used an electronic vaping device. Which of the following substances have you EVER vaped using an electronic vaping device? (tick all that apply)

- ☐ Nicotine (i.e., nicotine e-liquid, salts etc.)
- ☐ Alcohol (i.e., alcohol in pure form, not as a flavouring or an additive)
- ☐ Caffeine (i.e., caffeine in pure form, not as a flavouring or an additive)
- ☐ Products containing cannabis (i.e., cannabis oils, cannabis concentrate, cannabis resin, THC, cannabidiol, CBD, butane hash oil, hash, skunk, dope, weed, pot, etc.)
- ☐ Synthetic cannabis products (i.e., spice, SCRA, black mamba etc.)
- ☐ Cocaine (i.e., powder, crack, rocks, coke, charlie etc.)
- ☐ MDMA (i.e., ecstasy, Mandy, methylenedioxymethamphetamine etc.)
- ☐ GBL (i.e., ‘G’, gamma-hydroxybutyrate, GHB, gamma-butyrolactone, liquid ecstasy etc.)
- ☐ Amphetamines (i.e., crystal methamphetamine, crystal, meth, ice, tina, speed, china white, ritalin, methylphenidate etc.)
- ☐ Opioids (i.e., heroin, fentanyl, morphine etc.)
- ☐ Ketamine (i.e., Special K, Ket etc.)
- ☐ Mephedrone (i.e., M-CAT, meow-meow etc.)
- ☐ DMT (i.e, dimethyltryptamine, ayahuasca etc.)
- ☐ Psilocybin (i.e., magic mushrooms, shrooms)
- ☐ Benzodiazepines (i.e, benzos, blues, xanax, vallium, rivitrol, diazepam, tempazepan, clonazepam etc.)
- ☐ 2C’s (i.e, 2CB, 2CE, Seventh-Heaven etc.)
- ☐ alpha-PVP (i.e., alpha-pyrrolidinopentiophenone, flakka, gravel etc.)
- ☐ NBOMe (i.e, 2, 5-dimethoxy-4-bromophenethylamine, N-Bomb etc.)
- ☐ Other- please describe

For peer review only - http://bmjopen.bmj.com/site/about/guidelines.xhtml

Vaping Nicotine

You said that you had vaped nicotine. What type of nicotine have you vaped? (tick all that apply)

- ☐ Nicotine e-liquid (freebase)
- ☐ Nicotine salts
- ☐ Other, please describe

How old were you the first time you ever used nicotine? (by ANY method not only using an electronic vaping device)

What was the first route of administration by which you ever used nicotine?

- ○ Vaping
- ○ Smoking
- ○ Oral (ingesting by mouth)
- ○ Intravenous (injecting into a vein)
- ○ Sniffing/snorting (taking by nose)
- ○ Other, please describe

How old were you when you the first time you ever vaped nicotine?

What was your main reason for vaping nicotine the first time?

- ○ To quit smoking tobacco
- ○ To cut down smoking tobacco
- ○ To use when I could not or was not allowed to smoke tobacco
- ○ To avoid returning to smoking tobacco
- ○ Because I thought I would enjoy it
- ○ Because it I thought it would give me a more pleasurable effect than other routes
- ○ Because I was attracted to the different flavours you can get in nicotine e-liquid
- ○ Because it is less harmful than smoking tobacco

For peer review only - http://bmjopen.bmj.com/site/about/guidelines.xhtml

- Because I was concerned about my health
- Curiosity just wanted to try them
- To save money
- To protect others from second-hand exposure
- For some other reason -please explain
- Don't know
- Prefer not to say

What were any other reasons for vaping nicotine the first time?

- To quit smoking tobacco
- To cut down smoking tobacco
- To use when I cannot or am not allowed to smoke tobacco
- To avoid returning to smoking tobacco
- Because I enjoy it
- Because it gives me a more pleasurable effect than other routes
- Because I like the different flavours you can get in nicotine e-liquids
- Because it is less harmful than smoking tobacco
- Because I was concerned about my health
- Curiosity just wanted to try them
- To save money
- To protect others from second-hand exposure
- For some other reason -please explain
- Don't know
- Prefer not to say

Have you used nicotine in the last 30 days?

- Yes
- No

How did you use nicotine in past 30 days?

	Yes	No
Smoked	☐	☐
Vaped	☐	☐
Nicotine replacement therapy	☐	☐
Nicotine mouth pouches	☐	☐

For peer review only - http://bmjopen.bmj.com/site/about/guidelines.xhtml

Other source of nicotine (give details)	☐	☐

Past 30 Nicotine

Do you think your use of nicotine is having a negative effect on your mental or physical health?

- Yes, on both my mental and physical health
- Yes, on my physical health only
- Yes, on my mental health only
- No

Which of the following best describes the frequency you used nicotine using any route of administration in the last 30 days?

- Daily
- Less than daily, but at least once a week
- Less than weekly, but at least once a month

Do you have any current intention to quit using nicotine?

	Yes	No	Not applicable
Quit smoking tobacco	☐	☐	☐
Quit vaping nicotine	☐	☐	☐
Quit Nicotine replacement therapy	☐	☐	☐
Quit Nicotine mouth pouches	☐	☐	☐
Quit other source of nicotine	☐	☐	☐

Have you used an electronic vaping device to use nicotine in the last 30 days?

- Yes
- No

Current Use Nicotine

What is your main reason for vaping nicotine now?

- To quit smoking tobacco
- To cut down smoking tobacco
- To use when I cannot or am not allowed to smoke tobacco

For peer review only - http://bmjopen.bmj.com/site/about/guidelines.xhtml

To avoid returning to smoking tobacco

Because I enjoy it

Because it gives me a more pleasurable effect than other routes

Because it is less harmful than smoking tobacco

Because I was concerned about my health

Curiosity just wanted to try them

To save money

To protect others from second-hand exposure

For some other reason -please explain

Don't know

Prefer not to say

What are any other reasons for vaping nicotine now?

- To quit smoking tobacco
- To cut down smoking tobacco
- To use when I cannot or am not allowed to smoke tobacco
- To avoid returning to smoking tobacco
- Because I enjoy it
- Because it gives me a more pleasurable effect than other routes
- Because it is less harmful than smoking tobacco
- Because I was concerned about my health
- Curiosity just wanted to try them
- To save money
- To protect others from second-hand exposure
- For some other reason -please explain
- Don't know
- Prefer not to say

Which of the following best describes the frequency you vaped nicotine in the last 30 days?

- Daily
- Less than daily, but at least once a week
- Less than weekly, but at least once a month

What is the main electronic vaping device by which you use nicotine?

For peer review only - http://bmjopen.bmj.com/site/about/guidelines.xhtml

- Commercially bought electronic or e-cigarette
- Commercially bought electronic device used to vaporise dry herbs
- Commercially bought tabletop vapouriser
- Commercially bought electronic device used to vape liquids (but not an e-cigarette or a tabletop vapouriser)
- Homemade electronic device
- Other, please describe

Are there any other electronic vaping devices by which you use nicotine?

- [ ] Commercially bought electronic or e-cigarette
- [ ] Commercially bought electronic device used to vaporise dry herbs
- [ ] Commercially bought tabletop vapouriser
- [ ] Commercially bought electronic device used to vape liquids (but not an e-cigarette or a tabletop vapouriser)
- [ ] Homemade electronic device
- [ ] Other, please describe

Vaping caffine

You said that you had vaped caffeine. What type of caffeine have you vaped? (tick all that apply)

- Caffeine
- Other, please describe

How old were you the first time you ever used caffeine? (by ANY method not only using an electronic vaping device)

What was the first route of administration by which you ever used caffeine?

- Vaping
- Smoking
- Oral (ingesting by mouth)
- Intravenous (injecting into a vein)
- Sniffing/snorting (taking by nose)
- Other, please describe

For peer review only - http://bmjopen.bmj.com/site/about/guidelines.xhtml

How old were you when you the first time you ever vaped caffeine?

What was your main reason for vaping caffeine the first time?

- To quit other routes of administration
- To cut down other routes of administration
- To use when I could not or was not allowed to use via other routes of administration
- To avoid returning to other routes of administration
- Because I thought I would enjoy it
- Because I though it would give me a more pleasurable effect than other routes
- Because they are less harmful than other routes of administration
- Because I was concerned about my health
- Curiosity just wanted to try them
- To save money
- To protect others from second-hand exposure
- For some other reason -please explain
- Don't know
- Prefer not to say

What were any other reasons for vaping caffeine the first time?

- To quit other routes of administration
- To cut down other routes of administration
- To use when I cannot or am not allowed to use via other routes of administration
- To avoid returning to other routes of administration
- Because I enjoy it
- Because it gives me a more pleasurable effect than other routes
- Because they are less harmful than other routes of administration
- Because I was concerned about my health
- Curiosity just wanted to try them
- To save money
- To protect others from second-hand exposure
- For some other reason -please explain
- Don't know
- Prefer not to say

For peer review only - http://bmjopen.bmj.com/site/about/guidelines.xhtml

Have you used caffeine in the last 30 days?

- Yes
- No

Past 30 Caffeine

Do you think your use of caffeine is having a negative effect on your mental or physical health?

- Yes, on both my mental and physical health
- Yes, on my physical health only
- Yes, on my mental health only
- No

Which of the following best describes the frequency you used caffeine using any route of administration in the last 30 days?

- Daily
- Less than daily, but at least once a week
- Less than weekly, but at least once a month

Do you have any current intention to quit using caffeine?

- Yes
- No

Have you used an electronic vaping device to use caffeine in the last 30 days?

- Yes
- No

Current Use Caffeine

What is your main reason for vaping caffeine now?

- To quit other routes of administration
- To cut down other routes of administration

For peer review only - http://bmjopen.bmj.com/site/about/guidelines.xhtml

- To use when I cannot or am not allowed to use via other routes of administration
- To avoid returning to other routes of administration
- Because I enjoy it
- Because it gives me a more pleasurable effect than other routes
- Because they are less harmful than other routes of administration
- Because I was concerned about my health
- Curiosity just wanted to try them
- To save money
- To protect others from second-hand exposure
- For some other reason -please explain
- Don't know
- Prefer not to say

What are any other reasons for vaping caffeine now?

- To quit other routes of administration
- To cut down other routes of administration
- To use when I cannot or am not allowed to use via other routes of administration
- To avoid returning to other routes of administration
- Because I enjoy it
- Because it gives me a more pleasurable effect than other routes
- Because they are less harmful than other routes of administration
- Because I was concerned about my health
- Curiosity just wanted to try them
- To save money
- To protect others from second-hand exposure
- For some other reason -please explain
- Don't know
- Prefer not to say

Which of the following best describes the frequency you vaped caffeine in the last 30 days?

- Daily
- Less than daily, but at least once a week
- Less than weekly, but at least once a month

What is the main electronic vaping device by which you use caffeine?

For peer review only - http://bmjopen.bmj.com/site/about/guidelines.xhtml

- Commercially bought electronic or e-cigarette
- Commercially bought electronic device used to vaporise dry herbs
- Commercially bought tabletop vapouriser
- Commercially bought electronic device used to vape liquids (but not an e-cigarette or a tabletop vapouriser)
- Homemade electronic device
- Other, please describe

What is the main electronic vaping device by which you use caffeine?

- Commercially bought electronic or e-cigarette
- Commercially bought electronic device used to vaporise dry herbs
- Commercially bought tabletop vapouriser
- Commercially bought electronic device used to vape liquids (but not an e-cigarette or a tabletop vapouriser)
- Homemade electronic device
- Other, please describe

What is the main electronic vaping device by which you use caffeine?

- Commercially bought electronic or e-cigarette
- Commercially bought electronic device used to vaporise dry herbs
- Commercially bought tabletop vapouriser
- Commercially bought electronic device used to vape liquids (but not an e-cigarette or a tabletop vapouriser)
- Homemade electronic device
- Other, please describe

Are there any other electronic vaping devices by which you use caffeine?

- Commercially bought electronic or e-cigarette
- Commercially bought electronic device used to vaporise dry herbs
- Commercially bought tabletop vapouriser
- Commercially bought electronic device used to vape liquids (but not an e-cigarette or a tabletop vapouriser)
- Homemade electronic device
- Other, please describe

Vaping Alcohol

For peer review only - http://bmjopen.bmj.com/site/about/guidelines.xhtml

You said that you had vaped alcohol. What type of alcohol have you vaped? (tick all that apply)

- Alcohol liquid
- Other, please describe

How old were you the first time you ever used alcohol? (by ANY method not only using an electronic vaping device)

What was the first route of administration by which you ever used alcohol?

- Vaping
- Oral (ingesting by mouth)
- Intravenous (injecting into a vein)
- Sniffing/snorting (taking by nose)
- Other, please describe

How old were you when you the first time you ever vaped alcohol?

What was your main reason for vaping alcohol the first time?

- To quit other routes of administration
- To cut down other routes of administration
- To use when I could not or was not allowed to use via other routes of administration
- To avoid returning to other routes of administration
- Because I thought I would enjoy it
- Because I though it would give me a more pleasurable effect than other routes
- Because they are less harmful than other routes of administration
- Because I was concerned about my health
- Curiosity just wanted to try them
- To save money
- To protect others from second-hand exposure
- For some other reason -please explain

For peer review only - http://bmjopen.bmj.com/site/about/guidelines.xhtml

- Don't know
- Prefer not to say

What were any other reasons for vaping alcohol the first time?

- To quit other routes of administration
- To cut down other routes of administration
- To use when I cannot or am not allowed to use via other routes of administration
- To avoid returning to other routes of administration
- Because I enjoy it
- Because it gives me a more pleasurable effect than other routes
- Because it is less harmful than other routes of administration
- Because I was concerned about my health
- Curiosity just wanted to try them
- To save money
- For some other reason -please explain
- Don't know
- Prefer not to say

Have you used alcohol in the last 30 days?

- Yes
- No

Past 30 Alcohol

Do you think your use of alcohol is having a negative effect on your mental or physical health?

- Yes, on both my mental and physical health
- Yes, on my physical health only
- Yes, on my mental health only
- No

Which of the following best describes the frequency you used alcohol using any route of administration in the last 30 days?

- Daily
- Less than daily, but at least once a week

For peer review only - http://bmjopen.bmj.com/site/about/guidelines.xhtml

- Less than weekly, but at least once a month

Do you have any current intention to quit using alcohol?

- Yes
- No

Have you used an electronic vaping device to use alcohol in the last 30 days?

- Yes
- No

Current alcohol use

What is your main reason for vaping alcohol now?

- To quit other routes of administration
- To cut down other routes of administration
- To use when I cannot or am not allowed to use via other routes of administration
- To avoid returning to other routes of administration
- Because I enjoy it
- Because it gives me a more pleasurable effect than other routes
- Because it is less harmful than other routes of administration
- Because I was concerned about my health
- Curiosity just wanted to try them
- To save money
- For some other reason -please explain
- Don't know
- Prefer not to say

What are any other reasons for vaping alcohol now?

- To quit other routes of administration
- To cut down other routes of administration
- To use when I cannot or am not allowed to use via other routes of administration
- To avoid returning to other routes of administration
- Because I enjoy it
- Because it gives me a more pleasurable effect than other routes

For peer review only - http://bmjopen.bmj.com/site/about/guidelines.xhtml

- [ ] Because it is less harmful than other routes of administration
- [ ] Because I was concerned about my health
- [ ] Curiosity just wanted to try them
- [ ] To save money
- [ ] For some other reason -please explain
- [ ] Don't know
- [ ] Prefer not to say

Which of the following best describes the frequency you vaped alcohol in the last 30 days?

- ○ Daily
- ○ Less than daily, but at least once a week
- ○ Less than weekly, but at least once a month

What is the main electronic vaping device by which you use alcohol?

- ○ Commercially bought electronic or e-cigarette
- ○ Commercially bought electronic device used to vaporise dry herbs
- ○ Commercially bought tabletop vapouriser
- ○ Commercially bought electronic device used to vape liquids (but not an e-cigarette or a tabletop vapouriser)
- ○ Homemade electronic device
- ○ Other, please describe

Are there any other electronic vaping devices by which you use alcohol?

- [ ] Commercially bought electronic or e-cigarette
- [ ] Commercially bought electronic device used to vaporise dry herbs
- [ ] Commercially bought tabletop vapouriser
- [ ] Commercially bought electronic device used to vape liquids (but not an e-cigarette or a tabletop vapouriser)
- [ ] Homemade electronic device
- [ ] Other, please describe

Vaping Cannabis

You said that you had vaped cannabis. What type of cannabis have you vaped? (tick all that apply)

- [ ] Combined THC/CBD resin

For peer review only - http://bmjopen.bmj.com/site/about/guidelines.xhtml

- [ ] THC resin
- [ ] CBD resin
- [ ] Combined THC/CBD concentrate
- [ ] THC concentrate
- [ ] CBD concentrate
- [ ] Combined THC/CBD oil
- [ ] THC oil
- [ ] CBD oil
- [ ] Butane hash oil
- [ ] Dry herb/bud combined THC/CBD
- [ ] Dry herb/bud THC
- [ ] Dry herb/bud CBD
- [ ] Other, please describe

How old were you the first time you ever used cannabis? (by ANY method not only using an electronic vaping device)

What was the first route of administration by which you ever used cannabis?

- Vaping
- Smoking
- Oral (ingesting by mouth)
- Intravenous (injecting into a vein)
- Sniffing/snorting (taking by nose)
- Other, please describe

How old were you when you the first time you ever vaped cannabis?

What was your main reason for vaping cannabis the first time?

- To quit other routes of administration
- To cut down other routes of administration
- To use when I could not or was not allowed to use via other routes of administration

For peer review only - http://bmjopen.bmj.com/site/about/guidelines.xhtml

- To avoid returning to other routes of administration
- Because I thought I would enjoy it
- Because I though it would give me a more pleasurable effect than other routes
- Because they are less harmful than other routes of administration
- Because I was concerned about my health
- Curiosity just wanted to try them
- To save money
- To protect others from second-hand exposure
- For some other reason -please explain
- Don't know
- Prefer not to say

What were any other reasons for vaping cannabis the first time?

- To quit other routes of administration
- To cut down other routes of administration
- To use when I cannot or am not allowed to use via other routes of administration
- To avoid returning to other routes of administration
- Because I enjoy it
- Because it gives me a more pleasurable effect than other routes
- Because they are less harmful than other routes of administration
- Because I was concerned about my health
- Curiosity just wanted to try them
- To save money
- To protect others from second-hand exposure
- For some other reason -please explain
- Don't know
- Prefer not to say

Have you used cannabis in the last 30 days?

- Yes
- No

Past 30 cannabis

Do you think your use of cannabis is having a negative effect on your mental or physical health?

For peer review only - http://bmjopen.bmj.com/site/about/guidelines.xhtml

- Yes, on both my mental and physical health
- Yes, on my physical health only
- Yes, on my mental health only
- No

Which of the following best describes the frequency you used cannabis using any route of administration in the last 30 days?

- Daily
- Less than daily, but at least once a week
- Less than weekly, but at least once a month

Do you have any current intention to quit using cannabis?

- Yes
- No

Have you used an electronic vaping device to use cannabis in the last 30 days?

- Yes
- No

Current use cannabis

What is your main reason for vaping cannabis now?

- To quit other routes of administration
- To cut down other routes of administration
- To use when I cannot or am not allowed to use via other routes of administration
- To avoid returning to other routes of administration
- Because I enjoy it
- Because it gives me a more pleasurable effect than other routes
- Because they are less harmful than other routes of administration
- Because I was concerned about my health
- Curiosity just wanted to try them
- To save money
- To protect others from second-hand exposure
- For some other reason -please explain

For peer review only - http://bmjopen.bmj.com/site/about/guidelines.xhtml

- Don't know
- Prefer not to say

What are any other reasons for vaping cannabis now?

- To quit other routes of administration
- To cut down other routes of administration
- To use when I cannot or am not allowed to use via other routes of administration
- To avoid returning to other routes of administration
- Because I enjoy it
- Because it gives me a more pleasurable effect than other routes
- Because they are less harmful than other routes of administration
- Because I was concerned about my health
- Curiosity just wanted to try them
- To save money
- To protect others from second-hand exposure
- For some other reason -please explain
- Don't know
- Prefer not to say

Which of the following best describes the frequency you vaped cannabis in the last 30 days?

- Daily
- Less than daily, but at least once a week
- Less than weekly, but at least once a month

What is the main electronic vaping device by which you use cannabis?

- Commercially bought electronic or e-cigarette
- Commercially bought electronic device used to vaporise dry herbs
- Commercially bought tabletop vapouriser
- Commercially bought electronic device used to vape liquids (but not an e-cigarette or a tabletop vapouriser)
- Homemade electronic device
- Other, please describe

Are there any other electronic vaping devices by which you use cannabis?

- Commercially bought electronic or e-cigarette
- Commercially bought electronic device used to vaporise dry herbs
- Commercially bought tabletop vapouriser
- Commercially bought electronic device used to vape liquids (but not an e-cigarette or a tabletop vapouriser)
- Homemade electronic device
- Other, please describe

Vaping Synthetic cannabis

You said that you had vaped synthetic cannabis. What type of synthetic cannabis have you vaped?

- Spice
- Black mamba
- Spice gold
- Magic gold
- Exodus
- K2
- Herbal incense
- Spice arctic
- Bonzai
- Annihilation
- Thai high
- AB-FUMINACA
- ADB-PINACA
- AM-2201
- ADB-CHMICA
- JWH-015
- JWH-018
- CP-47-497
- 5F-ADB
- 5F-AKB48
- XLR-11
- UR-144
- HU-210
- MDMB-CHMICA
- Other, please describe

How old were you the first time you ever used synthetic cannabis? (by ANY method not only using an

For peer review only - http://bmjopen.bmj.com/site/about/guidelines.xhtml

electronic vaping device)

What was the first route of administration by which you ever used synthetic cannabis?

- Vaping
- Smoking
- Oral (ingesting by mouth)
- Intravenous (injecting into a vein)
- Sniffing/snorting (taking by nose)
- Other, please describe

How old were you when you the first time you ever vaped synthetic cannabis?

What was your main reason for vaping synthetic cannabis the first time?

- To quit other routes of administration
- To cut down other routes of administration
- To use when I could not or was not allowed to use via other routes of administration
- To avoid returning to other routes of administration
- Because I thought I would enjoy it
- Because I though it would give me a more pleasurable effect than other routes
- Because they are less harmful than other routes of administration
- Because I was concerned about my health
- Curiosity just wanted to try them
- To save money
- To protect others from second-hand exposure
- For some other reason -please explain
- Don't know
- Prefer not to say

What were any other reasons for vaping synthetic cannabis the first time?

- To quit other routes of administration

For peer review only - http://bmjopen.bmj.com/site/about/guidelines.xhtml

- [ ] To cut down other routes of administration
- [ ] To use when I cannot or am not allowed to use via other routes of administration
- [ ] To avoid returning to other routes of administration
- [ ] Because I enjoy it
- [ ] Because it gives me a more pleasurable effect than other routes
- [ ] Because they are less harmful than other routes of administration
- [ ] Because I was concerned about my health
- [ ] Curiosity just wanted to try them
- [ ] To save money
- [ ] To protect others from second-hand exposure
- [ ] For some other reason -please explain
- [ ] Don't know
- [ ] Prefer not to say

Have you used synthetic cannabis in the last 30 days?

- ○ Yes
- ○ No

Past 30 synthetic cannabis

Do you think your use of synthetic cannabis is having a negative effect on your mental or physical health?

- ○ Yes, on both my mental and physical health
- ○ Yes, on my physical health only
- ○ Yes, on my mental health only
- ○ No

Which of the following best describes the frequency you used synthetic cannabis using any route of administration in the last 30 days?

- ○ Daily
- ○ Less than daily, but at least once a week
- ○ Less than weekly, but at least once a month

Do you have any current intention to quit using synthetic cannabis?

- ○ Yes
- ○

For peer review only - http://bmjopen.bmj.com/site/about/guidelines.xhtml

No

Have you used an electronic vaping device to use synthetic cannabis in the last 30 days?

- Yes
- No

Current synthetic cannabis use

What is your main reason for vaping synthetic cannabis now?

- To quit other routes of administration
- To cut down other routes of administration
- To use when I cannot or am not allowed to use via other routes of administration
- To avoid returning to other routes of administration
- Because I enjoy it
- Because it gives me a more pleasurable effect than other routes
- Because they are less harmful than other routes of administration
- Because I was concerned about my health
- Curiosity just wanted to try them
- To save money
- To protect others from second-hand exposure
- For some other reason -please explain
- Don't know
- Prefer not to say

What are any other reasons for vaping synthetic cannabis now?

- To quit other routes of administration
- To cut down other routes of administration
- To use when I cannot or am not allowed to use via other routes of administration
- To avoid returning to other routes of administration
- Because I enjoy it
- Because it gives me a more pleasurable effect than other routes
- Because they are less harmful than other routes of administration
- Because I was concerned about my health
- Curiosity just wanted to try them
- To save money

For peer review only - http://bmjopen.bmj.com/site/about/guidelines.xhtml

To protect others from second-hand exposure

For some other reason -please explain

Don't know

Prefer not to say

Which of the following best describes the frequency you vaped synthetic cannabis in the last 30 days?

Daily

Less than daily, but at least once a week

Less than weekly, but at least once a month

What is the main electronic vaping device by which you use synthetic cannabis?

Commercially bought electronic or e-cigarette

Commercially bought electronic device used to vaporise dry herbs

Commercially bought tabletop vapouriser

Commercially bought electronic device used to vape liquids (but not an e-cigarette or a tabletop vapouriser)

Homemade electronic device

Other, please describe

Are there any other electronic vaping devices by which you use synthetic cannabis?

Commercially bought electronic or e-cigarette

Commercially bought electronic device used to vaporise dry herbs

Commercially bought tabletop vapouriser

Commercially bought electronic device used to vape liquids (but not an e-cigarette or a tabletop vapouriser)

Homemade electronic device

Other, please describe

Cocaine vaping

You said that you had vaped cocaine. What type of cocaine have you vaped? (tick all that apply)

Cocaine powder

Crack cocaine

Other, please describe

For peer review only - http://bmjopen.bmj.com/site/about/guidelines.xhtml

How old were you the first time you ever used cocaine? (by ANY method not only using an electronic vaping device)

What was the first route of administration by which you ever used cocaine?

- Vaping
- Smoking
- Oral (ingesting by mouth)
- Intravenous (injecting into a vein)
- Sniffing/snorting (taking by nose)
- Other, please describe

How old were you when you the first time you ever vaped cocaine?

What was your main reason for vaping cocaine the first time?

- To quit other routes of administration
- To cut down other routes of administration
- To use when I could not or was not allowed to use via other routes of administration
- To avoid returning to other routes of administration
- Because I thought I would enjoy it
- Because I though it would give me a more pleasurable effect than other routes
- Because they are less harmful than other routes of administration
- Because I was concerned about my health
- Curiosity just wanted to try them
- To save money
- To protect others from second-hand exposure
- For some other reason -please explain
- Don't know
- Prefer not to say

What were any other reasons for vaping cocaine the first time?

For peer review only - http://bmjopen.bmj.com/site/about/guidelines.xhtml

- To quit other routes of administration
- To cut down other routes of administration
- To use when I cannot or am not allowed to use via other routes of administration
- To avoid returning to other routes of administration
- Because I enjoy it
- Because it gives me a more pleasurable effect than other routes
- Because they are less harmful than other routes of administration
- Because I was concerned about my health
- Curiosity just wanted to try them
- To save money
- To protect others from second-hand exposure
- For some other reason -please explain
- Don't know
- Prefer not to say

Have you used cocaine in the last 30 days?

- Yes
- No

Past 30 cocaine

Do you think your use of cocaine is having a negative effect on your mental or physical health?

- Yes, on both my mental and physical health
- Yes, on my physical health only
- Yes, on my mental health only
- No

Which of the following best describes the frequency you used cocaine using any route of administration in the last 30 days?

- Daily
- Less than daily, but at least once a week
- Less than weekly, but at least once a month

Do you have any current intention to quit using cocaine?

For peer review only - http://bmjopen.bmj.com/site/about/guidelines.xhtml

- Yes
- No

Have you used an electronic vaping device to use cocaine in the last 30 days?

- Yes
- No

Current use cocaine

What is your main reason for vaping cocaine now?

- To quit other routes of administration
- To cut down other routes of administration
- To use when I cannot or am not allowed to use via other routes of administration
- To avoid returning to other routes of administration
- Because I enjoy it
- Because it gives me a more pleasurable effect than other routes
- Because they are less harmful than other routes of administration
- Because I was concerned about my health
- Curiosity just wanted to try them
- To save money
- To protect others from second-hand exposure
- For some other reason -please explain
- Don't know
- Prefer not to say

What are any other reasons for vaping cocaine now?

- To quit other routes of administration
- To cut down other routes of administration
- To use when I cannot or am not allowed to use via other routes of administration
- To avoid returning to other routes of administration
- Because I enjoy it
- Because it gives me a more pleasurable effect than other routes
- Because they are less harmful than other routes of administration
- Because I was concerned about my health
- Curiosity just wanted to try them

For peer review only - http://bmjopen.bmj.com/site/about/guidelines.xhtml

- [ ] To save money
- [ ] To protect others from second-hand exposure
- [ ] For some other reason -please explain
- [ ] Don't know
- [ ] Prefer not to say

Which of the following best describes the frequency you vaped cocaine in the last 30 days?

- Daily
- Less than daily, but at least once a week
- Less than weekly, but at least once a month

What is the main electronic vaping device by which you use cocaine?

- Commercially bought electronic or e-cigarette
- Commercially bought electronic device used to vaporise dry herbs
- Commercially bought tabletop vapouriser
- Commercially bought electronic device used to vape liquids (but not an e-cigarette or a tabletop vapouriser)
- Homemade electronic device
- Other, please describe

Are there any other electronic vaping devices by which you use cocaine?

- [ ] Commercially bought electronic or e-cigarette
- [ ] Commercially bought electronic device used to vaporise dry herbs
- [ ] Commercially bought tabletop vapouriser
- [ ] Commercially bought electronic device used to vape liquids (but not an e-cigarette or a tabletop vapouriser)
- [ ] Homemade electronic device
- [ ] Other, please describe

MDMA Vaping

You said that you had vaped MDMA. What type of MDMA have you vaped? (tick all that apply)

- [ ] MDMA (aka Ecstasy Molly, Mandy)
- [ ] Other, please describe

For peer review only - http://bmjopen.bmj.com/site/about/guidelines.xhtml

How old were you the first time you ever used MDMA? (by ANY method not only using an electronic vaping device)

What was the first route of administration by which you ever used MDMA?

- Vaping
- Smoking
- Oral (ingesting by mouth)
- Intravenous (injecting into a vein)
- Sniffing/snorting (taking by nose)
- Other, please describe

How old were you when you the first time you ever vaped MDMA?

What was your main reason for vaping MDMA the first time?

- To quit other routes of administration
- To cut down other routes of administration
- To use when I could not or was not allowed to use via other routes of administration
- To avoid returning to other routes of administration
- Because I thought I would enjoy it
- Because I though it would give me a more pleasurable effect than other routes
- Because they are less harmful than other routes of administration
- Because I was concerned about my health
- Curiosity just wanted to try them
- To save money
- To protect others from second-hand exposure
- For some other reason -please explain
- Don't know
- Prefer not to say

For peer review only - http://bmjopen.bmj.com/site/about/guidelines.xhtml

What were any other reasons for vaping MDMA the first time?

- ☐ To quit other routes of administration
- ☐ To cut down other routes of administration
- ☐ To use when I cannot or am not allowed to use via other routes of administration
- ☐ To avoid returning to other routes of administration
- ☐ Because I enjoy it
- ☐ Because it gives me a more pleasurable effect than other routes
- ☐ Because they are less harmful than other routes of administration
- ☐ Because I was concerned about my health
- ☐ Curiosity just wanted to try them
- ☐ To save money
- ☐ To protect others from second-hand exposure
- ☐ For some other reason -please explain
- ☐ Don't know
- ☐ Prefer not to say

Have you used MDMA in the last 30 days?

- ◯ Yes
- ◯ No

Past 30 MDMA

Do you think your use of MDMA is having a negative effect on your mental or physical health?

- ◯ Yes, on both my mental and physical health
- ◯ Yes, on my physical health only
- ◯ Yes, on my mental health only
- ◯ No

Which of the following best describes the frequency you used MDMA using any route of administration in the last 30 days?

- ◯ Daily
- ◯ Less than daily, but at least once a week
- ◯ Less than weekly, but at least once a month

For peer review only - http://bmjopen.bmj.com/site/about/guidelines.xhtml

Do you have any current intention to quit using MDMA?

- Yes
- No

Have you used an electronic vaping device to use MDMA in the last 30 days?

- Yes
- No

Current use MDMA

What is your main reason for vaping MDMA now?

- To quit other routes of administration
- To cut down other routes of administration
- To use when I cannot or am not allowed to use via other routes of administration
- To avoid returning to other routes of administration
- Because I enjoy it
- Because it gives me a more pleasurable effect than other routes
- Because they are less harmful than other routes of administration
- Because I was concerned about my health
- Curiosity just wanted to try them
- To save money
- To protect others from second-hand exposure
- For some other reason -please explain
- Don't know
- Prefer not to say

What are any other reasons for vaping MDMA now?

- To quit other routes of administration
- To cut down other routes of administration
- To use when I cannot or am not allowed to use via other routes of administration
- To avoid returning to other routes of administration
- Because I enjoy it
- Because it gives me a more pleasurable effect than other routes
- Because they are less harmful than other routes of administration
- Because I was concerned about my health

For peer review only - http://bmjopen.bmj.com/site/about/guidelines.xhtml

- [ ] Curiosity just wanted to try them
- [ ] To save money
- [ ] To protect others from second-hand exposure
- [ ] For some other reason -please explain
- [ ] Don't know
- [ ] Prefer not to say

Which of the following best describes the frequency you vaped MDMA in the last 30 days?

- ○ Daily
- ○ Less than daily, but at least once a week
- ○ Less than weekly, but at least once a month

What is the main electronic vaping device by which you use MDMA?

- ○ Commercially bought electronic or e-cigarette
- ○ Commercially bought electronic device used to vaporise dry herbs
- ○ Commercially bought tabletop vapouriser
- ○ Commercially bought electronic device used to vape liquids (but not an e-cigarette or a tabletop vapouriser)
- ○ Homemade electronic device
- ○ Other, please describe

Are there any other electronic vaping devices by which you use MDMA?

- [ ] Commercially bought electronic or e-cigarette
- [ ] Commercially bought electronic device used to vaporise dry herbs
- [ ] Commercially bought tabletop vapouriser
- [ ] Commercially bought electronic device used to vape liquids (but not an e-cigarette or a tabletop vapouriser)
- [ ] Homemade electronic device
- [ ] Other, please describe

GBL Vaping

You said that you had vaped GBL. What type of GBL have you vaped? (tick all that apply)

- [ ] GBL (aka liquid ecstasy)
- [ ] GHB

For peer review only - http://bmjopen.bmj.com/site/about/guidelines.xhtml

☐ Other, please describe

How old were you the first time you ever used GBL? (by ANY method not only using an electronic vaping device)

What was the first route of administration by which you ever used GBL?

- Vaping
- Smoking
- Oral (ingesting by mouth)
- Intravenous (injecting into a vein)
- Sniffing/snorting (taking by nose)
- Other, please describe

How old were you when you the first time you ever vaped GBL?

What was your main reason for vaping GBL the first time?

- To quit other routes of administration
- To cut down other routes of administration
- To use when I could not or was not allowed to use via other routes of administration
- To avoid returning to other routes of administration
- Because I thought I would enjoy it
- Because I though it would give me a more pleasurable effect than other routes
- Because they are less harmful than other routes of administration
- Because I was concerned about my health
- Curiosity just wanted to try them
- To save money
- To protect others from second-hand exposure
- For some other reason -please explain
- Don't know
- Prefer not to say

For peer review only - http://bmjopen.bmj.com/site/about/guidelines.xhtml

What were any other reasons for vaping GBL the first time?

- ☐ To quit other routes of administration
- ☐ To cut down other routes of administration
- ☐ To use when I cannot or am not allowed to use via other routes of administration
- ☐ To avoid returning to other routes of administration
- ☐ Because I enjoy it
- ☐ Because it gives me a more pleasurable effect than other routes
- ☐ Because they are less harmful than other routes of administration
- ☐ Because I was concerned about my health
- ☐ Curiosity just wanted to try them
- ☐ To save money
- ☐ To protect others from second-hand exposure
- ☐ For some other reason -please explain
- ☐ Don't know
- ☐ Prefer not to say

Have you used GBL in the last 30 days?

- ○ Yes
- ○ No

Past 30 day GLB

Do you think your use of GLB is having a negative effect on your mental or physical health?

- ○ Yes, on both my mental and physical health
- ○ Yes, on my physical health only
- ○ Yes, on my mental health only
- ○ No

Which of the following best describes the frequency you used GLB using any route of administration in the last 30 days?

- ○ Daily
- ○ Less than daily, but at least once a week
- ○ Less than weekly, but at least once a month

For peer review only - http://bmjopen.bmj.com/site/about/guidelines.xhtml

Do you have any current intention to quit using GLB?

- ◯ Yes
- ◯ No

Have you used an electronic vaping device to use GLB in the last 30 days?

- ◯ Yes
- ◯ No

Current use GBL

What is your main reason for vaping GBL now?

- ◯ To quit other routes of administration
- ◯ To cut down other routes of administration
- ◯ To use when I cannot or am not allowed to use via other routes of administration
- ◯ To avoid returning to other routes of administration
- ◯ Because I enjoy it
- ◯ Because it gives me a more pleasurable effect than other routes
- ◯ Because they are less harmful than other routes of administration
- ◯ Because I was concerned about my health
- ◯ Curiosity just wanted to try them
- ◯ To save money
- ◯ To protect others from second-hand exposure
- ◯ For some other reason -please explain ______
- ◯ Don't know
- ◯ Prefer not to say

What are any other reasons for vaping GBL now?

- ☐ To quit other routes of administration
- ☐ To cut down other routes of administration
- ☐ To use when I cannot or am not allowed to use via other routes of administration
- ☐ To avoid returning to other routes of administration
- ☐ Because I enjoy it
- ☐ Because it gives me a more pleasurable effect than other routes
- ☐ Because they are less harmful than other routes of administration

For peer review only - http://bmjopen.bmj.com/site/about/guidelines.xhtml

- [ ] Because I was concerned about my health
- [ ] Curiosity just wanted to try them
- [ ] To save money
- [ ] To protect others from second-hand exposure
- [ ] For some other reason -please explain
- [ ] Don't know
- [ ] Prefer not to say

Which of the following best describes the frequency you vaped GBL in the last 30 days?

- ○ Daily
- ○ Less than daily, but at least once a week
- ○ Less than weekly, but at least once a month

What is the main electronic vaping device by which you use GBL?

- ○ Commercially bought electronic or e-cigarette
- ○ Commercially bought electronic device used to vaporise dry herbs
- ○ Commercially bought tabletop vapouriser
- ○ Commercially bought electronic device used to vape liquids (but not an e-cigarette or a tabletop vapouriser)
- ○ Homemade electronic device
- ○ Other, please describe

Are there any other electronic vaping devices by which you use GBL?

- [ ] Commercially bought electronic or e-cigarette
- [ ] Commercially bought electronic device used to vaporise dry herbs
- [ ] Commercially bought tabletop vapouriser
- [ ] Commercially bought electronic device used to vape liquids (but not an e-cigarette or a tabletop vapouriser)
- [ ] Homemade electronic device
- [ ] Other, please describe

Vaping Amphetamines

You said that you had vaped amphetamines. What type of amphetamines have you vaped? (tick all that apply)

- [ ] Ritalin (Methylphenidate)

For peer review only - http://bmjopen.bmj.com/site/about/guidelines.xhtml

- Crystal methamphetamine (aka Ice, Tina, Crystal)
- Other, please describe

How old were you the first time you ever used amphetamines? (by ANY method not only using an electronic vaping device)

What was the first route of administration by which you ever used amphetamines?

- Vaping
- Smoking
- Oral (ingesting by mouth)
- Intravenous (injecting into a vein)
- Sniffing/snorting (taking by nose)
- Other, please describe

How old were you when you the first time you ever vaped amphetamines?

What was your main reason for vaping amphetamines the first time?

- To quit other routes of administration
- To cut down other routes of administration
- To use when I could not or was not allowed to use via other routes of administration
- To avoid returning to other routes of administration
- Because I thought I would enjoy it
- Because I though it would give me a more pleasurable effect than other routes
- Because they are less harmful than other routes of administration
- Because I was concerned about my health
- Curiosity just wanted to try them
- To save money
- To protect others from second-hand exposure
- For some other reason -please explain
- Don't know

For peer review only - http://bmjopen.bmj.com/site/about/guidelines.xhtml

Prefer not to say

What were any other reasons for vaping amphetamines the first time?

- To quit other routes of administration
- To cut down other routes of administration
- To use when I cannot or am not allowed to use via other routes of administration
- To avoid returning to other routes of administration
- Because I enjoy it
- Because it gives me a more pleasurable effect than other routes
- Because they are less harmful than other routes of administration
- Because I was concerned about my health
- Curiosity just wanted to try them
- To save money
- To protect others from second-hand exposure
- For some other reason -please explain
- Don't know
- Prefer not to say

Have you used amphetamines in the last 30 days?

- Yes
- No

Past 30 amphetamines

Do you think your use of amphetamines is having a negative effect on your mental or physical health?

- Yes, on both my mental and physical health
- Yes, on my physical health only
- Yes, on my mental health only
- No

Which of the following best describes the frequency you used amphetamines using any route of administration in the last 30 days?

- Daily
- Less than daily, but at least once a week

For peer review only - http://bmjopen.bmj.com/site/about/guidelines.xhtml

Less than weekly, but at least once a month

Do you have any current intention to quit using amphetamines?

- Yes
- No

Have you used an electronic vaping device to use amphetamines in the last 30 days?

- Yes
- No

Current use amphetamines

What is your main reason for vaping amphetamines now?

- To quit other routes of administration
- To cut down other routes of administration
- To use when I cannot or am not allowed to use via other routes of administration
- To avoid returning to other routes of administration
- Because I enjoy it
- Because it gives me a more pleasurable effect than other routes
- Because they are less harmful than other routes of administration
- Because I was concerned about my health
- Curiosity just wanted to try them
- To save money
- To protect others from second-hand exposure
- For some other reason -please explain
- Don't know
- Prefer not to say

What are any other reasons for vaping amphetamines now?

- To quit other routes of administration
- To cut down other routes of administration
- To use when I cannot or am not allowed to use via other routes of administration
- To avoid returning to other routes of administration
- Because I enjoy it

- [ ] Because it gives me a more pleasurable effect than other routes
- [ ] Because they are less harmful than other routes of administration
- [ ] Because I was concerned about my health
- [ ] Curiosity just wanted to try them
- [ ] To save money
- [ ] To protect others from second-hand exposure
- [ ] For some other reason -please explain
- [ ] Don't know
- [ ] Prefer not to say

Which of the following best describes the frequency you vaped amphetamines in the last 30 days?

- ( ) Daily
- ( ) Less than daily, but at least once a week
- ( ) Less than weekly, but at least once a month

What is the main electronic vaping device by which you use amphetamines?

- ( ) Commercially bought electronic or e-cigarette
- ( ) Commercially bought electronic device used to vaporise dry herbs
- ( ) Commercially bought tabletop vapouriser
- ( ) Commercially bought electronic device used to vape liquids (but not an e-cigarette or a tabletop vapouriser)
- ( ) Homemade electronic device
- ( ) Other, please describe

Are there any other electronic vaping devices by which you use amphetamines?

- [ ] Commercially bought electronic or e-cigarette
- [ ] Commercially bought electronic device used to vaporise dry herbs
- [ ] Commercially bought tabletop vapouriser
- [ ] Commercially bought electronic device used to vape liquids (but not an e-cigarette or a tabletop vapouriser)
- [ ] Homemade electronic device
- [ ] Other, please describe

Vaping Opiods

For peer review only - http://bmjopen.bmj.com/site/about/guidelines.xhtml

You said that you had vaped opioids. What type of opioids have you vaped? (tick all that apply)

- ☐ Heroin
- ☐ Morphine
- ☐ Fentanyl
- ☐ Other, please describe

How old were you the first time you ever used opioids? (by ANY method not only using an electronic vaping device)

What was the first route of administration by which you ever used opioids?

- ◯ Vaping
- ◯ Smoking
- ◯ Oral (ingesting by mouth)
- ◯ Intravenous (injecting into a vein)
- ◯ Sniffing/snorting (taking by nose)
- ◯ Other, please describe

How old were you when you the first time you ever vaped opioids?

What was your main reason for vaping opioids the first time?

- ◯ To quit other routes of administration
- ◯ To cut down other routes of administration
- ◯ To use when I could not or was not allowed to use via other routes of administration
- ◯ To avoid returning to other routes of administration
- ◯ Because I thought I would enjoy it
- ◯ Because I though it would give me a more pleasurable effect than other routes
- ◯ Because they are less harmful than other routes of administration
- ◯ Because I was concerned about my health
- ◯ Curiosity just wanted to try them
- ◯ To save money

For peer review only - http://bmjopen.bmj.com/site/about/guidelines.xhtml

- To protect others from second-hand exposure
- For some other reason -please explain
- Don't know
- Prefer not to say

What were any other reasons for vaping opioids the first time?

- To quit other routes of administration
- To cut down other routes of administration
- To use when I cannot or am not allowed to use via other routes of administration
- To avoid returning to other routes of administration
- Because I enjoy it
- Because it gives me a more pleasurable effect than other routes
- Because they are less harmful than other routes of administration
- Because I was concerned about my health
- Curiosity just wanted to try them
- To save money
- To protect others from second-hand exposure
- For some other reason -please explain
- Don't know
- Prefer not to say

Have you used opioids in the last 30 days?

- Yes
- No

Past 30 opioids

Do you think your use of opioids is having a negative effect on your mental or physical health?

- Yes, on both my mental and physical health
- Yes, on my physical health only
- Yes, on my mental health only
- No

Which of the following best describes the frequency you used opioids using any route of administration in

For peer review only - http://bmjopen.bmj.com/site/about/guidelines.xhtml

the last 30 days?

- Daily
- Less than daily, but at least once a week
- Less than weekly, but at least once a month

Do you have any current intention to quit using opioids?

- Yes
- No

Have you used an electronic vaping device to use opioids in the last 30 days?

- Yes
- No

Current opioids use

What is your main reason for vaping opioids now?

- To quit other routes of administration
- To cut down other routes of administration
- To use when I cannot or am not allowed to use via other routes of administration
- To avoid returning to other routes of administration
- Because I enjoy it
- Because it gives me a more pleasurable effect than other routes
- Because they are less harmful than other routes of administration
- Because I was concerned about my health
- Curiosity just wanted to try them
- To save money
- To protect others from second-hand exposure
- For some other reason -please explain
- Don't know
- Prefer not to say

What are any other reasons for vaping opioids now?

- To quit other routes of administration
- To cut down other routes of administration

For peer review only - http://bmjopen.bmj.com/site/about/guidelines.xhtml

- To use when I cannot or am not allowed to use via other routes of administration
- To avoid returning to other routes of administration
- Because I enjoy it
- Because it gives me a more pleasurable effect than other routes
- Because they are less harmful than other routes of administration
- Because I was concerned about my health
- Curiosity just wanted to try them
- To save money
- To protect others from second-hand exposure
- For some other reason -please explain
- Don't know
- Prefer not to say

Which of the following best describes the frequency you vaped opioids in the last 30 days?

- Daily
- Less than daily, but at least once a week
- Less than weekly, but at least once a month

What is the main electronic vaping device by which you use opioids?

- Commercially bought electronic or e-cigarette
- Commercially bought electronic device used to vaporise dry herbs
- Commercially bought tabletop vapouriser
- Commercially bought electronic device used to vape liquids (but not an e-cigarette or a tabletop vapouriser)
- Homemade electronic device
- Other, please describe

Are there any other electronic vaping devices by which you use opioids?

- Commercially bought electronic or e-cigarette
- Commercially bought electronic device used to vaporise dry herbs
- Commercially bought tabletop vapouriser
- Commercially bought electronic device used to vape liquids (but not an e-cigarette or a tabletop vapouriser)
- Homemade electronic device
- Other, please describe

Vaping ketamine

You said that you had vaped ketamine. What type of ketamine have you vaped? (tick all that apply)

- ☐ Ketamine (aka Special-K, Ket)
- ☐ Other, please describe

How old were you the first time you ever used ketamine? (by ANY method not only using an electronic vaping device)

What was the first route of administration by which you ever used ketamine?

- ○ Vaping
- ○ Smoking
- ○ Oral (ingesting by mouth)
- ○ Intravenous (injecting into a vein)
- ○ Sniffing/snorting (taking by nose)
- ○ Other, please describe

How old were you when you the first time you ever vaped ketamine?

What was your main reason for vaping ketamine the first time?

- ○ To quit other routes of administration
- ○ To cut down other routes of administration
- ○ To use when I could not or was not allowed to use via other routes of administration
- ○ To avoid returning to other routes of administration
- ○ Because I thought I would enjoy it
- ○ Because I though it would give me a more pleasurable effect than other routes
- ○ Because they are less harmful than other routes of administration
- ○ Because I was concerned about my health
- ○ Curiosity just wanted to try them
- ○ To save money

For peer review only - http://bmjopen.bmj.com/site/about/guidelines.xhtml

- To protect others from second-hand exposure
- For some other reason -please explain
- Don't know
- Prefer not to say

What were any other reasons for vaping ketamine the first time?

- To quit other routes of administration
- To cut down other routes of administration
- To use when I cannot or am not allowed to use via other routes of administration
- To avoid returning to other routes of administration
- Because I enjoy it
- Because it gives me a more pleasurable effect than other routes
- Because they are less harmful than other routes of administration
- Because I was concerned about my health
- Curiosity just wanted to try them
- To save money
- To protect others from second-hand exposure
- For some other reason -please explain
- Don't know
- Prefer not to say

Have you used ketamine in the last 30 days?

- Yes
- No

Past 30 days ketamine

Do you think your use of ketamine is having a negative effect on your mental or physical health?

- Yes, on both my mental and physical health
- Yes, on my physical health only
- Yes, on my mental health only
- No

For peer review only - http://bmjopen.bmj.com/site/about/guidelines.xhtml

Which of the following best describes the frequency you used ketamine using any route of administration in the last 30 days?

- Daily
- Less than daily, but at least once a week
- Less than weekly, but at least once a month

Do you have any current intention to quit using ketamine?

- Yes
- No

Have you used an electronic vaping device to use ketamine in the last 30 days?

- Yes
- No

Current use ketamine

What is your main reason for vaping ketamine now?

- To quit other routes of administration
- To cut down other routes of administration
- To use when I cannot or am not allowed to use via other routes of administration
- To avoid returning to other routes of administration
- Because I enjoy it
- Because it gives me a more pleasurable effect than other routes
- Because they are less harmful than other routes of administration
- Because I was concerned about my health
- Curiosity just wanted to try them
- To save money
- To protect others from second-hand exposure
- For some other reason -please explain
- Don't know
- Prefer not to say

What are any other reasons for vaping ketamine now?

For peer review only - http://bmjopen.bmj.com/site/about/guidelines.xhtml

- To quit other routes of administration
- To cut down other routes of administration
- To use when I cannot or am not allowed to use via other routes of administration
- To avoid returning to other routes of administration
- Because I enjoy it
- Because it gives me a more pleasurable effect than other routes
- Because they are less harmful than other routes of administration
- Because I was concerned about my health
- Curiosity just wanted to try them
- J. To save money
- To protect others from second-hand exposure
- For some other reason -please explain
- Don't know
- Prefer not to say

Which of the following best describes the frequency you vaped ketamine in the last 30 days?

- Daily
- Less than daily, but at least once a week
- Less than weekly, but at least once a month

What is the main electronic vaping device by which you use ketamine?

- Commercially bought electronic or e-cigarette
- Commercially bought electronic device used to vaporise dry herbs
- Commercially bought tabletop vapouriser
- Commercially bought electronic device used to vape liquids (but not an e-cigarette or a tabletop vapouriser)
- Homemade electronic device
- Other, please describe

Are there any other electronic vaping devices by which you use ketamine?

- Commercially bought electronic or e-cigarette
- Commercially bought electronic device used to vaporise dry herbs
- Commercially bought tabletop vapouriser
- Commercially bought electronic device used to vape liquids (but not an e-cigarette or a tabletop vapouriser)
- Homemade electronic device

For peer review only - http://bmjopen.bmj.com/site/about/guidelines.xhtml

Other, please describe

Vaping Mephedrone

You said that you had vaped mephedrone. What type of mephedrone have you vaped? (tick all that apply)

- Mephedrone (aka meow-meow, M-CAT)
- Other, please describe

How old were you the first time you ever used mephedrone? (by ANY method not only using an electronic vaping device)

What was the first route of administration by which you ever used mephedrone?

- Vaping
- Smoking
- Oral (ingesting by mouth)
- Intravenous (injecting into a vein)
- Sniffing/snorting (taking by nose)
- Other, please describe

How old were you when you the first time you ever vaped mephedrone?

What was your main reason for vaping mephedrone the first time?

- To quit other routes of administration
- To cut down other routes of administration
- To use when I could not or was not allowed to use via other routes of administration
- To avoid returning to other routes of administration
- Because I thought I would enjoy it
- Because I though it would give me a more pleasurable effect than other routes
- Because they are less harmful than other routes of administration

- Because I was concerned about my health
- Curiosity just wanted to try them
- To save money
- To protect others from second-hand exposure
- For some other reason -please explain
- Don't know
- Prefer not to say

What were any other reasons for vaping mephedrone the first time?

- To quit other routes of administration
- To cut down other routes of administration
- To use when I cannot or am not allowed to use via other routes of administration
- To avoid returning to other routes of administration
- Because I enjoy it
- Because it gives me a more pleasurable effect than other routes
- Because they are less harmful than other routes of administration
- Because I was concerned about my health
- Curiosity just wanted to try them
- To save money
- To protect others from second-hand exposure
- For some other reason -please explain
- Don't know
- Prefer not to say

Have you used mephedrone in the last 30 days?

- Yes
- No

Past 30 mephedrone

Do you think your use of mephedrone is having a negative effect on your mental or physical health?

- Yes, on both my mental and physical health
- Yes, on my physical health only
- Yes, on my mental health only
- No

Which of the following best describes the frequency you used mephedrone using any route of administration in the last 30 days?

- Daily
- Less than daily, but at least once a week
- Less than weekly, but at least once a month

Do you have any current intention to quit using mephedrone?

- Yes
- No

Have you used an electronic vaping device to use mephedrone in the last 30 days?

- Yes
- No

Current use mephedrone

What is your main reason for vaping mephedrone now?

- To quit other routes of administration
- To cut down other routes of administration
- To use when I cannot or am not allowed to use via other routes of administration
- To avoid returning to other routes of administration
- Because I enjoy it
- Because it gives me a more pleasurable effect than other routes
- Because they are less harmful than other routes of administration
- Because I was concerned about my health
- Curiosity just wanted to try them
- To save money
- To protect others from second-hand exposure
- For some other reason -please explain
- Don't know
- Prefer not to say

For peer review only - http://bmjopen.bmj.com/site/about/guidelines.xhtml

What are any other reasons for vaping mephedrone now?

- ☐ To quit other routes of administration
- ☐ To cut down other routes of administration
- ☐ To use when I cannot or am not allowed to use via other routes of administration
- ☐ To avoid returning to other routes of administration
- ☐ Because I enjoy it
- ☐ Because it gives me a more pleasurable effect than other routes
- ☐ Because they are less harmful than other routes of administration
- ☐ Because I was concerned about my health
- ☐ Curiosity just wanted to try them
- ☐ To save money
- ☐ To protect others from second-hand exposure
- ☐ For some other reason -please explain
- ☐ Don't know
- ☐ Prefer not to say

Which of the following best describes the frequency you vaped mephedrone in the last 30 days?

- ○ Daily
- ○ Less than daily, but at least once a week
- ○ Less than weekly, but at least once a month

What is the main electronic vaping device by which you use mephedrone?

- ○ Commercially bought electronic or e-cigarette
- ○ Commercially bought electronic device used to vaporise dry herbs
- ○ Commercially bought tabletop vapouriser
- ○ Commercially bought electronic device used to vape liquids (but not an e-cigarette or a tabletop vapouriser)
- ○ Homemade electronic device
- ○ Other, please describe

Are there any other electronic vaping devices by which you use mephedrone?

- ☐ Commercially bought electronic or e-cigarette
- ☐ Commercially bought electronic device used to vaporise dry herbs
- ☐ Commercially bought tabletop vapouriser
- ☐

For peer review only - http://bmjopen.bmj.com/site/about/guidelines.xhtml

Commercially bought electronic device used to vape liquids (but not an e-cigarette or a tabletop vapouriser)

- [ ] Homemade electronic device
- [ ] Other, please describe

Vaping DMT

You said that you had vaped DMT. What type of DMT have you vaped? (tick all that apply)

- [ ] DMT
- [ ] Other, please describe

How old were you the first time you ever used DMT? (by ANY method not only using an electronic vaping device)

What was the first route of administration by which you ever used DMT?

- ○ Vaping
- ○ Smoking
- ○ Oral (ingesting by mouth)
- ○ Intravenous (injecting into a vein)
- ○ Sniffing/snorting (taking by nose)
- ○ Other, please describe

How old were you when you the first time you ever vaped DMT?

What was your main reason for vaping DMT the first time?

- ○ To quit other routes of administration
- ○ To cut down other routes of administration
- ○ To use when I could not or was not allowed to use via other routes of administration
- ○ To avoid returning to other routes of administration
- ○ Because I thought I would enjoy it

For peer review only - http://bmjopen.bmj.com/site/about/guidelines.xhtml

- Because I though it would give me a more pleasurable effect than other routes
- Because they are less harmful than other routes of administration
- Because I was concerned about my health
- Curiosity just wanted to try them
- To save money
- To protect others from second-hand exposure
- For some other reason -please explain
- Don't know
- Prefer not to say

What were any other reasons for vaping DMT the first time?

- To quit other routes of administration
- To cut down other routes of administration
- To use when I cannot or am not allowed to use via other routes of administration
- To avoid returning to other routes of administration
- Because I enjoy it
- Because it gives me a more pleasurable effect than other routes
- Because they are less harmful than other routes of administration
- Because I was concerned about my health
- Curiosity just wanted to try them
- To save money
- To protect others from second-hand exposure
- For some other reason -please explain
- Don't know
- Prefer not to say

Have you used DMT in the last 30 days?

- Yes
- No

Past 30 DMT

Do you think your use of DMT is having a negative effect on your mental or physical health?

- Yes, on both my mental and physical health
- Yes, on my physical health only

For peer review only - http://bmjopen.bmj.com/site/about/guidelines.xhtml

- Yes, on my mental health only
- No

Which of the following best describes the frequency you used DMT using any route of administration in the last 30 days?

- Daily
- Less than daily, but at least once a week
- Less than weekly, but at least once a month

Do you have any current intention to quit using DMT?

- Yes
- No

Have you used an electronic vaping device to use DMT in the last 30 days?

- Yes
- No

Current Use DMT

What is your main reason for vaping DMT now?

- To quit other routes of administration
- To cut down other routes of administration
- To use when I cannot or am not allowed to use via other routes of administration
- To avoid returning to other routes of administration
- Because I enjoy it
- Because it gives me a more pleasurable effect than other routes
- Because they are less harmful than other routes of administration
- Because I was concerned about my health
- Curiosity just wanted to try them
- To save money
- To protect others from second-hand exposure
- For some other reason -please explain
- Don't know
- Prefer not to say

For peer review only - http://bmjopen.bmj.com/site/about/guidelines.xhtml

What are any other reasons for vaping DMT now?

- ☐ To quit other routes of administration
- ☐ To cut down other routes of administration
- ☐ To use when I cannot or am not allowed to use via other routes of administration
- ☐ To avoid returning to other routes of administration
- ☐ Because I enjoy it
- ☐ Because it gives me a more pleasurable effect than other routes
- ☐ Because they are less harmful than other routes of administration
- ☐ Because I was concerned about my health
- ☐ Curiosity just wanted to try them
- ☐ J. To save money
- ☐ To protect others from second-hand exposure
- ☐ For some other reason -please explain
- ☐ Don't know
- ☐ Prefer not to say

Which of the following best describes the frequency you vaped DMT in the last 30 days?

- ○ Daily
- ○ Less than daily, but at least once a week
- ○ Less than weekly, but at least once a month

What is the main electronic vaping device by which you use DMT?

- ○ Commercially bought electronic or e-cigarette
- ○ Commercially bought electronic device used to vaporise dry herbs
- ○ Commercially bought tabletop vapouriser
- ○ Commercially bought electronic device used to vape liquids (but not an e-cigarette or a tabletop vapouriser)
- ○ Homemade electronic device
- ○ Other, please describe

Are there any other electronic vaping devices by which you use DMT?

- ☐ Commercially bought electronic or e-cigarette
- ☐ Commercially bought electronic device used to vaporise dry herbs
- ☐

For peer review only - http://bmjopen.bmj.com/site/about/guidelines.xhtml

Commercially bought tabletop vapouriser

- [ ] Commercially bought electronic device used to vape liquids (but not an e-cigarette or a tabletop vapouriser)
- [ ] Homemade electronic device
- [ ] Other, please describe

Vaping Psilocybin

You said that you had vaped psilocybin. What type of psilocybin have you vaped? (tick all that apply)

- [ ] Psilocybin
- [ ] Other, please describe

How old were you the first time you ever used psilocybin? (by ANY method not only using an electronic vaping device)

What was the first route of administration by which you ever used psilocybin?

- Vaping
- Smoking
- Oral (ingesting by mouth)
- Intravenous (injecting into a vein)
- Sniffing/snorting (taking by nose)
- Other, please describe

How old were you when you the first time you ever vaped psilocybin?

What was your main reason for vaping psilocybin the first time?

- To quit other routes of administration
- To cut down other routes of administration
- To use when I cannot or am not allowed to use via other routes of administration
- To avoid returning to other routes of administration

- Because I enjoy it
- Because it gives me a more pleasurable effect than other routes
- Because they are less harmful than other routes of administration
- Because I was concerned about my health
- Curiosity just wanted to try them
- To save money
- To protect others from second-hand exposure
- For some other reason -please explain
- Don't know
- Prefer not to say

What were any other reasons for vaping psilocybin the first time?

- To quit other routes of administration
- To cut down other routes of administration
- To use when I cannot or am not allowed to use via other routes of administration
- To avoid returning to other routes of administration
- Because I enjoy it
- Because it gives me a more pleasurable effect than other routes
- Because they are less harmful than other routes of administration
- Because I was concerned about my health
- Curiosity just wanted to try them
- To save money
- To protect others from second-hand exposure
- For some other reason -please explain
- Don't know
- Prefer not to say

Have you used psilocybin in the last 30 days?

- Yes
- No

Past 30 psilocybin

Do you think your use of psilocybin is having a negative effect on your mental or physical health?

- Yes, on both my mental and physical health

For peer review only - http://bmjopen.bmj.com/site/about/guidelines.xhtml

- Yes, on my physical health only
- Yes, on my mental health only
- No

Which of the following best describes the frequency you used psilocybin using any route of administration in the last 30 days?

- Daily
- Less than daily, but at least once a week
- Less than weekly, but at least once a month

Do you have any current intention to quit using psilocybin?

- Yes
- No

Have you used an electronic vaping device to use psilocybin in the last 30 days?

- Yes
- No

Current use psilocybin

What is your main reason for vaping psilocybin now?

- To quit other routes of administration
- To cut down other routes of administration
- To use when I cannot or am not allowed to use via other routes of administration
- To avoid returning to other routes of administration
- Because I enjoy it
- Because it gives me a more pleasurable effect than other routes
- Because they are less harmful than other routes of administration
- Because I was concerned about my health
- Curiosity just wanted to try them
- To save money
- To protect others from second-hand exposure
- For some other reason -please explain
- Don't know

Prefer not to say

What are any other reasons for vaping psilocybin now?

- To quit other routes of administration
- To cut down other routes of administration
- To use when I cannot or am not allowed to use via other routes of administration
- To avoid returning to other routes of administration
- Because I enjoy it
- Because it gives me a more pleasurable effect than other routes
- Because they are less harmful than other routes of administration
- Because I was concerned about my health
- Curiosity just wanted to try them
- J. To save money
- To protect others from second-hand exposure
- For some other reason -please explain
- Don't know
- Prefer not to say

Which of the following best describes the frequency you vaped psilocybin in the last 30 days?

- Daily
- Less than daily, but at least once a week
- Less than weekly, but at least once a month

What is the main electronic vaping device by which you use psilocybin?

- Commercially bought electronic or e-cigarette
- Commercially bought electronic device used to vaporise dry herbs
- Commercially bought tabletop vapouriser
- Commercially bought electronic device used to vape liquids (but not an e-cigarette or a tabletop vapouriser)
- Homemade electronic device
- Other, please describe

Are there any other electronic vaping devices by which you use psilocybin?

- Commercially bought electronic or e-cigarette

For peer review only - http://bmjopen.bmj.com/site/about/guidelines.xhtml

- [ ] Commercially bought electronic device used to vaporise dry herbs
- [ ] Commercially bought tabletop vapouriser
- [ ] Commercially bought electronic device used to vape liquids (but not an e-cigarette or a tabletop vapouriser)
- [ ] Homemade electronic device
- [ ] Other, please describe

Vaping Benzodiazepines

You said that you had vaped benzodiazepines. What type of benzodiazepines have you vaped? (tick all that apply)

- [ ] Diazepam (aka blues, Valium)
- [ ] Clonazepam (aka Rivotril)
- [ ] Alprazolam (aka Xanax)
- [ ] Other, please describe

How old were you the first time you ever used benzodiazepines? (by ANY method not only using an electronic vaping device)

What was the first route of administration by which you ever used benzodiazepines?

- Vaping
- Smoking
- Oral (ingesting by mouth)
- Intravenous (injecting into a vein)
- Sniffing/snorting (taking by nose)
- Other, please describe

How old were you when you the first time you ever vaped benzodiazepines?

What was your main reason for vaping benzodiazepines the first time?

- To quit other routes of administration

For peer review only - http://bmjopen.bmj.com/site/about/guidelines.xhtml

- To cut down other routes of administration
- To use when I could not or was not allowed to use via other routes of administration
- To avoid returning to other routes of administration
- Because I thought I would enjoy it
- Because I though it would give me a more pleasurable effect than other routes
- Because they are less harmful than other routes of administration
- Because I was concerned about my health
- Curiosity just wanted to try them
- To save money
- To protect others from second-hand exposure
- For some other reason -please explain
- Don't know
- Prefer not to say

What were any other reasons for vaping benzodiazepines the first time?

- To quit other routes of administration
- To cut down other routes of administration
- To use when I cannot or am not allowed to use via other routes of administration
- To avoid returning to other routes of administration
- Because I enjoy it
- Because it gives me a more pleasurable effect than other routes
- Because they are less harmful than other routes of administration
- Because I was concerned about my health
- Curiosity just wanted to try them
- To save money
- To protect others from second-hand exposure
- For some other reason -please explain
- Don't know
- Prefer not to say

Have you used benzodiazepines in the last 30 days?

- Yes
- No

Past 30 benzodiazepines

For peer review only - http://bmjopen.bmj.com/site/about/guidelines.xhtml

Do you think your use of benzodiazepines is having a negative effect on your mental or physical health?

- Yes, on both my mental and physical health
- Yes, on my physical health only
- Yes, on my mental health only
- No

Which of the following best describes the frequency you used benzodiazepines using any route of administration in the last 30 days?

- Daily
- Less than daily, but at least once a week
- Less than weekly, but at least once a month

Do you have any current intention to quit using benzodiazepines?

- Yes
- No

Have you used an electronic vaping device to use benzodiazepines in the last 30 days?

- Yes
- No

Current use benzodiazepines

What is your main reason for vaping benzodiazepines now?

- To quit other routes of administration
- To cut down other routes of administration
- To use when I cannot or am not allowed to use via other routes of administration
- To avoid returning to other routes of administration
- Because I enjoy it
- Because it gives me a more pleasurable effect than other routes
- Because they are less harmful than other routes of administration
- Because I was concerned about my health
- Curiosity just wanted to try them
- To save money
- To protect others from second-hand exposure

For peer review only - http://bmjopen.bmj.com/site/about/guidelines.xhtml

- For some other reason -please explain
- Don't know
- Prefer not to say

What are any other reasons for vaping benzodiazepines now?

- To quit other routes of administration
- To cut down other routes of administration
- To use when I cannot or am not allowed to use via other routes of administration
- To avoid returning to other routes of administration
- Because I enjoy it
- Because it gives me a more pleasurable effect than other routes
- Because they are less harmful than other routes of administration
- Because I was concerned about my health
- Curiosity just wanted to try them
- To save money
- To protect others from second-hand exposure
- For some other reason -please explain
- Don't know
- Prefer not to say

Which of the following best describes the frequency you vaped benzodiazepines in the last 30 days?

- Daily
- Less than daily, but at least once a week
- Less than weekly, but at least once a month

What is the main electronic vaping device by which you use benzodiazepines?

- Commercially bought electronic or e-cigarette
- Commercially bought electronic device used to vaporise dry herbs
- Commercially bought tabletop vapouriser
- Commercially bought electronic device used to vape liquids (but not an e-cigarette or a tabletop vapouriser)
- Homemade electronic device
- Other, please describe

Are there any other electronic vaping devices by which you use benzodiazepines?

- ☐ Commercially bought electronic or e-cigarette
- ☐ Commercially bought electronic device used to vaporise dry herbs
- ☐ Commercially bought tabletop vapouriser
- ☐ Commercially bought electronic device used to vape liquids (but not an e-cigarette or a tabletop vapouriser)
- ☐ Homemade electronic device
- ☐ Other, please describe

Vaping 2C

You said that you had vaped 2C. What type of 2C have you vaped? (tick all that apply)

- ☐ 2CB
- ☐ 2CE
- ☐ Other, please describe

How old were you the first time you ever used 2C? (by ANY method not only using an electronic vaping device)

What was the first route of administration by which you ever used 2C?

- ○ Vaping
- ○ Smoking
- ○ Oral (ingesting by mouth)
- ○ Intravenous (injecting into a vein)
- ○ Sniffing/snorting (taking by nose)
- ○ Other, please describe

How old were you when you the first time you ever vaped 2C?

What was your main reason for vaping 2C the first time?

- To quit other routes of administration
- To cut down other routes of administration
- To use when I could not or was not allowed to use via other routes of administration
- To avoid returning to other routes of administration
- Because I thought I would enjoy it
- Because I though it would give me a more pleasurable effect than other routes
- Because they are less harmful than other routes of administration
- Because I was concerned about my health
- Curiosity just wanted to try them
- To save money
- To protect others from second-hand exposure
- For some other reason -please explain
- Don't know
- Prefer not to say

What were any other reasons for vaping 2C the first time?

- To quit other routes of administration
- To cut down other routes of administration
- To use when I cannot or am not allowed to use via other routes of administration
- To avoid returning to other routes of administration
- Because I enjoy it
- Because it gives me a more pleasurable effect than other routes
- Because they are less harmful than other routes of administration
- Because I was concerned about my health
- Curiosity just wanted to try them
- To save money
- To protect others from second-hand exposure
- For some other reason -please explain
- Don't know
- Prefer not to say

Have you used 2C in the last 30 days?

- Yes
- No

Past 30 2C

Do you think your use of 2C is having a negative effect on your mental or physical health?

- Yes, on both my mental and physical health
- Yes, on my physical health only
- Yes, on my mental health only
- No

Which of the following best describes the frequency you used 2C using any route of administration in the last 30 days?

- Daily
- Less than daily, but at least once a week
- Less than weekly, but at least once a month

Do you have any current intention to quit using 2C?

- Yes
- No

Have you used an electronic vaping device to use 2C in the last 30 days?

- Yes
- No

Current use 2C

What is your main reason for vaping 2C now?

- To quit other routes of administration
- To cut down other routes of administration
- To use when I cannot or am not allowed to use via other routes of administration
- To avoid returning to other routes of administration
- Because I enjoy it
- Because it gives me a more pleasurable effect than other routes
- Because they are less harmful than other routes of administration
- Because I was concerned about my health
- Curiosity just wanted to try them
- To save money

For peer review only - http://bmjopen.bmj.com/site/about/guidelines.xhtml

- To protect others from second-hand exposure
- For some other reason -please explain
- Don't know
- Prefer not to say

What are any other reasons for vaping 2C now?

- To quit other routes of administration
- To cut down other routes of administration
- To use when I cannot or am not allowed to use via other routes of administration
- To avoid returning to other routes of administration
- Because I enjoy it
- Because it gives me a more pleasurable effect than other routes
- Because they are less harmful than other routes of administration
- Because I was concerned about my health
- Curiosity just wanted to try them
- J. To save money
- To protect others from second-hand exposure
- For some other reason -please explain
- Don't know
- Prefer not to say

Which of the following best describes the frequency you vaped 2C in the last 30 days?

- Daily
- Less than daily, but at least once a week
- Less than weekly, but at least once a month

What is the main electronic vaping device by which you use 2C?

- Commercially bought electronic or e-cigarette
- Commercially bought electronic device used to vaporise dry herbs
- Commercially bought tabletop vapouriser
- Commercially bought electronic device used to vape liquids (but not an e-cigarette or a tabletop vapouriser)
- Homemade electronic device
- Other, please describe

Are there any other electronic vaping devices by which you use 2C?

- ☐ Commercially bought electronic or e-cigarette
- ☐ Commercially bought electronic device used to vaporise dry herbs
- ☐ Commercially bought tabletop vapouriser
- ☐ Commercially bought electronic device used to vape liquids (but not an e-cigarette or a tabletop vapouriser)
- ☐ Homemade electronic device
- ☐ Other, please describe

Vaping alpha-PVP

You said that you had vaped alpha-PVP. What type of alpha-PVP have you vaped? (tick all that apply)

- ☐ alpha-PVP (aka gravel, flakka)
- ☐ Other, please describe

How old were you the first time you ever used alpha-PVP? (by ANY method not only using an electronic vaping device)

What was the first route of administration by which you ever used alpha-PVP?

- ○ Vaping
- ○ Smoking
- ○ Oral (ingesting by mouth)
- ○ Intravenous (injecting into a vein)
- ○ Sniffing/snorting (taking by nose)
- ○ Other, please describe

How old were you when you the first time you ever vaped alpha-PVP?

What was your main reason for vaping alpha-PVP the first time?

For peer review only - http://bmjopen.bmj.com/site/about/guidelines.xhtml

- To quit other routes of administration
- To cut down other routes of administration
- To use when I could not or was not allowed to use via other routes of administration
- To avoid returning to other routes of administration
- Because I thought I would enjoy it
- Because I though it would give me a more pleasurable effect than other routes
- Because they are less harmful than other routes of administration
- Because I was concerned about my health
- Curiosity just wanted to try them
- To save money
- To protect others from second-hand exposure
- For some other reason -please explain
- Don't know
- Prefer not to say

What were any other reasons for vaping alpha-PVP the first time?

- To quit other routes of administration
- To cut down other routes of administration
- To use when I cannot or am not allowed to use via other routes of administration
- To avoid returning to other routes of administration
- Because I enjoy it
- Because it gives me a more pleasurable effect than other routes
- Because they are less harmful than other routes of administration
- Because I was concerned about my health
- Curiosity just wanted to try them
- To save money
- To protect others from second-hand exposure
- For some other reason -please explain
- Don't know
- Prefer not to say

Have you used alpha-PVP in the last 30 days?

- Yes
- No

For peer review only - http://bmjopen.bmj.com/site/about/guidelines.xhtml

Past 30 alpha-PVP

Do you think your use of alpha-PVP is having a negative effect on your mental or physical health?

- Yes, on both my mental and physical health
- Yes, on my physical health only
- Yes, on my mental health only
- No

Which of the following best describes the frequency you used alpha-PVP using any route of administration in the last 30 days?

- Daily
- Less than daily, but at least once a week
- Less than weekly, but at least once a month

Do you have any current intention to quit using alpha-PVP?

- Yes
- No

Have you used an electronic vaping device to use alpha-PVP in the last 30 days?

- Yes
- No

Current use alpha-PVP

What is your main reason for vaping alpha-PVP now?

- To quit other routes of administration
- To cut down other routes of administration
- To use when I cannot or am not allowed to use via other routes of administration
- To avoid returning to other routes of administration
- Because I enjoy it
- Because it gives me a more pleasurable effect than other routes
- Because they are less harmful than other routes of administration
- Because I was concerned about my health
- Curiosity just wanted to try them
- To save money

For peer review only - http://bmjopen.bmj.com/site/about/guidelines.xhtml

- To protect others from second-hand exposure
- For some other reason -please explain
- Don't know
- Prefer not to say

What are any other reasons for vaping alpha-PVP now?

- To quit other routes of administration
- To cut down other routes of administration
- To use when I cannot or am not allowed to use via other routes of administration
- To avoid returning to other routes of administration
- Because I enjoy it
- Because it gives me a more pleasurable effect than other routes
- Because they are less harmful than other routes of administration
- Because I was concerned about my health
- Curiosity just wanted to try them
- To save money
- To protect others from second-hand exposure
- For some other reason -please explain
- Don't know
- Prefer not to say

Which of the following best describes the frequency you vaped alpha-PVP in the last 30 days?

- Daily
- Less than daily, but at least once a week
- Less than weekly, but at least once a month

What is the main electronic vaping device by which you use alpha-PVP?

- Commercially bought electronic or e-cigarette
- Commercially bought electronic device used to vaporise dry herbs
- Commercially bought tabletop vapouriser
- Commercially bought electronic device used to vape liquids (but not an e-cigarette or a tabletop vapouriser)
- Homemade electronic device
- Other, please describe

For peer review only - http://bmjopen.bmj.com/site/about/guidelines.xhtml

Are there any other electronic vaping devices by which you use alpha-PVP?

- ☐ Commercially bought electronic or e-cigarette
- ☐ Commercially bought electronic device used to vaporise dry herbs
- ☐ Commercially bought tabletop vapouriser
- ☐ Commercially bought electronic device used to vape liquids (but not an e-cigarette or a tabletop vapouriser)
- ☐ Homemade electronic device
- ☐ Other, please describe

Vaping NBOMe

You said that you had vaped NBOMe What type of NBOMe have you vaped? (tick all that apply)

- ☐ NBOMe (aka N-Bomb)
- ☐ Other, please describe

How old were you the first time you ever used NBOMe? (by ANY method not only using an electronic vaping device)

What was the first route of administration by which you ever used NBOMe?

- ○ Vaping
- ○ Smoking
- ○ Oral (ingesting by mouth)
- ○ Intravenous (injecting into a vein)
- ○ Sniffing/snorting (taking by nose)
- ○ Other, please describe

How old were you when you the first time you ever vaped NBOMe?

What was your main reason for vaping NBOMe the first time?

For peer review only - http://bmjopen.bmj.com/site/about/guidelines.xhtml

- To quit other routes of administration
- To cut down other routes of administration
- To use when I could not or was not allowed to use via other routes of administration
- To avoid returning to other routes of administration
- Because I thought I would enjoy it
- Because I though it would give me a more pleasurable effect than other routes
- Because they are less harmful than other routes of administration
- Because I was concerned about my health
- Curiosity just wanted to try them
- To save money
- To protect others from second-hand exposure
- For some other reason -please explain
- Don't know
- Prefer not to say

What were any other reasons for vaping NBOMe the first time?

- To quit other routes of administration
- To cut down other routes of administration
- To use when I cannot or am not allowed to use via other routes of administration
- To avoid returning to other routes of administration
- Because I enjoy it
- Because it gives me a more pleasurable effect than other routes
- Because they are less harmful than other routes of administration
- Because I was concerned about my health
- Curiosity just wanted to try them
- To save money
- To protect others from second-hand exposure
- For some other reason -please explain
- Don't know
- Prefer not to say

Have you used NBOMe in the last 30 days?

- Yes
- No

Past 30 NBOMe

Do you think your use of NBOMe is having a negative effect on your mental or physical health?

- Yes, on both my mental and physical health
- Yes, on my physical health only
- Yes, on my mental health only
- No

Which of the following best describes the frequency you used NBOMe using any route of administration in the last 30 days?

- Daily
- Less than daily, but at least once a week
- Less than weekly, but at least once a month

Do you have any current intention to quit using NBOMe?

- Yes
- No

Have you used an electronic vaping device to use NBOMe in the last 30 days?

- Yes
- No

Current use NBOMe

What is your main reason for vaping NBOMe now?

- To quit other routes of administration
- To cut down other routes of administration
- To use when I cannot or am not allowed to use via other routes of administration
- To avoid returning to other routes of administration
- Because I enjoy it
- Because it gives me a more pleasurable effect than other routes
- Because they are less harmful than other routes of administration
- Because I was concerned about my health
- Curiosity just wanted to try them
- To save money

For peer review only - http://bmjopen.bmj.com/site/about/guidelines.xhtml

- To protect others from second-hand exposure
- For some other reason -please explain
- Don't know
- Prefer not to say

What are any other reasons for vaping NBOMe now?

- To quit other routes of administration
- To cut down other routes of administration
- To use when I cannot or am not allowed to use via other routes of administration
- To avoid returning to other routes of administration
- Because I enjoy it
- Because it gives me a more pleasurable effect than other routes
- Because they are less harmful than other routes of administration
- Because I was concerned about my health
- Curiosity just wanted to try them
- To save money
- To protect others from second-hand exposure
- For some other reason -please explain
- Don't know
- Prefer not to say

Which of the following best describes the frequency you vaped NBOMe in the last 30 days?

- Daily
- Less than daily, but at least once a week
- Less than weekly, but at least once a month

What is the main electronic vaping device by which you use NBOMe?

- Commercially bought electronic or e-cigarette
- Commercially bought electronic device used to vaporise dry herbs
- Commercially bought tabletop vapouriser
- Commercially bought electronic device used to vape liquids (but not an e-cigarette or a tabletop vapouriser)
- Homemade electronic device
- Other, please describe

Are there any other electronic vaping devices by which you use NBOMe?

- Commercially bought electronic or e-cigarette
- Commercially bought electronic device used to vaporise dry herbs
- Commercially bought tabletop vapouriser
- Commercially bought electronic device used to vape liquids (but not an e-cigarette or a tabletop vapouriser)
- Homemade electronic device
- Other, please describe

Vaping Other

You said that you had vaped other substances. What type of other substances have you vaped? (tick all that apply)

How old were you the first time you ever used these other substances? (by ANY method not only using an electronic vaping device)

What was the first route of administration by which you ever used these other substances?

- Vaping
- Smoking
- Oral (ingesting by mouth)
- Intravenous (injecting into a vein)
- Sniffing/snorting (taking by nose)
- Other, please describe

How old were you when you the first time you ever vaped these other substances?

What was your main reason for vaping these other substances the first time?

For peer review only - http://bmjopen.bmj.com/site/about/guidelines.xhtml

- To quit other routes of administration
- To cut down other routes of administration
- To use when I could not or was not allowed to use via other routes of administration
- To avoid returning to other routes of administration
- Because I thought I would enjoy it
- Because I though it would give me a more pleasurable effect than other routes
- Because they are less harmful than other routes of administration
- Because I was concerned about my health
- Curiosity just wanted to try them
- To save money
- To protect others from second-hand exposure
- For some other reason -please explain
- Don't know
- Prefer not to say

What were any other reasons for vaping these other substances the first time?

- To quit other routes of administration
- To cut down other routes of administration
- To use when I cannot or am not allowed to use via other routes of administration
- To avoid returning to other routes of administration
- Because I enjoy it
- Because it gives me a more pleasurable effect than other routes
- Because they are less harmful than other routes of administration
- Because I was concerned about my health
- Curiosity just wanted to try them
- To save money
- To protect others from second-hand exposure
- For some other reason -please explain
- Don't know
- Prefer not to say

Have you used these other substances in the last 30 days?

- Yes
- No

For peer review only - http://bmjopen.bmj.com/site/about/guidelines.xhtml

Past 30 other substance

Do you think your use of other substances is having a negative effect on your mental or physical health?

- Yes, on both my mental and physical health
- Yes, on my physical health only
- Yes, on my mental health only
- No

Which of the following best describes the frequency you used other substances using any route of administration in the last 30 days?

- Daily
- Less than daily, but at least once a week
- Less than weekly, but at least once a month

Do you have any current intention to quit using other substances?

- Yes
- No

Have you used an electronic vaping device to use other substances in the last 30 days?

- Yes
- No

Current use other substances

What is your main reason for vaping other substances now?

- To quit other routes of administration
- To cut down other routes of administration
- To use when I cannot or am not allowed to use via other routes of administration
- To avoid returning to other routes of administration
- Because I enjoy it
- Because it gives me a more pleasurable effect than other routes
- Because they are less harmful than other routes of administration
- Because I was concerned about my health
- Curiosity just wanted to try them
- To save money

For peer review only - http://bmjopen.bmj.com/site/about/guidelines.xhtml

- To protect others from second-hand exposure
- For some other reason -please explain
- Don't know
- Prefer not to say

What are any other reasons for vaping other substances now?

- To quit other routes of administration
- To cut down other routes of administration
- To use when I cannot or am not allowed to use via other routes of administration
- To avoid returning to other routes of administration
- Because I enjoy it
- Because it gives me a more pleasurable effect than other routes
- Because they are less harmful than other routes of administration
- Because I was concerned about my health
- Curiosity just wanted to try them
- To save money
- To protect others from second-hand exposure
- For some other reason -please explain
- Don't know
- Prefer not to say

Which of the following best describes the frequency you vaped other substances in the last 30 days?

- Daily
- Less than daily, but at least once a week
- Less than weekly, but at least once a month

What is the main electronic vaping device by which you use other substances ?

- Commercially bought electronic or e-cigarette
- Commercially bought electronic device used to vaporise dry herbs
- Commercially bought tabletop vapouriser
- Commercially bought electronic device used to vape liquids (but not an e-cigarette or a tabletop vapouriser)
- Homemade electronic device
- Other, please describe

For peer review only - http://bmjopen.bmj.com/site/about/guidelines.xhtml

Are there any other electronic vaping devices by which you use other substances ?

- ☐ Commercially bought electronic or e-cigarette
- ☐ Commercially bought electronic device used to vaporise dry herbs
- ☐ Commercially bought tabletop vapouriser
- ☐ Commercially bought electronic device used to vape liquids (but not an e-cigarette or a tabletop vapouriser)
- ☐ Homemade electronic device
- ☐ Other, please describe

Demographics

What is your age?

- ○ 18-24
- ○ 25-34
- ○ 35-44
- ○ 45-54
- ○ 55-64
- ○ 65-74
- ○ over 75

What is your sex?
Please use the sex recorded on your birth certificate or Gender Recognition Certificate

- ○ Female
- ○ Male
- ○ Prefer not to say

Is the gender you identify with the same as your sex registered at birth?

- ○ Yes
- ○ No, please give the term you use to describe your gender
- ○ Do not wish to say

What is your ethnic group?
Please choose one option that best describes your ethnic group or background

- ○

For peer review only - http://bmjopen.bmj.com/site/about/guidelines.xhtml

English/Welsh/Scottish/Northern Irish/British

- Irish
- Gypsy or Irish Traveller
- Any other White background, please describe
- White and Black Caribbean
- White and Black African
- White and Asian
- Any other Mixed/Multiple ethnic background, please describe
- Indian
- Pakistani
- Bangladeshi
- Chinese
- Any other Asian background, please describe
- African
- Caribbean
- Any other Black/African/Caribbean background, please describe
- Arab
- Any other ethnic group, please describe
- Do not wish to say

What is your religion?

- No religion
- Christian (including Church of England, Catholic, Protestant and all other Christian denominations)
- Buddhist
- Hindu
- Jewish
- Muslim
- Sikh
- Any other religion, please describe
- Do not wish to say

What is your legal marital or registered civil partnership status?

- Never married and never registered in a civil partnership

For peer review only - http://bmjopen.bmj.com/site/about/guidelines.xhtml

Married

- In a registered civil partnership
- Separated, but still legally married
- Separated, but still legally in a civil partnership
- Divorced
- Formerly in a civil partnership which is now legally dissolved
- Widowed
- Surviving partner from a registered civil partnership
- Do not wish to say

Which of the following best describes your sexual orientation?

- Straight/Heterosexual
- Gay or Lesbian
- Bisexual
- Other sexual orientation, please give the term you use to describe your sexual orientation
- Do not wish to say

Have you achieved a qualification at degree level or above?

- Yes
- No
- Do not wish to say

Do you have any physical or mental health conditions or illnesses lasting or expected to last 12 months or more?

- Yes
- No
- Do not wish to say

Do any of your conditions or illnesses reduce your ability to carry out day-to-day activities?

- Yes, a lot
- Yes, a little
- Not at all

For peer review only - http://bmjopen.bmj.com/site/about/guidelines.xhtml

For peer review only

For peer review only - http://bmjopen.bmj.com/site/about/guidelines.xhtml

STROBE Statement—Checklist of items that should be included in reports of ***cross-sectional studies***

	Item No	Recommendation	Page No
**Title and abstract**	1	(*a*) Indicate the study's design with a commonly used term in the title or the abstract	1
		(*b*) Provide in the abstract an informative and balanced summary of what was done and what was found	3
**Introduction**			
Background/rationale	2	Explain the scientific background and rationale for the investigation being reported	5
Objectives	3	State specific objectives, including any prespecified hypotheses	5
**Methods**			
Study design	4	Present key elements of study design early in the paper	6
Setting	5	Describe the setting, locations, and relevant dates, including periods of recruitment, exposure, follow-up, and data collection	6
Participants	6	(*a*) Give the eligibility criteria, and the sources and methods of selection of participants	6
Variables	7	Clearly define all outcomes, exposures, predictors, potential confounders, and effect modifiers. Give diagnostic criteria, if applicable	6
Data sources/ measurement	8*	For each variable of interest, give sources of data and details of methods of assessment (measurement). Describe comparability of assessment methods if there is more than one group	*6*
Bias	9	Describe any efforts to address potential sources of bias	6
Study size	10	Explain how the study size was arrived at	6/7
Quantitative variables	11	Explain how quantitative variables were handled in the analyses. If applicable, describe which groupings were chosen and why	6
Statistical methods	12	(*a*) Describe all statistical methods, including those used to control for confounding	6
		(*b*) Describe any methods used to examine subgroups and interactions	6
		(*c*) Explain how missing data were addressed	N/A
		(*d*) If applicable, describe analytical methods taking account of sampling strategy	6
		(*e*) Describe any sensitivity analyses	N/A
**Results**			
Participants	13*	(a) Report numbers of individuals at each stage of study—eg numbers potentially eligible, examined for eligibility, confirmed eligible, included in the study, completing follow-up, and analysed	7
		(b) Give reasons for non-participation at each stage	N/A
		(c) Consider use of a flow diagram	N/A
Descriptive data	14*	(a) Give characteristics of study participants (eg demographic, clinical, social) and information on exposures and potential confounders	7
		(b) Indicate number of participants with missing data for each variable of interest	N/A
Outcome data	15*	Report numbers of outcome events or summary measures	7
Main results	16	(*a*) Give unadjusted estimates and, if applicable, confounder-adjusted estimates and their precision (eg, 95% confidence interval). Make clear which confounders were adjusted for and why they were included	7

For peer review only - http://bmjopen.bmj.com/site/about/guidelines.xhtml

		(*b*) Report category boundaries when continuous variables were categorized	N/A
		(*c*) If relevant, consider translating estimates of relative risk into absolute risk for a meaningful time period	N/A
Other analyses	17	Report other analyses done—eg analyses of subgroups and interactions, and sensitivity analyses	7
**Discussion**			
Key results	18	Summarise key results with reference to study objectives	9
Limitations	19	Discuss limitations of the study, taking into account sources of potential bias or imprecision. Discuss both direction and magnitude of any potential bias	10
Interpretation	20	Give a cautious overall interpretation of results considering objectives, limitations, multiplicity of analyses, results from similar studies, and other relevant evidence	9/10
Generalisability	21	Discuss the generalisability (external validity) of the study results	9/10
**Other information**			
Funding	22	Give the source of funding and the role of the funders for the present study and, if applicable, for the original study on which the present article is based	2

*Give information separately for exposed and unexposed groups.

Note: An Explanation and Elaboration article discusses each checklist item and gives methodological background and published examples of transparent reporting. The STROBE checklist is best used in conjunction with this article (freely available on the Web sites of PLoS Medicine at http://www.plosmedicine.org/, Annals of Internal Medicine at http://www.annals.org/, and Epidemiology at http://www.epidem.com/). Information on the STROBE Initiative is available at www.strobe-statement.org.

For peer review only

For peer review only - http://bmjopen.bmj.com/site/about/guidelines.xhtml
